# Supplementary material for: Economic system justification predicts muted emotional responses to inequality
Source: Nat Commun. 2020 Jan 20;11:383. doi: 10.1038/s41467-019-14193-z (PMC6971272; doi:10.1038/s41467-019-14193-z)
Supplement: Supplementary file 1 — Supplementary Information [file 41467_2019_14193_MOESM1_ESM.pdf]

Supplementary Information for

Economic System Justification Predicts Muted Emotional Responses to Inequality

Shahrzad Goudarzi
Ruthie Pliskin
John T. Jost
Eric D. Knowles

This PDF file includes:

Supplementary text
Supplementary references
Supplementary Figures 1–63
Supplementary Tables 1–85

Supplementary Information Text

Study 1

Procedure

U.S.-born workers from Amazon's Mechanical Turk crowdsourcing platform were recruited to complete a two-part online experiment. In the first session, participants provided informed consent and completed pretest survey that included demographic questions, the economic system justification (ESJ)[1] scale, and other exploratory measures. Participants were contacted at least a week later with a link to the second part of the experiment, during which they watched a control and a homeless video clip. The order of the videos was randomized between participants. After watching each video, participants answered two sets of questions gauging feelings toward the person in the video and toward the American socioeconomic system. Person-directed emotion questions always preceded the system-directed emotion items. Lastly, participants completed the Balanced Inventory of Desirable Responding (BIDR).[2] After completing the experiment, participants were debriefed, thanked, and paid $0.90.

Participants

Of the 147 participants originally recruited, 105 completed the second part of the experiment, and our analyses reflect only these individuals. The final sample consisted of 55 males and 50 females, aged 18 to 68 (M = 37.72, SD = 13.03). Eighty-six participants identified as White, 7 as Asian American, 7 as Black or African American, 1 as Latinx, 3 as more than one ethnicity, with 1 participant declining to answer the question.

Measures and Materials

Our independent variable consisted of scores on the 17-item ESJ scale, which gauges belief in the fairness of current socioeconomic arrangements in the United States. Sample items include: "Most people who don't get ahead in our society should not blame the system; they have only themselves to blame," "It is unfair to have an economic system which produces extreme wealth and extreme poverty at the same time" (reverse-scored), "There are many reasons to think that the economic system is unfair," and "Economic differences in the society reflect an illegitimate distribution of resources" (reverse-scored). Participants indicated their level of agreement on a scale from 1 ("Strongly Disagree") to 9 ("Strongly Agree") (α = 0.87). This measure was completed as part of the pretest survey in the first session.

The pretest survey also probed participants' gender, age, education level, ethnicity, political orientation, party affiliation, religiosity, citizenship status, country of birth, years lived in the U.S., and household income. For exploratory purposes, participants also completed measures of subjective socioeconomic status,[3] subjective social class,[4] general system justification,[5] Protestant Work Ethic,[6] and Belief in a Just World.[7]

For the dependent variables, measured during the second session, participants

reported how much anger, sadness, disgust, guilt, pity, and empathy they felt toward the person as they watched the video, and how much anger, sadness, disgust, guilt, pride, and hope they felt toward the American socioeconomic system as they watched the video, on a scale ranging from 0 to 100. We measured self-reported levels of discrete emotions because different discrete emotions are known to be associated with different attitudinal and behavioral responses. For instance, anger is related to punitive judgement and greater risk-taking, including active aggression, whereas sadness is associated with reduced risk-taking and more passive behavior (see[8]). Likewise, anger promotes approach and confrontation, whereas disgust promotes avoidance and expulsion.[9] Whereas pity is a paternalistic sympathetic emotion with ambivalent outcomes,[10] empathy is a non-paternalistic sympathetic emotion that is more clearly associated with helping behavior.[11] In contrast to the aforementioned emotions, guilt and pride are both self-directed emotions, but guilt involves attribution of responsibility for negative outcomes to the self, whereas pride involves attribution of positive outcomes to the self.[12] Accordingly, guilt is associated with reparative behavior,[13] and pride is associated with a sense of entitlement.[14] Finally, hope promotes action to achieve a goal that is deemed both desirable and feasible, but uncertain.[15, 16] These different behavioral outcomes mean that analyses that combine multiple emotions that share the same valence may fail to explain downstream differences in behavior and self-assessment.

For exploratory purposes, we also asked some memory questions for the content of the videos participants watched.

Finally, participants responded to the BIDR**,** a widely-used measure of biased responding on self-report instruments. The questionnaire includes two 20-item subscales: self-deceptive enhancement (SDE; $\alpha = 0.74$) and impression management (IM; $\alpha = 0.82$). Sample SDE items include, “I don’t care to know what other people really think of me” and “I am a completely rational person.” Sample IM items include, “I never take things that don't belong to me” and “I sometimes drive faster than the speed limit” (reverse-scored). Participants rated the items on a scale from 1 (“Not True”) to 7 (“Very True”). The BIDR was administered at the end of the second session after participants watched the videos and answered the emotion questions.

The videos shown to participants during the second session depicted interviews with homeless or non-homeless (control) persons. For each participant, a homeless video was randomly selected from a set of four videos (2 men and 2 women) and a control video was randomly selected from a set of two videos (1 man and 1 woman). Participants always saw the homeless video first. The individuals in all videos were White. The homeless videos showed individuals talking about their daily routines and the hardships of homelessness and living in poverty, and were edited to exclude references to crime or substance abuse. The videos were publicly available on the Internet and were identified by entering the keywords “homeless,” “interview,” and “video” into the Google search engine. The control videos (which originally aired on the C-SPAN television station) showed people talking about fishing and the process of making coffee. The homeless and control videos averaged 130 and 222 seconds in length, respectively; the shortest homeless video was 137 seconds long and the shortest control video was 126 seconds

long

Supplementary Results

Mixed-effects linear regressions were conducted to examine the effects of ESJ (z-scored), Video Type (1 = homeless, 0 = control), and the ESJ × Video Type interaction on affective reports. Intercepts varied randomly between participants and robust standard errors were specified. We adjusted for video order in the analysis, and stimuli were effect-coded within video type to adjust for within-condition heterogeneity between the videos.

After applying Bonferroni correction, we observed a significant ESJ × Video Type interaction effect on person-directed sadness and pity and system- directed anger, sadness, and disgust (Supplementary Tables 5–16). Analyses of simple effects* indicated that participants both high and low in ESJ reported higher levels of sadness and pity toward the person in the homeless as compared to the control video. However, this condition effect was larger for low-ESJ participants (sadness: $B = 72.726$, $SE\ B = 3.086$, $z = 23.569$, $p < .001$, 95% CI = [66.678, 78.774]; pity: $B = 67.87$, $SE\ B = 3.612$, $z = 18.79$, $p < .001$, 95% CI = [60.791, 74.95]) as compared to high-ESJ participants (sadness: $B = 54.46$, $SE\ B = 4.491$, $z = 12.126$, $p < .001$, 95% CI = [45.657, 63.262]; pity: $B = 46.775$, $SE\ B = 4.238$, $z = 11.036$, $p < .001$, 95% CI = [38.468, 55.082]).

Analyses of simple effects also indicated that participants both high and low in ESJ reported higher levels of sadness, anger, and disgust directed toward the American socioeconomic system in response to the homeless as compared to the control video. Again, the condition effect was larger for low-ESJ (anger: $B = 46.596$, $SE\ B = 4.769$, $z = 9.77$, $p < .001$, 95% CI = [37.248, 55.944]; sadness: $B = 54.558$, $SE\ B = 4.543$, $z = 12.009$, $p < .001$, 95 % CI = [45.654, 63.463]; disgust: $B = 50.012$, $SE\ B = 5.114$, $z = 9.780$, $p < .001$, 95 % CI = [39.989, 60.035]) as compared to high-ESJ (anger: $B = 25.924$, $SE\ B = 3.455$, $z = 7.503$, $p < .001$, 95 % CI = [19.152, 32.696]; sadness: $B = 35.39$, $SE\ B = 3.418$, $z = 10.355$, $p < .001$, 95 % CI = [28.691, 42.088]; disgust: $B = 23.369$, $SE\ B = 3.546$, $z = 6.59$, $p < .001$, 95 % CI = [16.418, 30.319]) participants.

Finally, in the homeless condition, the simple effect of ESJ on person-directed sadness and pity was significant (sadness: $B = -6.934$, $SE\ B = 2.364$, $z = -2.933$, $p = .003$, 95 % CI = [-11.568, -2.3]; pity: $B = -8.482$, $SE\ B = 2.686$, z = -3.158, $p = .002$, 95 % CI = [-13.747, -3.218]). Furthermore, in the homeless condition, the simple effect of ESJ on system-directed anger, sadness, and disgust was significant (anger: $B = -13.537$, $SE\ B = 2.429$, $z = -5.573$, $p < .001$, 95 % CI = [-18.298, -8.777]; sadness: $B = -10.607$, $SE\ B = 2.63$, $z = -4.034$, $p < .001$, 95 % CI = [-15.761, -5.454]; disgust: $B = -17.083$, $SE\ B = 2.355$, z = -7.255, $p < .001$, 95 % CI = [-21.698, -12.468]).

* Here, we only report simple slopes analyses for those dependent variables for which the ESJ x Video Type interaction effect was statistically significant (after applying Bonferroni correction).

Study 2

Procedure

U.S.-born workers from the Prolific Academic crowdsourcing platform were recruited to complete a two-part online experiment similar to Study 1. In the first session, participants provided informed consent and completed the ESJ scale. Participants were contacted about a week later and were invited to participate in the second session, during which they watched a control and a homeless video clip (previously used in Study 1) as well as a cystic fibrosis (CF) video. The order of the presentation of the videos was randomized between participants. After watching each video, participants answered two sets of questions that measured evoked feelings toward the person in the video and toward the American socioeconomic system. Finally, participants completed the BIDR scale and empathic concern measure. Participants were then debriefed and thanked for their participation. Participants were paid \$0.90 for completing the first session and \$1.70 for completing the second session.

Participants

Of the 400 participants recruited for the first session of the experiment, 326 completed the second session, and our analyses reflect only these individuals. The final sample consisted of 151 men, 171 women, and 4 who identified as non-binary, aged 18 to 72 (M = 33.80, SD = 11.77). Two hundred and forty-four participants identified as White, 20 as East Asian or Asian American, 22 as Black or African American, 14 as Latinx, 2 as Native American, 23 as more than one ethnicity, and one participant chose "other" as their ethnicity.

Measures and Materials

As in Study 1, the independent variable in Study 2 consisted of scores on the ESJ scale (α = 0.78) administered as part of the pretest survey during the first session.

The pretest survey also included demographic questions including participants' gender, age, education level, ethnicity, political orientation, party affiliation, religiosity, country of birth, years lived in the U.S., and household income. For exploratory purposes, participants also completed measures of subjective socioeconomic status, subjective social class, general system justification, and Belief in a Just World. In addition, participant responded to the Empathic Concern (EC) sub-scale of Interpersonal Reactivity Index (IRI).[17] Sample item included, "I often have tender, concerned feelings for people less fortunate than me" and "Other people's misfortunes do not usually disturb me a great deal" (reverse-coded). Participants indicated how well each item described them on a scale from 1 (Does not describe me) to 5 (Describes me very well).

For the dependent measures, administered during the second session, participants reported how much sadness, pity, and empathy they felt toward the person in the video and how much anger, sadness, disgust they felt toward the American socioeconomic system. Emotions were rated on a scale from 0 to 100. We also asked participants some memory questions for the content of the videos they watched. As in Study 1, we also administered the BIDR at the end of the second session.

The control and homeless videos shown to participants were identical to those used in Study 1. In addition, each participant watched one of two cystic fibrosis (CF) videos involving a CF patient (a White man and a White woman). In these clips, the patient talks about their daily routine and the hardships of living with CF disease. The videos were publicly available on the Internet and were identified by entering the keywords “cystic fibrosis” “interview,” and “video” into the Google search engine. The CF videos averaged 125.5 seconds in length; the shorter CF video was 107 seconds long and the longer CF video was 144 seconds long.

Supplementary Results

Following the preregistered analysis plan, we regressed each of our outcome emotion variables on participants’ ESJ scores (*z*-scored), 2 stimulus vectors (Homeless: 1 = homeless, 0 = control; CF: 1 = CF, 0 = control), and the ESJ × Homeless and ESJ × CF interactions. Within condition, the specific stimuli were effects-coded to account for heterogeneity among the videos. We also adjusted for video order in the analysis. Intercepts varied randomly between participants, and robust standard errors were specified. We observed a significant ESJ × Homeless interaction effect on person-directed sadness, empathy, and pity and on system-directed anger, sadness, and disgust (Supplementary Tables 29–34).

Analyses of simple effects indicated that participants both high and low in ESJ reported higher sadness, pity, and empathy toward the person in the homeless as compared to the control video. However, this effect was larger for low-ESJ participants (sadness: $B = 77.307$, $SE\ B = 1.879$, $z = 41.139$, $p < .001$, 95% CI = [73.624, 80.991]; pity: $B = 66.999$, $SE\ B = 2.26$, $z = 29.644$, $p < .001$, 95% CI = [62.569, 71.428]; empathy: $B = 59.172$, $SE\ B = 2.555$, $z = 23.158$, $p < .001$, 95% CI = [54.164, 64.18]) as compared to high-ESJ participants (sadness: $B = 56.819$, $SE\ B = 2.704$, $z = 21.012$, $p < .001$, 95% CI = [51.519, 62.119]; pity: $B = 54.841$, $SE\ B = 2.732$, $z = 20.077$, $p < .001$, 95% CI = [49.487, 60.194]; empathy: $B = 48.599$, $SE\ B = 2.949$, $z = 16.48$, $p < .001$, 95% CI = [42.819, 54.379]).

Analyses of simple effects also indicated that participants both high and low in ESJ reported higher levels of sadness, anger, and disgust directed toward the American socioeconomic system in response to the homeless as compared to the control video. Again, this effect was considerably larger for low-ESJ participants (anger: $B = 58.03$, $SE\ B = 2.773$, z = 20.93, $p < .001$, 95 % CI = [52.596, 63.464]; sadness: $B = 54.558$, $SE\ B = 4.543$, $z = 12.009$, $p < .001$, 95 % CI = [45.654, 63.463]; disgust: $B = 50.012$, $SE\ B = 5.114$, $z = 9.780$, $p < .001$, 95 % CI = [39.989, 60.035]) as compared to high-ESJ participants (anger: $B = 32.556$, $SE\ B = 2.486$, z = 13.093, $p < .001$, 95 % CI = [27.683, 37.429]; sadness: $B = 35.39$, $SE\ B = 3.418$, $z = 10.355$, $p < .001$, 95 % CI = [28.691, 42.088]; disgust: $B = 23.369$, $SE\ B = 3.546$, $z = 6.59$, $p < .001$, 95 % CI = [16.418, 30.319]).

In the homeless video condition, the simple effect of ESJ on person-directed sadness and empathy were significant (sadness: $B = -7.163$, $SE\ B = 1.323$, $z = -5.414$, $p <$

.001, 95% CI = [-9.756, -4.57]; empathy: $B = -6.227$, $SE\ B = 1.221$, $z = -5.100$, $p < .001$, 95% CI = [-8.620, -3.834]). Surprisingly, in the homeless video condition, the simple effect of ESJ on pity was not significant ($B = -2.338$, $SE\ B = 1.532$, $z = -1.526$, $p = .127$, 95% CI = [-5.341, 0.664]).

Furthermore, in the homeless condition, the simple effects of ESJ on system-directed anger, sadness, and disgust were significant (anger: $B = -14.398$, $SE\ B = 1.544$, $z = -9.325$, $p < .001$, 95% CI = [-17.424, -11.372]; sadness: $B = -12.175$, $SE\ B = 1.521$, $z = -8.006$, $p < .001$, 95% CI = [-15.156, -9.195]; disgust: $B = -15.225$, $SE\ B = 1.53$, $z = -9.953$, $p < .001$, 95% CI = [-18.224, -12.227]).

Contrary to our preregistered hypothesis, we observed a significant, although smaller, ESJ × CF Video interaction effect on person-directed sadness as well as system-directed anger, sadness, and disgust. Notably, the effect of ESJ × Video Type was significantly larger (with some effects twice as big) for the ESJ × Homeless interaction contrast as compared to the ESJ × CF condition on person-directed sadness and system-directed anger, sadness, and disgust (see Supplementary Tables 29–34).

For both high-ESJ and low-ESJ participants, the simple effect of the CF video (vs. control) on person-directed sadness was significant (low-ESJ: $B = 74.493$, $SE\ B = 1.921$, $z = 38.771$, $p < .001$, 95% CI = [70.728, 78.259]; high-ESJ: $B = 65.282$, $SE\ B = 2.825$, $z = 23.108$, $p < .001$, 95% CI = [59.745, 70.819]).

For both high-ESJ and low-ESJ participants the simple effect of the CF video (vs. control) on system-directed anger (low-ESJ: $B = 29.426$, $SE\ B = 2.51$, $z = 11.725$, $p < .001$, 95% CI = [24.508, 34.345]; high-ESJ: ($B = 17.229$, $SE\ B = 2.305$, $z = 7.475$, $p < .001$, 95 % CI = [12.711, 21.746]), sadness (low-ESJ: $B = 37.562$, $SE\ B = 2.558$, $z = 14.686$, $p < .001$, 95 % CI = [32.549, 42.575]; high-ESJ: $B = 27.204$, $SE\ B = 2.562$, $z = 10.618$, $p < .001$, 95 % CI = [22.182, 32.225]), and disgust (low-ESJ: $B = 28.744$, $SE\ B = 2.48$, $z = 11.593$, $p < .001$, 95% CI = [23.884, 33.604]; high-ESJ: $B = 15.955$, $SE\ B = 2.079$, $z = 7.676$, $p < .001$, 95% CI = [11.881, 20.029]) was significant.

In the CF video condition, the simple effect of ESJ on person-directed sadness was not significant, $B = -1.524$, $SE\ B = 1.305$, $z = -1.167$, $p = .243$, 95% CI = [-4.083, 1.035]. In the CF condition, the simple effect of ESJ on system-directed anger, sadness, and disgust was significant (anger: $B = -7.76$, $SE\ B = 1.76$, $z = -4.41$, $p < .001$, 95% CI = [-11.208, -4.311]; sadness: $B = -7.386$, $SE\ B = 1.843$, $z = -4.008$, $p < .001$, 95% CI = [-10.998, -3.775]; disgust: $B = -8.426$, $SE\ B = 1.759$, $z = -4.791$, $p = .001$, 95% CI = [-11.873, -4.979]).

As indicated in the main text, to ensure that our findings reflect economic system justification rather than liberal–conservative differences in dispositional empathy, we adjusted for empathic concern by including it and its interactions with the video contrasts in a series of regression models. These models were not preregistered but were tested in order to rule out differences in dispositional empathy as an alternative hypothesis. Results from these models are summarized in the main text in Tables 2 and 3. In these models,

with the inclusion of empathic concern, the ESJ × Homeless interactions remained significant for person-directed sadness and system-directed anger, sadness, and disgust, and marginally significant for person-directed pity (Table 2). In contrast, with the inclusion of empathic concern, only the ESJ × CF interactions for system-directed anger and disgust remained significant (see main text Table 3).

Adjusting for EC[†], analyses of simple effects indicated that participants both high and low in ESJ reported greater sadness toward the person in the homeless (vs. control) video. However, this effect was again larger for low-ESJ participants (B = 74.153, $SE\ B$ = 1.909, z = 38.853, $p < .001$, 95 % CI = [70.412, 77.893]) as compared to high-ESJ participants (B = 60.076, $SE\ B$ = 2.614, z = 22.981, $p < .001$, 95 % CI = [54.952, 65.199]).

Adjusting for EC, analyses of simple effects also indicated that participants high and low in ESJ reported greater levels of sadness, anger, and disgust directed at the American socioeconomic system in response to the homeless (vs. control) video. Again, these effects were considerably larger for low-ESJ participants (anger: B = 56.786, $SE\ B$ = 2.87, z = 19.785, $p < .001$, 95 % CI = [51.161, 62.412]; sadness: B = 56.455, $SE\ B$ = 2.899, z = 19.474, $p < .001$, 95 % CI = [50.773, 62.137]; disgust: B = 57.459, $SE\ B$ = 2.913, z = 19.726, $p < .001$, 95 % CI = [51.749, 63.168]) than for high-ESJ participants (anger: B = 34.106, $SE\ B$ = 2.605, z = 13.094, $p < .001$, 95 % CI = [29.001, 39.211]; sadness: B = 41.117, $SE\ B$ = 2.737, z = 15.023, $p < .001$, 95 % CI = [35.753, 46.482]; disgust: B = 33.907, $SE\ B$ = 2.516, z = 13.477, $p < .001$, 95 % CI = [28.976, 38.838]).

Adjusting for EC, simple effects analyses revealed that the magnitude of the effect of ESJ on person-directed sadness as well as system-directed anger, sadness, and disgust responses to the homeless video were greater than those in response to the CF video (person sadness: B = -6.403, $SE\ B$ = 1.346, z = -4.76, $p < .001$, 95 % CI = [-9.041, -3.766]; system anger: B = -6.751, $SE\ B$ = 1.947, z = -3.47, p = .001, 95 % CI = [-10.568, -2.934]; system sadness: B = -4.639, $SE\ B$ = 2.091, z = -2.22, p = 0.027, 95 % CI = [-8.737, -0.540]; system disgust: B = -6.517, $SE\ B$ = 1. 789, $p < .001$, 95 % CI = [-10.017, -3.017]).

Adjusting for EC, the simple effects of ESJ on person-directed sadness and system-directed anger, sadness, and disgust were significant in the homeless condition (person sadness: B = -4.551, $SE\ B$ = 1.297, z = -3.509, $p < .001$, 95 % CI = [-7.092, -2.009]; system anger: B = -13.135, $SE\ B$ = 1.646, z = -7.978, $p < .001$, 95 % CI = [-16.362, -9.908]; system sadness: B = -10.205, $SE\ B$ = 1.672, z = -6.104, $p < .001$, 95% CI = [-13.482, -6.928]; system disgust: B = -14.014, $SE\ B$ = 1.641, z = -8.542, $p < .001$, 95 % CI = [-17.23, -10.798]).

[†] Here, we only report the results of simple-slope analyses for interactions that remained significant after adjusting for EC.

Study 3

Procedure

New York University undergraduates (NYU) born or raised in the U.S. were recruited to attend laboratory sessions in exchange for course credit. After obtaining consent and explaining the procedure, the experimenter gently abraded sites corresponding to the *corrugator supercilii* and *levator labii superioris* muscles and attached electromyography (EMG) electrodes.[18] Before initiating the experimental sessions, impedance levels for both muscle sites were ensured to be no more than 10 kΩ.[18] Electrodermal activity (EDA) electrodes were placed on the distal phalanges of the participant's non-dominant hand.[19] As in Studies 1 and 2, the ESJ scale was used to measure system-justifying beliefs with respect to the economy.

At the beginning of the experimental session, instructions appeared on the computer monitor directing participants to attend carefully to the stimuli. Participants then watched a control video followed by a homeless video (identical to those used in the previous experiments), presented using E-prime stimulus presentation software,[20] while listening to audio over a pair of headphones. A blank screen lasting for 4 seconds preceded each video clip. Facial muscle activity and skin conductance measurements, collected using a BIOPAC MP150 Data Acquisition System and AcqKnowledge 4.3 software,[21] were recorded while the participants watched the videos and for 1 second prior to their onset. Participants also continuously reported their level of positive affect by sliding a finger left or right on a touchpad. An experimenter monitored the experimental sessions on a closed-circuit camera. After the conclusion of the second video, electrodes were removed, and participants completed a questionnaire containing demographic items and exploratory measures. Participants were then debriefed, thanked, and dismissed.

Participants

The target sample size was determined by the number of participants who could be run before the end of the academic term. Of the 54 participants originally recruited, 12 were excluded from analysis—3 due to audio and/or video malfunctions, 3 because they were not born or raised in the United States, 2 because of noncompliance with the experimenter's instructions, 2 because their pre-test data could not be located, 1 because impedance levels of her facial EMG electrodes could not be kept below 10 kΩ (34), and 1 who had an extreme emotional reaction during the session. Thus, the final sample consisted of 42 participants (18 males, 24 females), aged 18 to 31 years (M = 19.88, SD = 2.63). Sixteen participants identified as White, 12 as Asian American, 6 as Latinx, 2 as Black or African American, with 6 participants choosing "other" as their race/ethnicity. Of these 42, one participant's touchpad data was omitted from analysis because she reported in debriefing that she forgot to move her finger while the videos played. Additionally, one participant's *levator* data and one participant's EDA data were excluded from analysis because electrodes fell off during the session.

Measures and Materials

Facial EMG signals were obtained using 2.5 cm^2 disposable solid gel Ag/AgCl

electrode pairs at the locations of the *corrugator supercilii* and *levator labii superioris* muscles.[18] Signals were digitized at a rate of 2,000 Hz with a high-pass 10 Hz filter and a low-pass 500 Hz filter using the BIOPAC EMG100C module. Signals were smoothed using the AcqKnowledge Root Mean Square (RMS) automatic EMG analysis routine with a 10-ms time constant. Mean RMS *corrugator* and *levator* muscle activity (in microvolts) were calculated for a 1-second period preceding the beginning of each video and for every second during stimulus presentation. The RMS muscle activity for each 1-second period during the videos was adjusted by subtracting the baseline RMS activity from it. Thus, positive and negative values indicate muscle activity above and below the pre-stimulus baseline, respectively.

Skin conductance level (SCL) was measured using direct current exosomatic recording. Constant current of 0.5 V was applied using a BIOPAC EDA100C amplifier to a pair of disposable 11 mm Ag/AgCl electrodes placed on the index and middle fingers of the participants' non-dominant hand. The signal was sampled at a rate of 2,000 Hz with 1 Hz low-pass filter. The per-second mean SCL (in microsiemens) was computed using BIOPAC's AcqKnowledge 4.3 software. SCL difference scores were calculated by subtracting mean SCL during stimulus presentation from the mean SCL in the second preceding stimulus presentation. Positive and negative values, therefore, indicate skin conductance levels above and below the pre-stimulus baseline, respectively.

Participants provided self-report ratings of affective valence by moving the index finger of their dominant hand left or right on a touchpad to indicate how negatively or positively they felt as they watched the videos. The left-most side of the touchpad was labeled 0 ("extremely negative and unpleasant") and the right-most side of the touchpad was labeled 100 ("extremely positive and pleasant"). By default, the initial location of the finger on the touchpad was recorded at 50 ("neither positive nor negative"). During stimulus presentation, E-prime software recorded the participants' finger position on the touchpad every .5 seconds.

Prior to processing the physiological signals, a research assistant blind to participants' ESJ scores inspected all participant videos for behavioral anomalies. Signal artifacts due to gross motor movements such as yawning, sneezing, coughing, and touching the electrodes or face were flagged[22, 23] and replaced with interpolated data using the AcqKnowledge software Connect Endpoints transformation routine.

The homeless and control videos shown to participants were the same as those used in Study 1. In Study 3, participants always saw the homeless video after the control video. To ensure that no single video disproportionately affected the results, we only analyzed psychophysiological data corresponding to the first 126 seconds of the videos (i.e., the length of the shortest homeless clip).

At the end of the lab sessions, participants provided information about their age, gender, ethnicity, political orientation, religiosity, parents' education, and parents' income. Several additional measures were administered for exploratory purposes. These measures included social class identification,[4] psychological entitlement,[24] sense of

control, attitudes toward greed,[25] social dominance orientation (SDO),[26] incremental vs. entity theories of social class, dispositional compassion,[18] dispositional vs. contextual explanation of social class, social class essentialism,[28] social mobility beliefs, personal sense of power,[29] right-wing authoritarianism,[30] general system justification,[5] wealth-related guilt, communal orientation,[31] and Protestant Work Ethic.[6]

As in Studies 1 and 2, our primary predictor was the 17-item ESJ scale, which was administered several weeks prior to the laboratory sessions as part of the NYU Psychology Department battery questionnaire.

Supplementary Results

In a series of mixed-effects linear regression, we regressed each of our outcome variables on participants' ESJ scores (*z*-scored), stimulus condition (1 = homeless, 0 = control), and the ESJ × Homeless interaction. Within condition, specific stimuli were effect-coded to account for heterogeneity among the videos. Random intercepts and random slopes for video condition, unstructured random effects variance covariance matrix, and robust standard errors were specified.

Consistent with our hypothesis, we observed a significant interactive effect of ESJ and video condition on *corrugator* activity and SCL (Supplementary Tables 43 and 45). Analyses of simple effects confirmed that, while participants who scored relatively low on the ESJ scale (1 SD below the mean) displayed an increase in *corrugator* activation in response to the homeless video ($B = 2.875$, $SE\ B = 1.252$, $z = 2.295$, $p = .022$, 95% CI = [0.42, 5.33]), those who scored high on the ESJ scale (1 SD above the mean) did not ($B = -0.139$, $SE\ B = 0.645$, $z = -0.215$, $p = .829$, 95% CI = [-1.40, 1.13]).

In the homeless video condition, the simple effect of ESJ on *corrugator* activity was significant, $B = -1.04$, $SE\ B = 0.436$, $z = -2.387$, $p = .017$, 95% CI = [-1.894, -0.186].

Similarly, whereas the homeless video significantly increased SCL among low-ESJ participants ($B = 0.966$, $SE\ B = 0.36$, $z = 2.682$, $p = .007$, 95% CI = [0.26, 1.67]), the homeless video had no significant effect on SCL among high-ESJ participants ($B = -0.169$, $SE\ B = 0.28$, $z = -0.601$, $p = .548$, 95% CI = [-0.72, 0.38]).

In the homeless video condition, the simple effect of ESJ on SCL was significant, $B = -0.443$, $SE\ B = 0.16$, $z = -2.771$, $p = .006$, 95% CI = [-0.756, -0.129].

Consistent with the hypothesis, we also observed a marginally significant ESJ × Homeless interaction on self-reported affect (Supplementary Table 46).

Levator activity (associated with retraction of the nostrils) is related to physical and moral disgust.[32, 33] Our expectations with regard to *levator* activation in Study 2 were uncertain. On one hand, low-ESJ individuals may feel morally disgusted at the economic system when faced with exemplars of economic inequality, and thus exhibit greater *levator* activity than high-ESJ individuals. For different reasons, however, high-ESJ individuals might show greater *levator* activity in response to the homeless. For instance,

research suggests that conservatives are higher in disgust sensitivity than liberals.[34] Therefore, we might expect that high-ESJ would feel physically disgusted by homelessness. Despite these equivocal expectations, we predicted that low-ESJ individuals would show higher *levator* activity due to their greater affective engagement with the homeless videos. Consistent with this prediction, we observed a trending ESJ × Homeless interaction on the *levator* activity (Supplementary Table 44).

We also SDO and ran regression models predicting physiological responses on the basis of SDO, Video Condition (homeless vs. control), and their interactions. None of the coefficients for the interactive effect of SDO and video condition were significantly different from zero in these models.

Study 4

Procedure

New York University undergraduates (NYU) born or raised in the U.S. were recruited to attend laboratory sessions in exchange for course credit. The procedure matched that of Study 3, with three exceptions: in Study 4, an equipment malfunction prevented us from collecting EDA responses, baseline measurement periods were extended to 2 minutes, and we used reusable (rather than disposable) electrodes to record EMG signals.

The desired sample size, rules for termination of data collection, hypotheses, data-exclusion criteria, and detailed description of the experimental procedure were preregistered on the Open Science Framework (OSF) (see https://osf.io/2qn2z/registrations).

Participants

Of the 39 participants originally recruited, 2 participants' *corrugator* and *levator* muscle activity data were excluded from analysis because of high impedance (>10 kΩ). Thus, the final sample consisted of 37 participants (13 males, 24 females) ranging in age from 18 to 40 (M = 19.81, SD = 3.72). Seventeen participants identified as White, 9 as Asian American, 5 as Black or African American, 4 as Latinx, and 2 chose "other" as their race/ethnicity.

Measures and Materials

Reusable Ag/AgCl electrode pairs with a recording diameter of 4 mm were used to collect facial EMG data. Facial EMG data were preprocessed using the same methods employed in Study 2.

After undergoing physiological measurement, participants also completed a questionnaire probing their age, gender, ethnicity, political orientation, religiosity, parents' education, and parents' income. As additional exploratory measures, participants reported their social class identification[4] and subjective well-being,[35] as well as the amount of blame, responsibility, and controllability they attributed to the homeless

individual in the videos and their situation.

As in previous experiments, our primary predictor variable was ESJ, which was measured several weeks prior to the laboratory sessions.

Supplementary Results

In a series of mixed-effects linear regressions, we regressed each of our outcome variables on participants' ESJ scores (*z*-scored), stimulus condition (1 = homeless, 0 = non-homeless), and the ESJ × Homeless interaction. Within condition, specific stimuli were effect-coded to account for heterogeneity among the videos. Random intercepts and random slopes for video condition, unstructured random effects variance covariance matrix, and robust standard errors were specified.

Consistent with the hypothesis, and replicating Study 3, we observed a significant ESJ × Homeless interaction on *corrugator* activity (Supplementary Table 49). Analyses of simple effects confirmed that low-ESJ participants showed a significant increase in *corrugator* activity in response to the homeless (vs. control) video (B = 5.63, $SE\ B$ = 1.101, z = 5.115, p < .001, 95% CI [3.473, 7.79]). In contrast, high-ESJ participants only showed a marginally significant increase in *corrugator* activity in response to the homeless video (B = 2.16, $SE\ B$ = 1.16, z = 1.862, p = .063, 95% CI [-0.113, 4.433]). In the homeless video condition, the simple effect of ESJ on *corrugator* activity was not significant (B = -1.047, $SE\ B$ = 0.705, z = -1.484, p = .138, 95 % CI = [-2.43, 0.336].

Contrary to our hypothesis and the findings of Study 2, we did not observe an interactive effect of ESJ and video condition on *levator* activity or self-reported positive affect (Supplementary Tables 50 and 51).

Study 5

Procedure

NYU undergraduates born or raised in the U.S. were recruited to attend laboratory sessions in exchange for course credit. The procedure and data processing methods were similar to Studies 3 and 4, with one exception: in this experiment, we added the CF video condition involving patients suffering from disease. The CF videos were previously used in Study 2.

The desired sample size, rules for termination of data collection, hypotheses, data-exclusion criteria, and detailed description of the experimental procedure were preregistered on the Open Science Framework (OSF) (see https://osf.io/2qn2z/registrations).

Participants

Of the 80 recruited participants, 4 participants' data were excluded from the analyses due to noncompliance with the experimenter's instructions. Thus, the final sample included 76 participants (19 males, 57 females) ranging in age from 17 to 24 (*M* =

19.14, *SD* = 1.16). Thirty-seven participants identified as White, 23 as Asian American, 10 as Latinx, 4 as Black or African American, and 2 participants indicated "other" as their race/ethnicity. Seven participants' *levator* data were excluded because of a broken electrode. One participant's *levator* data were discarded because electrodes fell off during the session and one participant's *levator* data were excluded because of high impedance (>10 kΩ). One participant's EDA data were lost due to experimenter error and 1 participant's EDA could not be properly recorded.

Measures and Materials

The homeless and control videos were the same as those used in the previous studies. In addition, each participant viewed a CF video previously used in Study 2. We again only analyzed psychophysiological data corresponding to the first 126 seconds of the videos (i.e., the length of the shortest homeless clip).

As in Study 3, the baseline periods between videos lasted 2 minutes, and facial EMG signals were obtained using 4 mm reusable solid gel Ag/AgCl electrode pairs. The facial EMG and EDA signals were recorded and preprocessed using the methods described in Studies 3 and 4.

At the end of the lab sessions, participants provided information about their age, gender, ethnicity, political orientation, religiosity, parents' education and income, social class identification[4], and subjective well-being[35] as well as the amount of blame, responsibility, and controllability they attributed to the homeless person.

As in previous studies, our primary predictor variable was ESJ, which was measured several weeks prior to the laboratory sessions.

Supplementary Results

In a series of mixed-effects linear regressions, we regressed each of our outcome variables on participants' ESJ scores (*z*-scored), 2 stimulus vectors (homeless: 1 = homeless, 0 = control; CF: 1 = CF, 0 = control), and the ESJ × Homeless and ESJ × CF interactions. As in previous experiments, within each condition, the specific stimuli were effects-coded to account for heterogeneity among the videos. We additionally adjusted for stimulus order in the model. Random intercepts and random slopes for video condition, unstructured covariance matrices, and robust standard errors were specified.

Consistent with our hypothesis, we observed a marginally significant ESJ × Homeless interaction effect on *corrugator* activity and a significant ESJ × Homeless interaction on SCL (Supplementary Tables 55 and 57). Analyses of simple effects indicated that participants both high and low in ESJ displayed an increase in corrugator activation in response to the homeless video (low-ESJ: $B = 4.012$, $SE\ B = 0.766$, $z = 5.241$, $p < .001$, 95% CI = [2.512, 5.513]; high-ESJ: $B = 2.628$, $SE\ B = 0.761$, $z = 3.453$, $p = .001$, 95% CI = [1.136, 4.119]). In the homeless video condition, the simple effect of ESJ on *corrugator* activity was not significant ($B = -0.825$, $SE\ B = 0.571$, z = -1.444, $p = .149$, 95 % CI = [-1.945, 0.295].

Whereas the homeless video significantly increased SCL among low-ESJ participants (B = 0.879, $SE\ B$ = 0.232, z = 3.793, $p < .001$, 95% CI = [0.425, 1.332]), it had no significant effect on high-ESJ participants (B = 0.007, $SE\ B$ = 0.217, z = 0.034, p = .973, 95% CI = [-.417, .432]). In the homeless video condition, the simple effect of ESJ on SCL was marginally significant (B = -0.227, $SE\ B$ = .117, z = -1.946, p = .052, 95 % CI = [-0.456, 0.002].

Contrary to our hypothesis and the results found in Study 3, we did not observe a significant interactive effect of ESJ and video condition on levator activity or self-reported positive affect (Supplementary Tables 56 and 58). Finally, as predicted, we did not find a significant CF × ESJ interaction effect on any of our outcome variables (Supplementary Tables 55–58).

Integrative Data Analysis (IDA; Studies 3–5)

Supplementary Results

Analyses of simple slopes indicated that for both low- and high-ESJ participants, the simple effect of exposure to the homeless video on *corrugator* activity was significant. Importantly, this effect was larger for low-ESJ (B = 3.922, $SE\ B$ = 0.573, z = 6.839, $p < .001$, 95% CI = [2.798, 5.046]) than for high-ESJ participants (B = 1.84, $SE\ B$ = .467, z = 3.944, $p < .001$, 95 % CI = [0.926, 2.755]).

Simple slopes analysis also revealed that for low-ESJ participants, the simple effect of the homeless video on SCL was significant (B = 0.85, $SE\ B$ = 0.174, z = 4.897, $p < .001$, 95 % CI LB = .51, CI UB = 1.19). This effect was not significant for high-ESJ participants (B = -0.02, $SE\ B$ = .168, z = -.119, p = .905, 95% CI = [-0.349, 0.309]).

In the homeless video condition, the simple effect of ESJ on *corrugator* activity and SCL was significant (*corrugator:* B = -0.889, $SE\ B$ = 0.341, z = -2.604, p = .009, 95% CI = [-1.557, -0.22]; SCL: B = -0.281, $SE\ B$ = 0.082, z = -3.446, p = .001, 95% CI = [-0.441, -0.121).

Studies 3–5

Supplementary Discussion

Activation of the *levator labii superioris* muscle was assessed for exploratory purposes in Study 3. Because we observed a marginally significant effect of ESJ by video condition on levator in Study 3, we preregistered it as one of our hypotheses in Studies 4 and 5. In these two preregistered replications, however, our prediction was not supported.

Two factors may explain the lack of consistent effects for self-reported affect in the physiological experiments. First, we measured self-reported affective valence rather than discrete emotions, as in Studies 1 and 2; the latter approach would have provided a more granular and potentially more precise index of emotional experience. Second, participants were required to report their affective valence while simultaneously watching

the videos, whereas participants in Study 1 reported their discrete emotions after watching each video. Thus, participants in the physiological experiments may have had difficulty continuously updating their self-reported responses while engaging with the video.

Across the three physiological studies, we found inconsistent results with respect to the simple slope of ESJ on *Corrugator* and SCL in the homeless condition. In Study 3, the simple slope of ESJ on both *Corrugator* and SCL was significant in the homeless condition. However, in Studies 4 and 5, the simple slope of ESJ on *Corrugator* was not significant in the homeless condition. In Study 5, the simple slope of ESJ on SCL was marginally significant in this condition.

As pointed out in the main text, previous work suggests that there are baseline differences between high and low system-justifiers when it comes to experiencing some positive (e.g., empathy) and negative (e.g., disgust) emotions. Additionally, with regard to physiological indices, methodological experts recommend that, because of major individual and baseline variations in physiological responses,[22, 23] conclusions be drawn on within-participant rather than between-participant comparisons. Therefore, we conclude that inspecting only the effect of ideology on emotion in one condition without comparing it to that of another condition (in within-participants fashion) may be problematic.

It is noteworthy, in any case, that in the combined data analysis from all physiological studies (IDA), in which we have the most power to detect an effect, we do observe significant ESJ–Corrugator and ESJ–SCL effects in the homeless condition. There may be two related reasons why we observed inconsistent findings with respect to simple slopes analyses in the homeless condition across the physiological studies. First, inconsistency may be attributable to baseline individual differences, rendering the ESJ–physiology link in the homeless condition unreliable. Secondly, it is possible that the sample sizes in each individual study (Studies 3–5) were too small to overcome baseline variations and detect ESJ–physiology effects when inspecting results in the homeless condition alone.

Study 6

Procedure

Participants received four text messages a day for nine consecutive days. Three of the surveys were sent during the day and each asked participants about a distinct event. One survey asked participants to indicate whether they had an encounter with someone they considered very poor compared to themselves. Another asked whether they had an encounter with someone they consider much richer than themselves. Another asked whether they have had an argument with someone. Each survey focused on one of these categories, with the order counterbalanced across days. For each category, participants who indicated they had such an encounter were asked to describe that encounter, and subsequently reported their emotions in response to the encounter and how they regulated them. Participants who reported no encounter responded to the same set of emotion

items, but addressing how they generally felt in the preceding couple of hours. Participants also received a recap nightly survey, which asked them to reflect back on each of these events (if any had occurred) and emotions they may have felt during these events.

Participants

Seventy-one NYU undergraduate participants were recruited for this study (24 males, 47 females) ranging in age from 17.5 to 22.4 ($M = 19.24$, $SD = 1.12$). Thirty-seven participants identified as White, 15 as Asian American, 12 as Latinx, 3 as Black or African American, and 3 indicated "more than one race," with 1 participant indicating "other" as their race/ethnicity.

Measures and Materials

The study was designed to answer research questions from two separate research projects, and therefore included measures that we do not address in the present paper. Nonetheless, all measures are detailed below.

Before daily data collection began, all participants completed an intake survey in the lab. This initial questionnaire included an informed consent letter and several background measures. In the intake survey, each participant was asked to indicate their university email address, the zip code where they currently live, and their mode of commuting to NYU. If they indicated having a job outside the university, the zip code of that job and the mode of commuting to it were also asked. Participants also provided their phone numbers and their carrier, to allow us to send them text messages with links to the daily survey. Participants also responded to measures of subjective happiness[35] and life satisfaction.[36] These questions were followed by a series of items on emotion regulation tendencies, focused on the motivation to regulate anger, empathy, and sadness toward a close other and in response to inequality (six items) as well as self-reported general success and difficulty in regulating emotions (one item for each). Participants also indicated the extent to which they generally felt different sentiments toward the rich, poor, and inequality in general (e.g., "contempt for the poor").

Three daily surveys, filled out on each of the nine days, focused on participants' specific experiences in the couple of hours preceding the reception of that survey. At the beginning of each survey, participants were instructed to "Please think about the last couple of hours…" The instructions then diverged for each survey. For the "poor" survey, participants were asked whether in the past couple of hours, they "encountered or heard about a person/people in the U.S. who is/are much poorer than" them. In the "rich" survey the word "poorer" was replaced with "richer." Finally, in the "interpersonal" survey, they were asked whether they "had an argument or conflict with someone?" Participants who answered "yes" to any of these were then provided a text box to describe "this person/people and the situation in which you encountered or heard about

them" ("poor" and "rich" surveys) ‡ or the "argument or conflict, the person involved, and the situation in which it occurred" ("interpersonal" survey)

Next, all participants were asked about their emotions, either following the encounter/argument or, if they indicated no such event, about the past couple of hours preceding the receipt of the survey. These emotions were as follows: "Empathy toward the poor person/people," "Anger toward the poor person/people," "Sadness about the poor person/people's situation," "Anger toward the American economic system," and "Disgusted by the American economic system" ("poor" survey); "Empathy toward the rich person/people," "Anger toward the rich person/people," "Jealousy of the rich person/people," "Sadness about your status," "Anger toward the American economic system," and "Disgusted by the American economic system" ("rich" survey); "Empathy toward the person involved in the argument," "Anger toward the person involved in the argument," "Sadness about the situation," and "Disgusted by the person involved in the argument" ("interpersonal" survey). Again, we followed a discrete emotion approach for the reasons specified above (Study 1 Supplementary Discussion). In addition to the discrete emotions measured in Study 1, we also measured jealousy in this study. This is because our study investigated reactions to advantaged (as well as disadvantaged) others, and upward social comparison tends to arouse envy and/or jealousy.[10] All emotions were rated on a 1 (not at all) to 7 (extremely) scale. These were our central dependent variables, and they were followed by items assessing emotion regulation, namely the desired direction of regulation of several emotions toward the target (all surveys) and the system ("poor" and "rich" surveys), and the strategies employed for regulation (focusing on reappraisal and attention deployment, see[37]). Finally, each of the three surveys gauged momentary life satisfaction by asking people to what extent they felt "satisfied" with their lives, "happy," "accomplished," and "fortunate" in the preceding couple of hours. These three surveys were sent out in different order each day across the period of administration, with one sent in the morning, one around midday, and one in the late afternoon.

Every evening, participants received a link to the fourth survey, which involved reflecting on the day. Participants were asked to think of their day and report how many times they encountered or heard of someone "much poorer" than them, how many times they encountered or heard of someone "much richer" than them, and how many times they had "and argument or conflict with someone." After each of these questions, participants who reported having one or more of the three experiences responded to questions on their emotions, emotion regulation, and life satisfaction similar to the shorter daily surveys.

Happiness and life satisfaction items as well as emotion regulation questions are collected as part of a separate project and were not analyzed for present purposes.

Supplementary Results

‡ After the completion of data collection, two research assistants categorized the type of rich and poor encounters based on the description stated in the textbox (see Study 6 Supplementary results).

Overall, participants reported having an encounter with (or hearing about) a poor target 30% of the time and having an encounter with (or hearing about) a rich target 30% of the time across the assessment period.

Of the 172 total poor encounters reported, 61.40% involved direct exposure to homelessness on the streets. 18.13% were direct encounters with someone the participant personally knew (e.g., family member, roommate, acquaintance) and 5.26% were direct encounters with a (non-homeless) stranger. 6.40% of the responses involved indirect exposure through media and Internet, and 2.30% involved indirect exposure through discussion with others. 5.85% of the responses were categorized by the raters as something other than the categories described. One observation was excluded from analysis because the participant mistakenly reported about a different target category than poor others.

Of the 177 total rich encounters, 46.33% involved direct encounter with someone they personally knew. 37.85% involved indirect exposure through media and Internet. 8.47% involved discussions with others. 4.52% of the responses were categorized by the raters as something other than the categories described above. 2.82% (five instances) of the encounters were characterized as erroneous by the raters. These observations were excluded from further analysis as the participant mistakenly reported about a different target category than rich others.[§]

We first calculated a person-level mean of the poor encounter variable, by averaging rich and poor encounter responses across the nine days for each participant. This would allow us to isolate between- (i.e. time invariant) as well as within-subject (time-varying) differences in rich and poor encounters.

For each of the rich and poor response categories, mixed effect linear regression models were conducted to estimate the between- and within-subject interactive effects of inequality (rich or poor) encounter by ESJ on each of the emotion reports. The intercept and the slope of inequality encounter were allowed to vary randomly between participants. We requested Restricted Maximum Likelihood (REML) estimators and unstructured variance covariance matrix for random effects for these models.

As predicted, we found a significant negative two-way interaction effect of within-subject poor encounter by ESJ on anger and disgust at the system (see Supplementary Tables 67 and 68). Analysis of simple effects revealed that although all participants reported increased disgust and anger at the system when they encountered poor targets, these effects were larger for low-ESJ (anger: $B = 1.453$, $SE\ B = 0.211$, $z = 6.883$, $p < .001$, 95 % CI = [1.039, 1.867]; disgust: $B = 1.55$, $SE\ B = 0.206$, $z = 7.53$, $p < .001$, 95 % CI = [1.147, 1.954]) as compared to high-ESJ (anger: $B = 0.732$, $SE\ B = 0.219$, $z = 3.346$, $p = .001$, 95 % CI = [0.303, 1.16]; disgust: $B = 0.587$, $SE\ B = 0.213$, $z = 2.751$, $p = .006$, 95 % CI = [0.169, 1.005]) participants.

[§] Results from all analyses reported for Study 6 are consistent with or without the exclusion of the erroneous responses. Results in which these 6 responses are excluded from the analyses are reported in this paper.

On days when participants encountered a poor target, the simple effects of ESJ on system-directed anger and disgust were significant (anger: B = -0.807, $SE\ B$ = 0.188, z = -4.286, p < .001, 95 % CI = [-1.177, -0.438]; disgust: B = -0.93, $SE\ B$ = 0.184, z = -5.043, p = .001, 95 % CI = [-1.291, -0.569]).

We found a significant two-way interaction effect of within-subject poor encounter by ESJ on person-directed anger (see Supplementary Table 69), such that low-ESJ (B = 0.42, $SE\ B$ = 0.16, z = 2.69, p = .007, 95 % CI = [0.113, 0.718]) but not high-ESJ (B = -0.188, $SE\ B$ = 0.16, z = -1.176, p = .25, 95 % CI = [-0.502, 0.125]) participants felt anger when they encountered a poor person. On days when participants encountered a poor target, the simple effect of ESJ on person-directed anger was not significant (B = -0.077, $SE\ B$ = 0.116, z = -0.665, p = .506, 95 % CI = [-0.305, 0.151]).

As predicted, we found a negative two-way interaction effect of within-subject rich encounter by ESJ on anger and disgust at the system (see Supplementary Tables 72 and 73 for full regression results). Analysis of simple effects revealed that whereas low-ESJ participants felt anger and disgust at the system when they encountered rich targets (anger: B = 1.037, $SE\ B$ = 0.263, z = 3.945, p < .001, 95 % CI = [0.522, 1.552]; disgust: B = 0.916, $SE\ B$ = 0.272, z = 3.367, p = .001, 95 % CI = [0.383, 1.45]), high-ESJ did not (anger: B = 0.061, $SE\ B$ = 0.264, z = 0.23, p = 0.818, 95 % CI = [-0.458, 0.579]; disgust: B = 0.087, $SE\ B$ = 0.274, z = 0.316, p = .752, 95 % CI = [-0.45, 0.624]).

On days when participants encountered a rich target, the simple effect of ESJ on system-directed anger and disgust was significant (anger: B = -0.745, $SE\ B$ = 0.189, z = -3.936, p < .001, 95 % CI = [-1.115, -0.374]; disgust: B = -0.671, $SE\ B$ = 0.195, z = -3.431, p = .001, 95 % CI = [-1.054, -0.287]).

We also found a significant two-way interaction effect of within-subject rich encounter by ESJ on anger and jealousy felt toward the rich target as well as sadness felt about one's socioeconomic status (see Supplementary Tables 74–76). Analysis of simple effects revealed that whereas low-ESJ participants felt anger, jealousy, and sadness when they encountered rich targets (anger: B = 0.826, $SE\ B$ = 0.25, z = 3.299, p = .001, 95 % CI = [0.335, 1.316]; jealousy: B = 1.076, $SE\ B$ = 0.258, z = 4.171, p < .001, 95 % CI = [0.570, 1.582]; sadness: B = 0.523, $SE\ B$ = 0.209, z = 2.506, p = .012, 95 % CI = [0.114, 0.932]), high-ESJ did not (anger: B = 0.01, $SE\ B$ = 0.253, z = 0.04, p = .968, 95 % CI = [-0.485, 0.505]; jealousy: B = 0.185, $SE\ B$ = 0.26, z = 0.713, p = 0.476, 95 % CI = [-0.324, 0.694]; sadness: B = -0.147, $SE\ B$ = 0.21, z = -0.699, p = .485, 95 % CI = [-0.560, 0.265]).

On days when participants encountered a rich target, the simple effect of ESJ on target-directed anger was significant (B = -0.406, $SE\ B$ = 0.173, z = -2.349, p = .019, 95 % CI = [-0.746, -0.067]). On days when participants encountered a rich target, the simple effect of ESJ on self-directed sadness was marginally significant (B = -0.305, $SE\ B$ = 0.166, z = -1.833, p = .067, 95 % CI = [-0.631, 0.021]). On days when participants encountered a rich target, the simple effect of ESJ on jealousy toward the rich target was not significant (B = -0.259, $SE\ B$ = 0.193, z = -1.345, p = 0.179, 95 % CI = [-0.637, 0.119]).

Supplementary References

1. Jost, J. T. & Thompson, E. P. Group-based dominance and opposition to equality as independent predictors of self-esteem, ethnocentrism, and social policy attitudes among African Americans and European Americans. *J. Exp. Soc. Psychol.* **36**, 209–232 (2000).

2. Paulhus, D. L. & Reid, D. B. Enhancement and denial in socially desirable responding. *J. Pers. Soc. Psychol.* **60**, 307–317 (1991).

3. Adler, N. E., Epel, E. S., Castellazzo, G. & Ickovics, J. R. Relationship of subjective and objective social status with psychological and physiological functioning: Preliminary data in healthy, White women. *Heal. Psychol.* **19**, 586–592 (2000).

4. Jackman, M. R. & Jackman, M. R. *Class Awareness in the United States*. (University of California Press, 1983).

5. Kay, A. C. & Jost, J. T. Complementary justice: Effects of 'poor but happy' and 'poor but honest' stereotype exemplars on system justification and implicit activation of the justice motive. *J. Pers. Soc. Psychol.* **85**, 823–837 (2003).

6. Levy, S. R., West, T. L., Ramirez, L. & Karafantis, D. M. The Protestant work ethic: A lay theory with dual intergroup implications. *Gr. Process. Intergr. Relations.* **9**, 95–115 (2006).

7. Lipkus, I. The construction and preliminary validation of a global belief in a just world scale and the exploratory analysis of the multidimensional belief in a just world scale. *Pers. Individ. Dif.* **12**, 1171–1178 (1991).

8. Lerner, J. S. & Keltner, D. Beyond valence: Toward a model of emotion-specific influences on judgement and choice. *Cogn. Emot.* **14**, 473–493 (2000).

9. Rozin, P., Haidt, J. & McCauley, C. R. Disgust. in *Handbook of Emotions* (eds. Lewis, M., Haviland-Jones, J. M. & Barrett, L. F.) 757–776 (The Guilford Press, 2008)

10. Fiske, S. T., Cuddy, A. J. C., Glick, P. & Xu, J. A model of (often mixed) stereotype content: Competence and warmth respectively follow from perceived status and competition. *J. Pers. Soc. Psychol.* **82**, 878–902 (2002).

11. Batson, C. D., Chang, J., Orr, R. & Rowland, J. Empathy, attitudes, and action: Can feeling for a member of a stigmatized group motivate one to help the group? *Personal. Soc. Psychol. Bull.* **28**, 1656–1666 (2002).

12. Lewis, M. Self-conscious emotions: Embarrassment, pride, shame, and guilt. in *Handbook of Emotions* (eds. Lewis, M., Haviland-Jones, J. M. & Barrett, L. F.) 742–756 (The Guilford Press, 2008).

13. Tangney, J. P. Moral affect: The good, the bad, and the ugly. *J. Pers. Soc. Psychol.* **61**, 598–607 (1991).

14. Wilcox, K., Kramer, T. & Sen, S. Indulgence or Self-Control: A Dual Process Model of the Effect of Incidental Pride on Indulgent Choice. *J. Consum. Res.* **38**, 151–163 (2011).

15. Snyder, C. R. *The psychology of hope: You can get there from here*. (Free Press, 1994).

16. Snyder, C. R. The past and possible futures of hope. *J. Soc. Clin. Psychol.* **19**, 11–28 (2000).

17. Davis, M. H. Measuring individual differences in empathy: Evidence for a multidimensional approach. *J. Pers. Soc. Psychol.* **44**, 113–126 (1983).

18. Fridlund, A. J. & Cacioppo, J. T. Guidelines for human electromyographic research. *Psychophysiology* **23**, 567–589 (1986).

19. Boucsein, W. *et al.* Publication recommendations for electrodermal measurements. *Psychophysiology* **49**, 1017–1034 (2012).

20. Psychology Software Tools Inc. E-Prime 2.0. (2012).

21. BIOPAC Systems. AcqKnowledge 4 software guide.

22. Hess, U. Facial EMG. in *Methods in Social Neuroscience* (eds. Harmon-Jones, E. & Beer, J. S.) 70–91 (Guildford Press, 2009).

23. Boucsein, W. *Electrodermal Activity*. (Springer Science + Business Media, 2012).

24. Campbell, W. K., Bonacci, A. M., Shelton, J., Exline, J. J. & Bushman, B. J. Psychological entitlement: Interpersonal consequences and validation of a self-report measure. *J. Pers. Assess.* **83**, 29–45 (2004).

25. Piff, P. K., Stancato, D. M., Côté, S., Mendoza-Denton, R. & Keltner, D. Higher social class predicts increased unethical behavior. *Proc. Natl. Acad. Sci. USA.* **109**, 4086–4091 (2012).

26. Ho, A. K. *et al.* The nature of social dominance orientation: Theorizing and measuring preferences for intergroup inequality using the new SDO7 scale. *J. Pers. Soc. Psychol.* **109**, 1003–1028 (2015).

27. Stellar, J. E., Manzo, V. M., Kraus, M. W. & Keltner, D. Class and compassion: Socioeconomic factors predict responses to suffering. *Emotion* **12**, 449–459 (2012).

28. Kraus, M. W. & Keltner, D. Social class rank, essentialism, and punitive judgment.

J. Pers. Soc. Psychol. **105**, 247–261 (2013).

29. Anderson, C., John, O. P. & Keltner, D. The personal sense of power. *J. Pers.* **80**, 313–44 (2012).

30. Zakrisson, I. Construction of a short version of the Right-Wing Authoritarianism (RWA) scale. *Pers. Individ. Dif.* **39**, 863–872 (2005).

31. Clark, M. S., Oullette, R., Powell, M. C. & Milberg, S. Recipient's mood, relationship type, and helping. *J. Pers. Soc. Psychol.* **53**, 94–103 (1987).

32. Vrana, S. R. The psychophysiology of disgust: Differentiating negative emotional contexts with facial EMG. *Psychophysiology* **30**, 279–286 (1993).

33. Whitton, A. E., Henry, J. D., Rendell, P. G. & Grisham, J. R. Disgust, but not anger provocation, enhances levator labii superioris activity during exposure to moral transgressions. *Biol. Psychol.* **96**, 48–56 (2014).

34. Inbar, Y., Pizarro, D. A., Bloom, P. & Haven, N. Conservatives are more easily disgusted than liberals. *Cogn. Emot.* **23**, 714–726 (2009).

35. Lyubomirsky, S. & Lepper, H. S. A measure of subjective happiness: Preliminary reliability and construct validation. *Soc. Indic. Res.* **46**, 137–155 (1999).

36. Diener, E., Emmons, R. A., Larsen, R. J. & Griffin, S. The Satisfaction with Life Scale. *J. Pers. Assess.* **49**, 71–75 (1985).

37. Gross, J. J. Emotion regulation: Current status and future prospects. *Psychol. Inq.* **26**, 1–26 (2015).

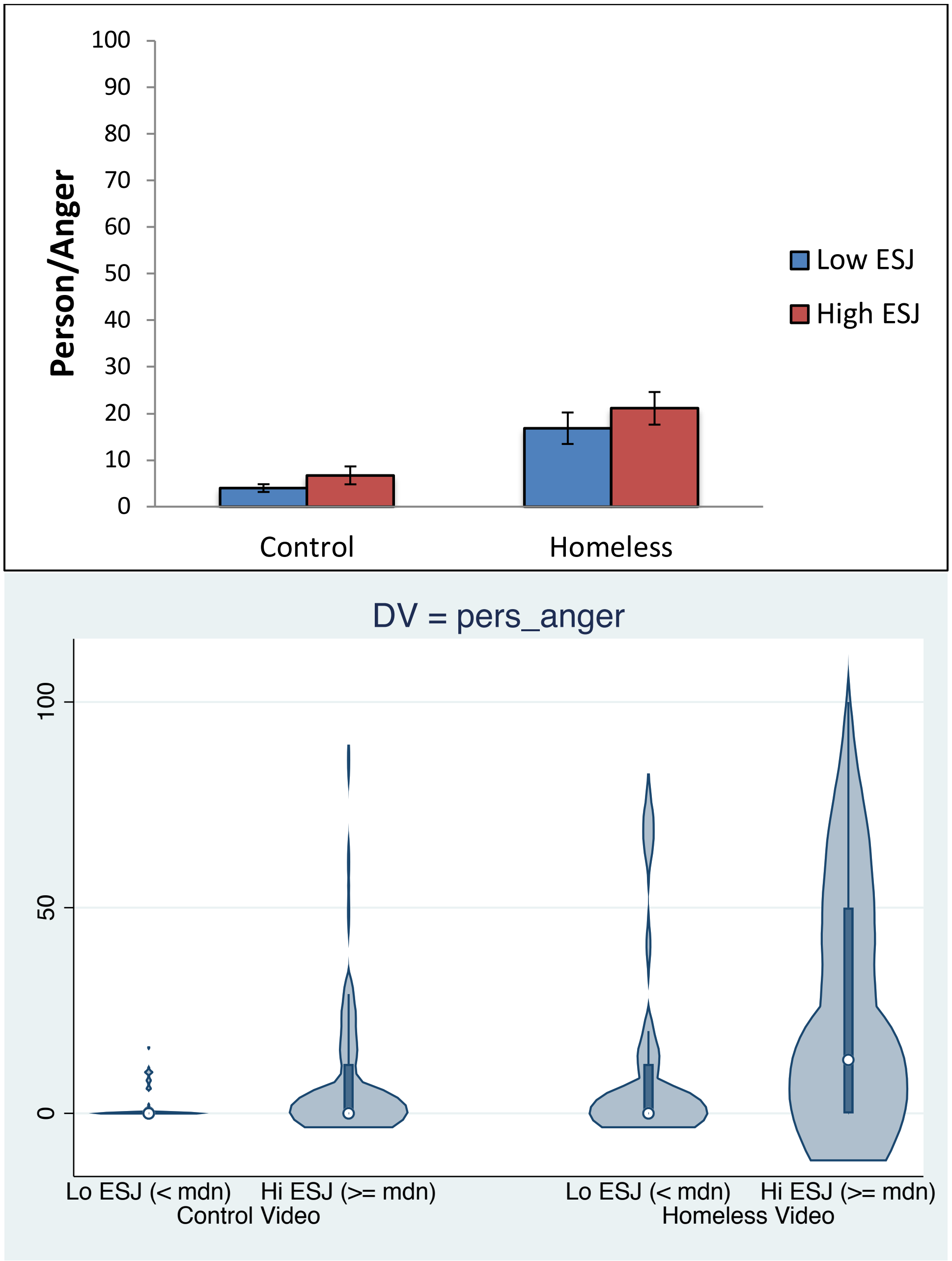

Supplementary Figure 1. *ESJ × Video Type interaction for person-directed anger in Study 1, p = .70. Simple effect of ESJ in control video condition, p = .14; simple effect of ESJ in homeless video condition, p = .36. Simple effect of video type (homeless vs. control) at low (-1 SD) ESJ, p < .001; simple effect of video type (homeless vs. control) at high (+1 SD) ESJ, p < .001. Emotions were rated on a 0–100 scale. Error bars represent standard errors. Bottom graph shows description statistics and distributional properties based on raw data.*

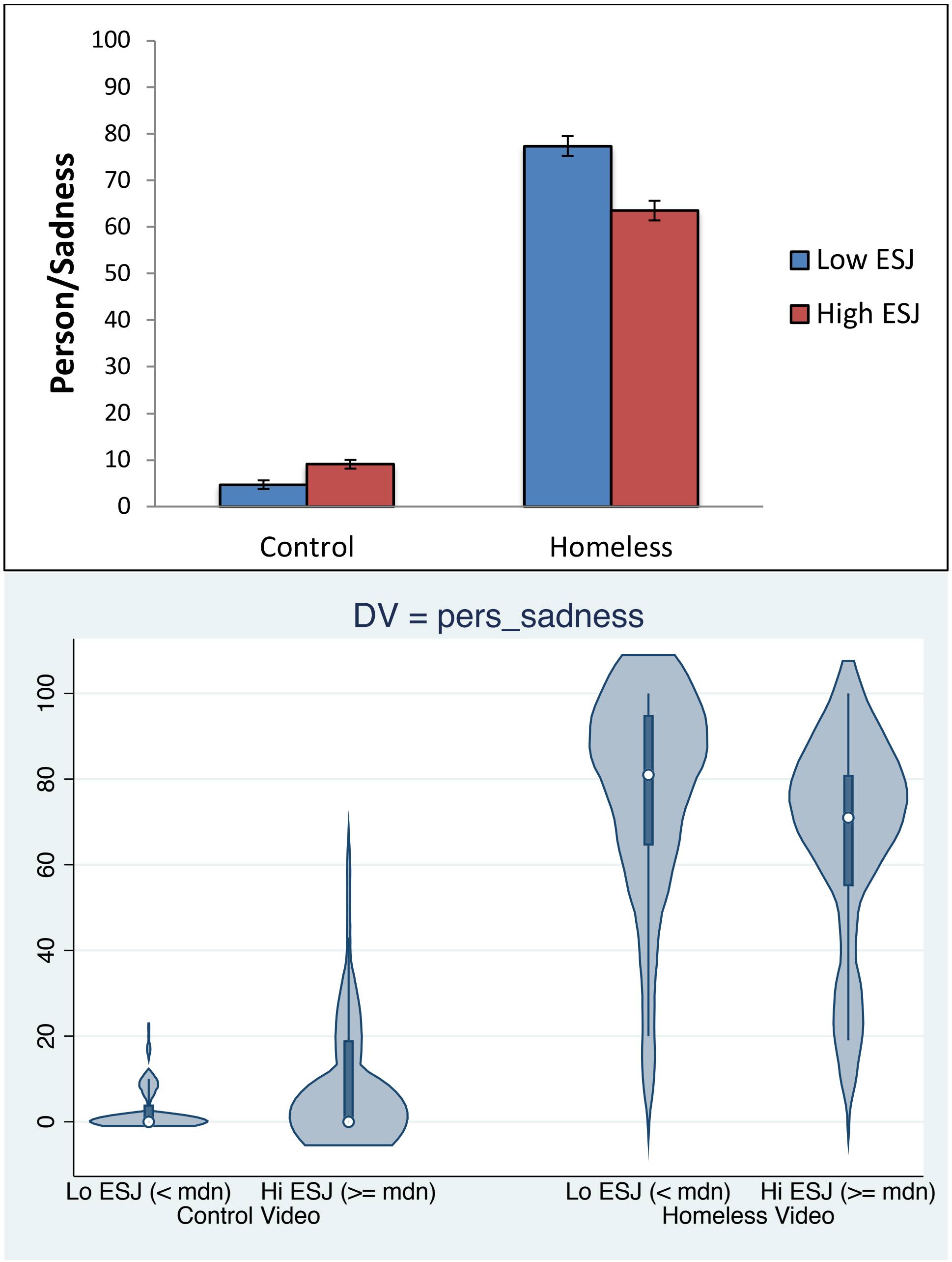

Supplementary Figure 2. *ESJ × Video Type interaction for person-directed sadness in Study 1, p < .001. Simple effect of ESJ in control video condition, p = .03; simple effect of ESJ in homeless video condition, p = .003. Simple effect of video type (homeless vs. control) at low (-1 SD) ESJ, p < .001; simple effect of video type (homeless vs. control) at high (+1 SD) ESJ, p < .001. Emotions were rated on a 0–100 scale. Error bars represent standard errors. Bottom graph shows description statistics and distributional properties based on raw data.*

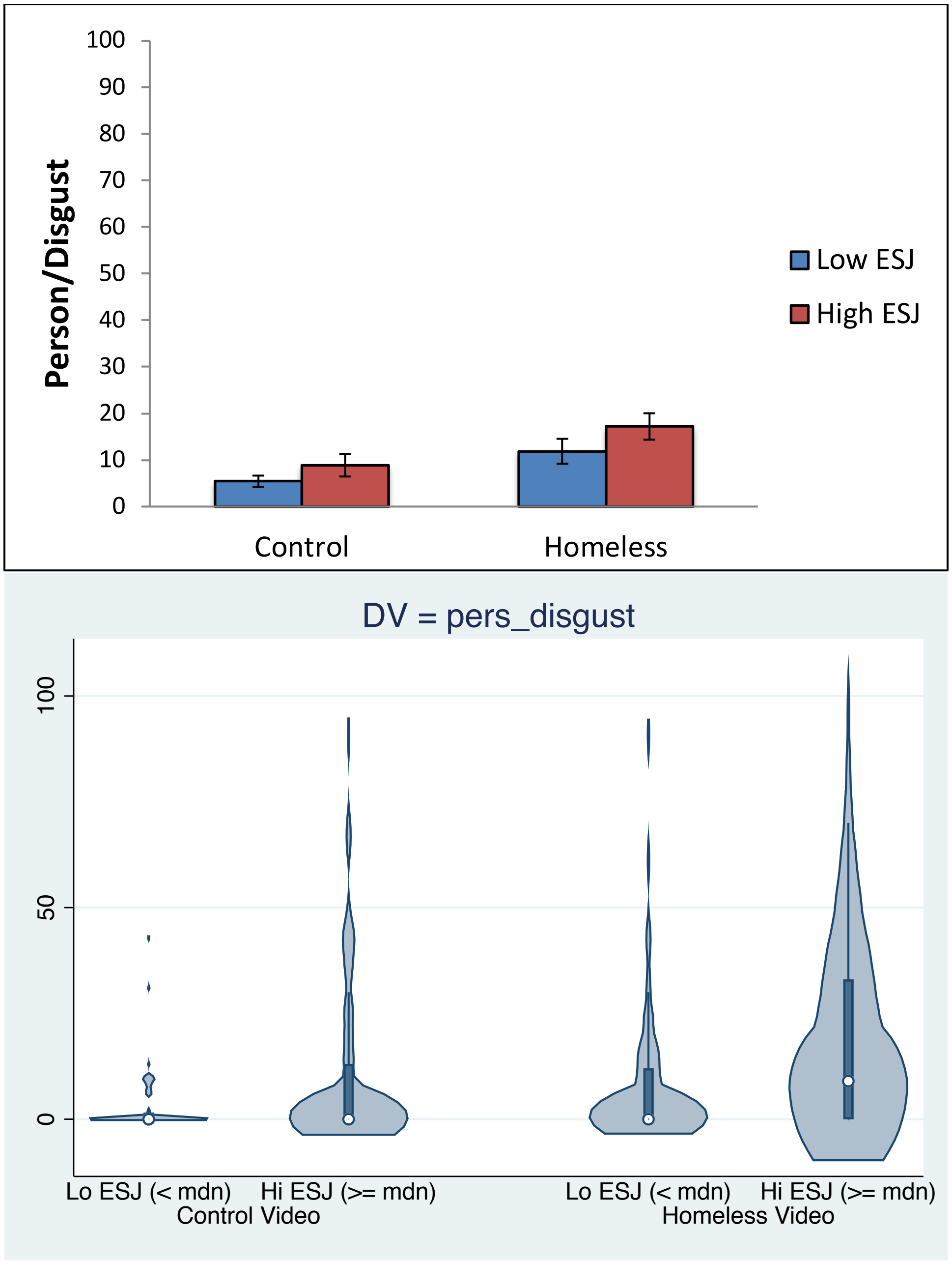

Supplementary Figure 3. *ESJ × Video Type interaction for person-directed disgust in Study 1, p = .62. Simple effect of ESJ in control video condition, p = .149; simple effect of ESJ in homeless video condition, p = .139. Simple effect of video type (homeless vs. control) at low (-1 SD) ESJ, p = .02; simple effect of video type (homeless vs. control) at high (+1 SD) ESJ, p = .004. Emotions were rated on a 0–100 scale. Error bars represent standard errors. Bottom graph shows description statistics and distributional properties based on raw data.*

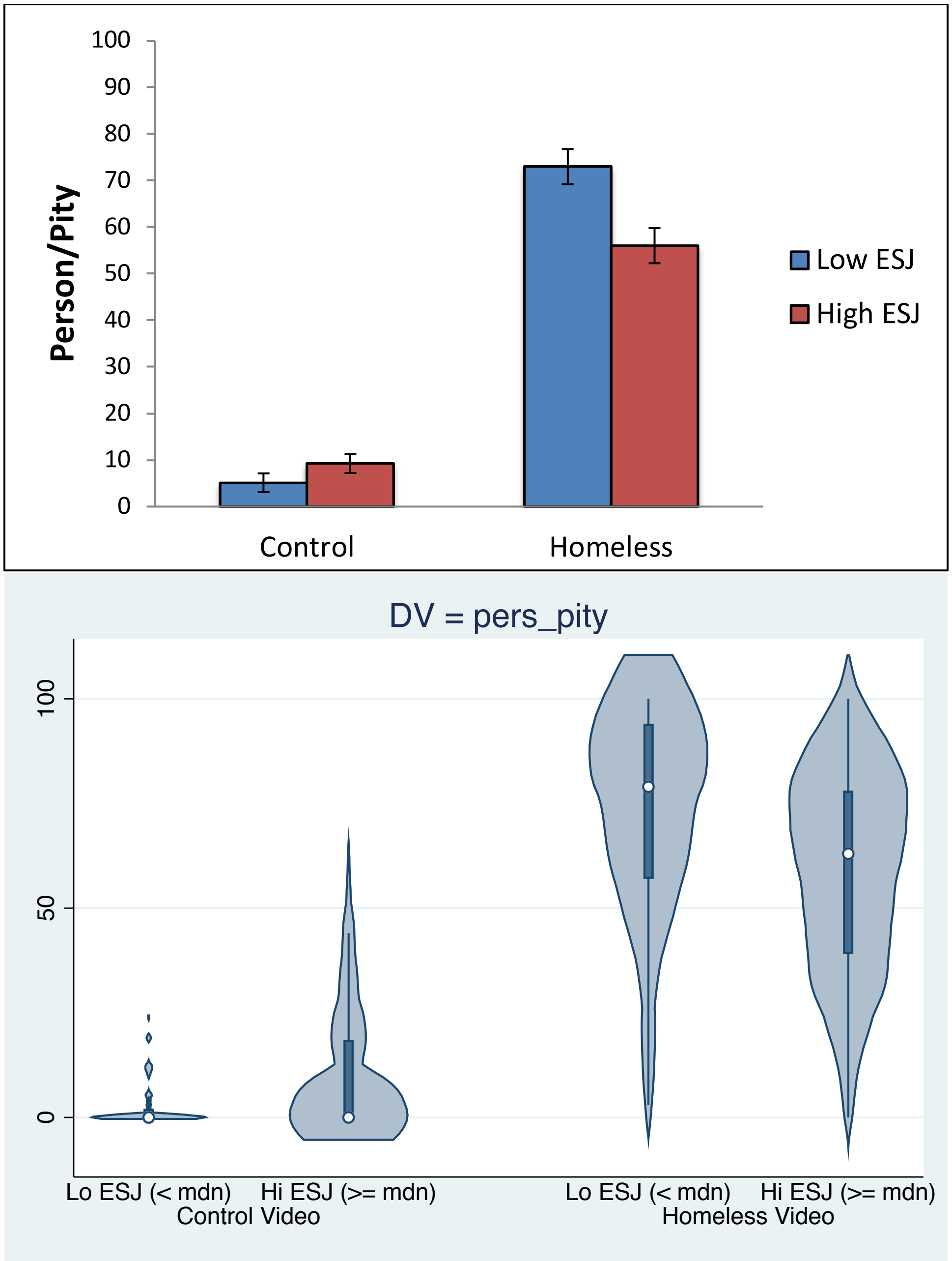

Supplementary Figure 4. *ESJ × Video Type interaction for person-directed pity in Study 1, p < .001. Simple effect of ESJ in control video condition, p = .032; simple effect of ESJ in homeless video condition, p = .002. Simple effect of video type (homeless vs. control) at low (-1 SD) ESJ, p < .001; simple effect of video type (homeless vs. control) at high (+1 SD) ESJ, p < .001. Emotions were rated on a 0–100 scale. Error bars represent standard errors. Bottom graph shows description statistics and distributional properties based on raw data.*

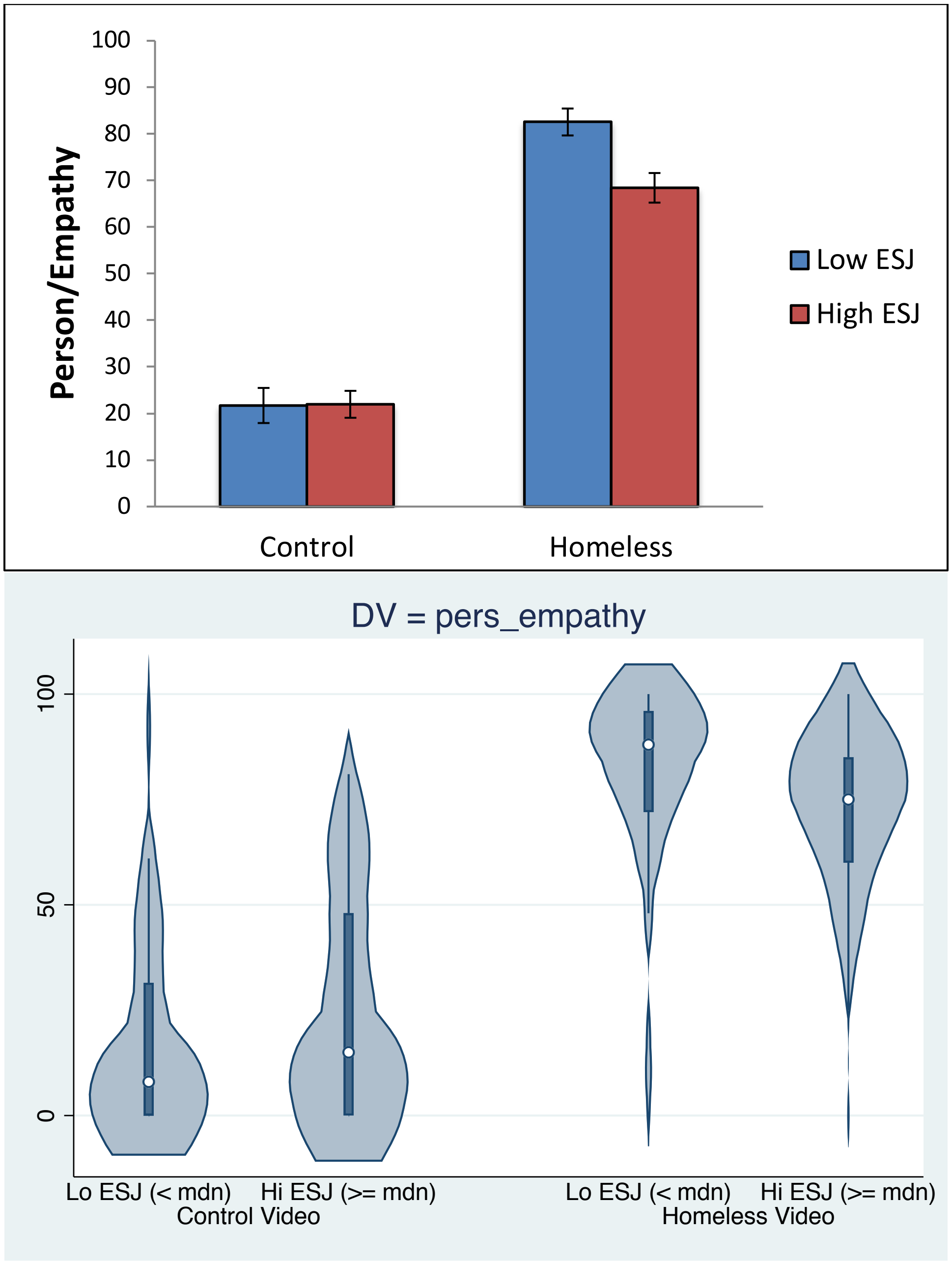

Supplementary Figure 5. *ESJ × Video Type interaction for person-directed empathy in Study 1, p = .016. Simple effect of ESJ in control video condition, p = .959; simple effect of ESJ in homeless video condition, p = .001. Simple effect of video type (homeless vs. control) at low (-1 SD) ESJ, p < .001; simple effect of video type (homeless vs. Emotions were rated on a 0–100 scale. Error bars represent standard errors. Bottom graph shows description statistics and distributional properties based on raw data.*

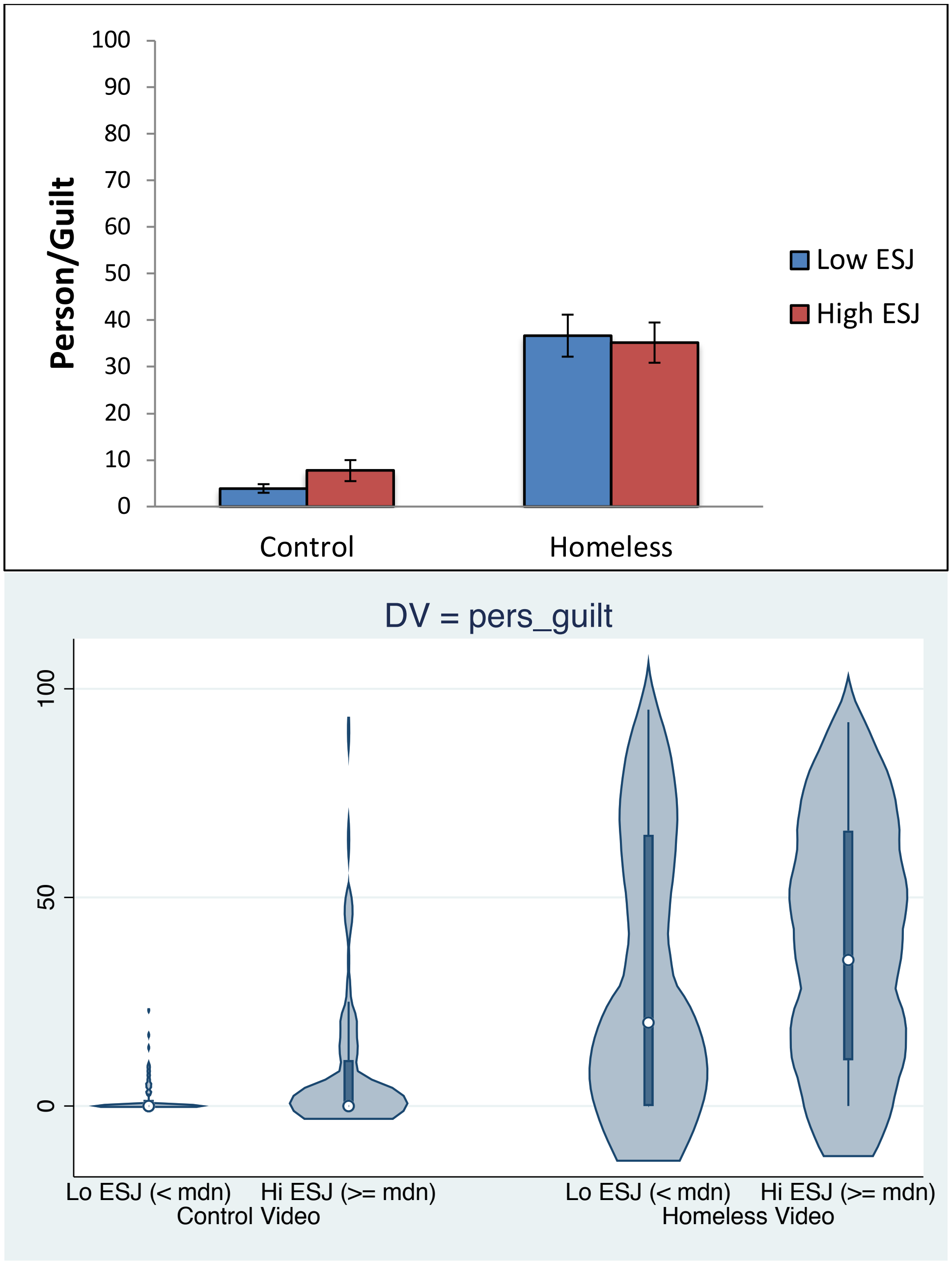

Supplementary Figure 6. *ESJ × Video Type interaction for guilt in Study 1, p = .364. Simple effect of ESJ in control video condition, p = .068; simple effect of ESJ in homeless video condition, p = .821. Simple effect of video type (homeless vs. control) at low (-1 SD) ESJ, p < .001; simple effect of video type (homeless vs. control) at high (+1 SD) ESJ, p = . p < .001. Emotions were rated on a 0–100 scale. Error bars represent standard errors. Bottom graph shows description statistics and distributional properties based on raw data.*

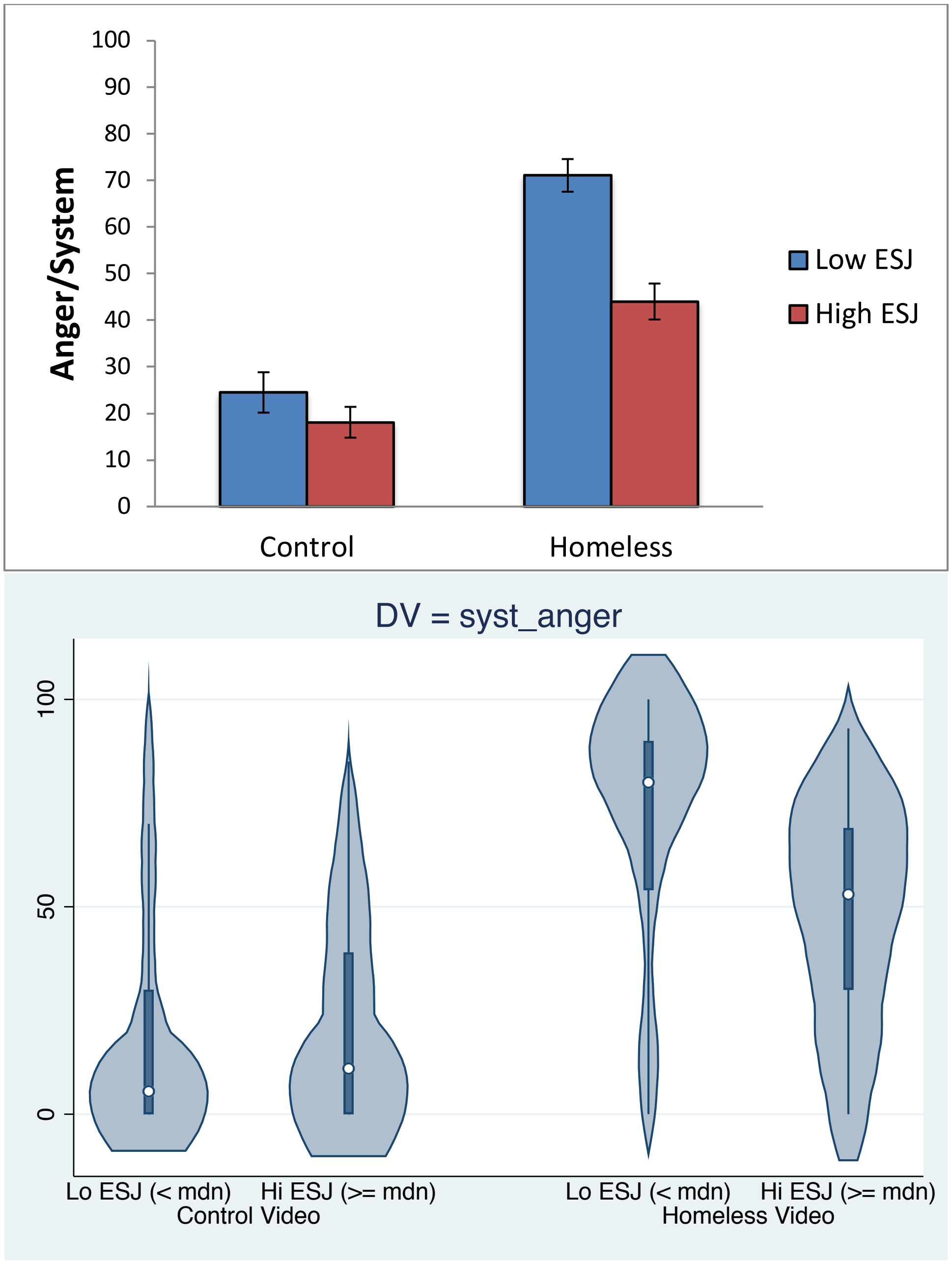

Supplementary Figure 7. *ESJ × Video Type interaction for system-directed anger in Study 1, p < .001. Simple effect of ESJ in control video condition, p = .263; simple effect of ESJ in homeless video condition, p < .001. Simple effect of video type (homeless vs. control) at low (-1 SD) ESJ, p < .001; simple effect of video type (homeless vs. control) at high (+1 SD) ESJ, p < .001. Emotions were rated on a 0–100 scale. Error bars represent standard errors. Bottom graph shows description statistics and distributional properties based on raw data.*

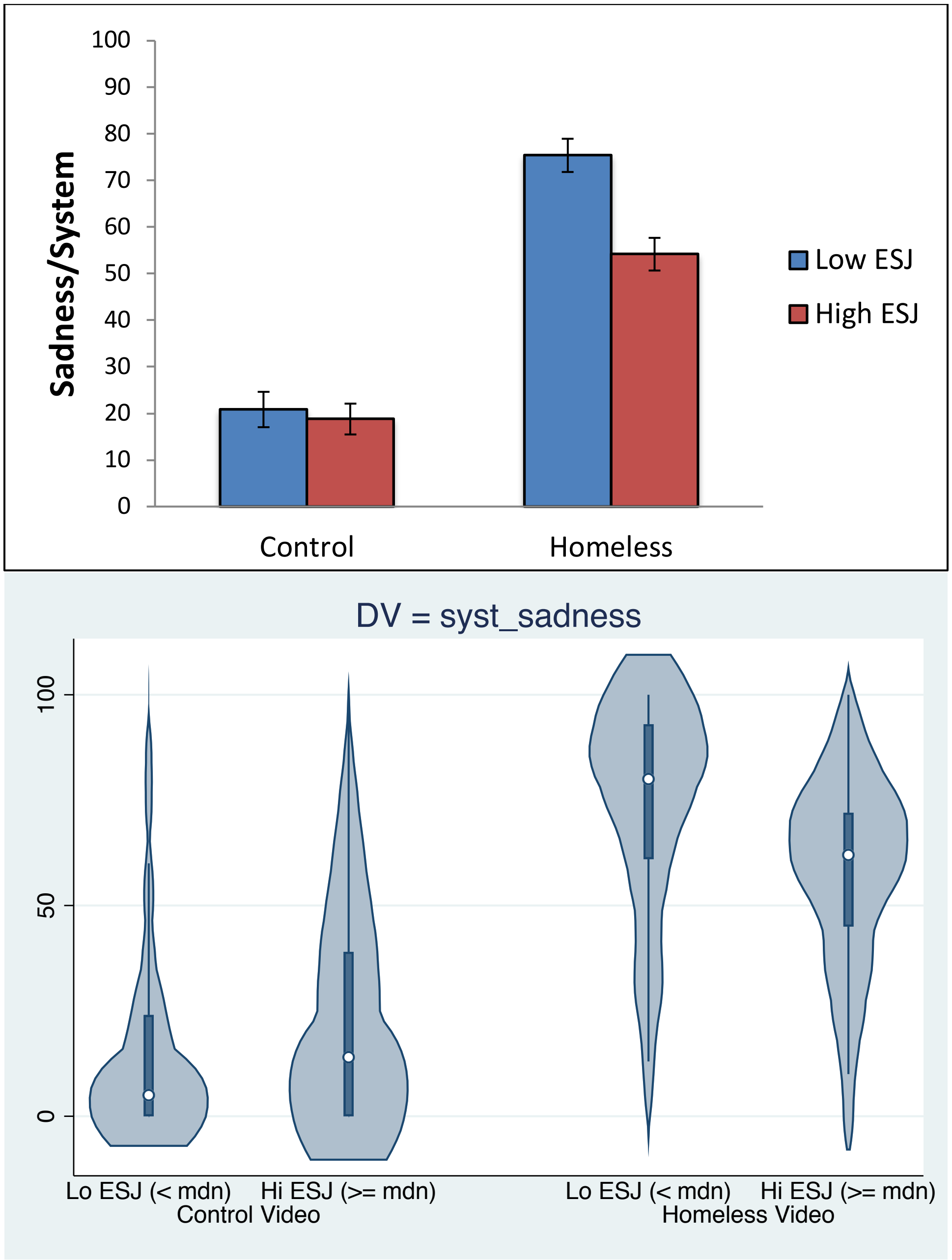

Supplementary Figure 8. *ESJ × Video Type interaction for system-directed sadness in Study 1, p = .001. Simple effect of ESJ in control video condition, p = .695; simple effect of ESJ in homeless video condition, p < .001. Simple effect of video type (homeless vs. control) at low (-1 SD) ESJ, p < .001; simple effect of video type (homeless vs. control) at high (+1 SD) ESJ, p < .001. Emotions were rated on a 0–100 scale. Error bars represent standard errors. Bottom graph shows description statistics and distributional properties based on raw data.*

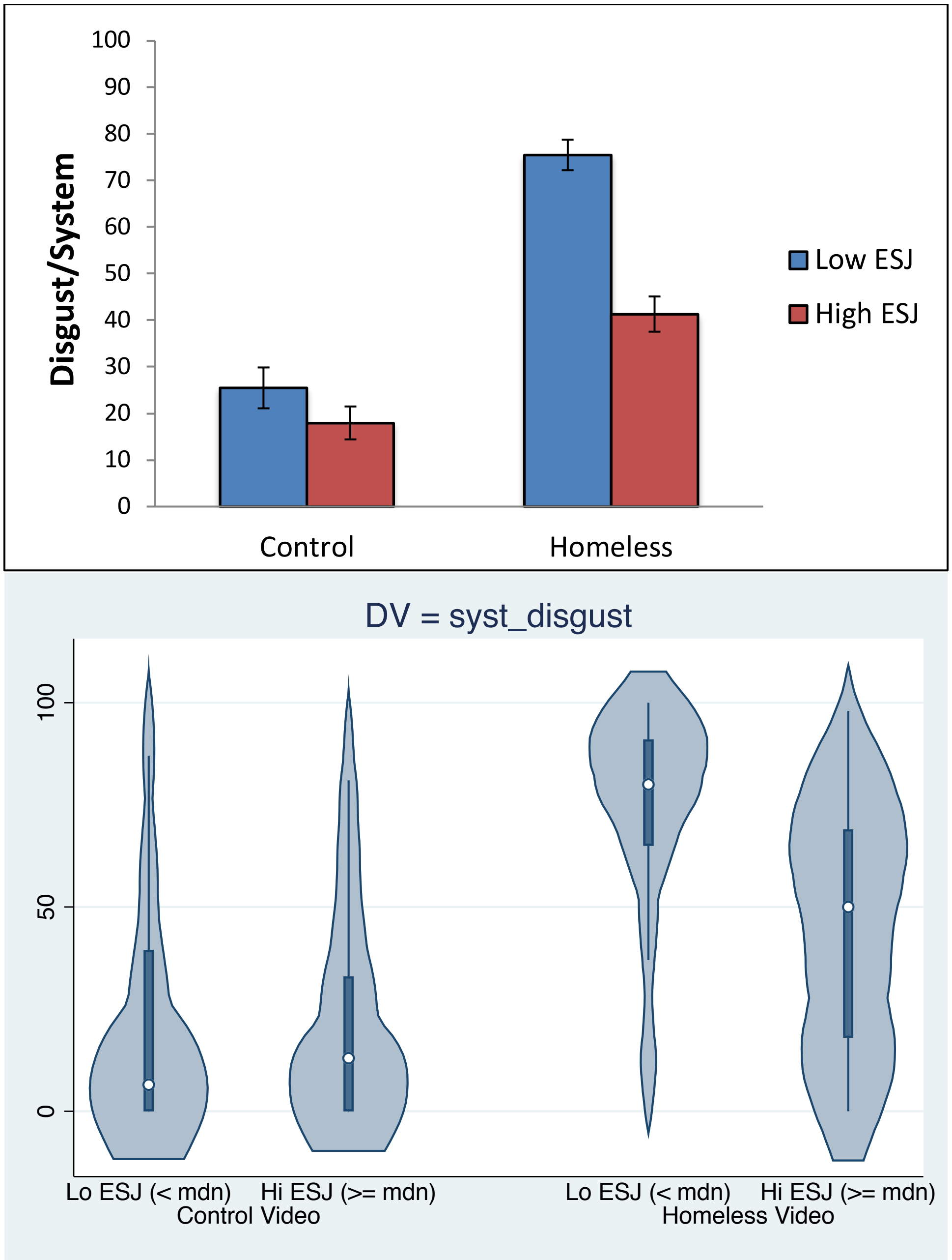

Supplementary Figure 9. *ESJ × Video Type interaction for system-directed disgust in Study 1, p < .001. Simple effect of ESJ in control video condition, p = .207; simple effect of ESJ in homeless video condition p < .001. Simple effect of video type (homeless vs. control) at low (-1 SD) ESJ, p < .001; simple effect of video type (homeless vs. control) at high (+1 SD) ESJ, p < .001. Emotions were rated on a 0–100 scale. Error bars represent standard errors. Bottom graph shows description statistics and distributional properties based on raw data.*

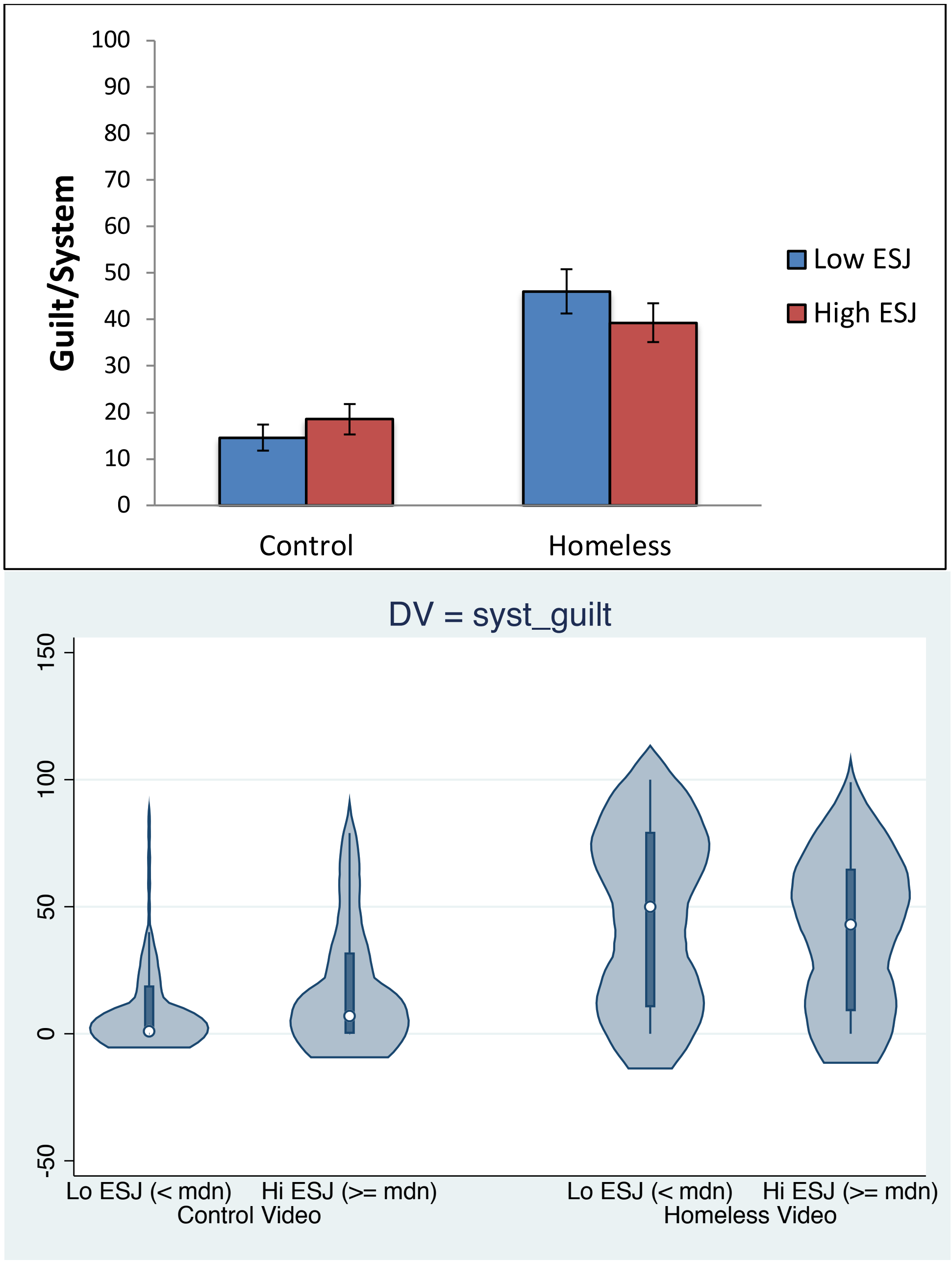

Supplementary Figure 10. *ESJ × Video Type interaction for system-directed guilt in Study 1, p = .065. Simple effect of ESJ in control video condition, p = .343; simple effect of ESJ in homeless video condition, p = .311. Simple effect of video type (homeless vs. control) at low (-1 SD) ESJ, p < .001; simple effect of video type (homeless vs. control) at high (+1 SD) ESJ, p < .001. Emotions were rated on a 0–100 scale. Error bars represent standard errors. Bottom graph shows description statistics and distributional properties based on raw data.*

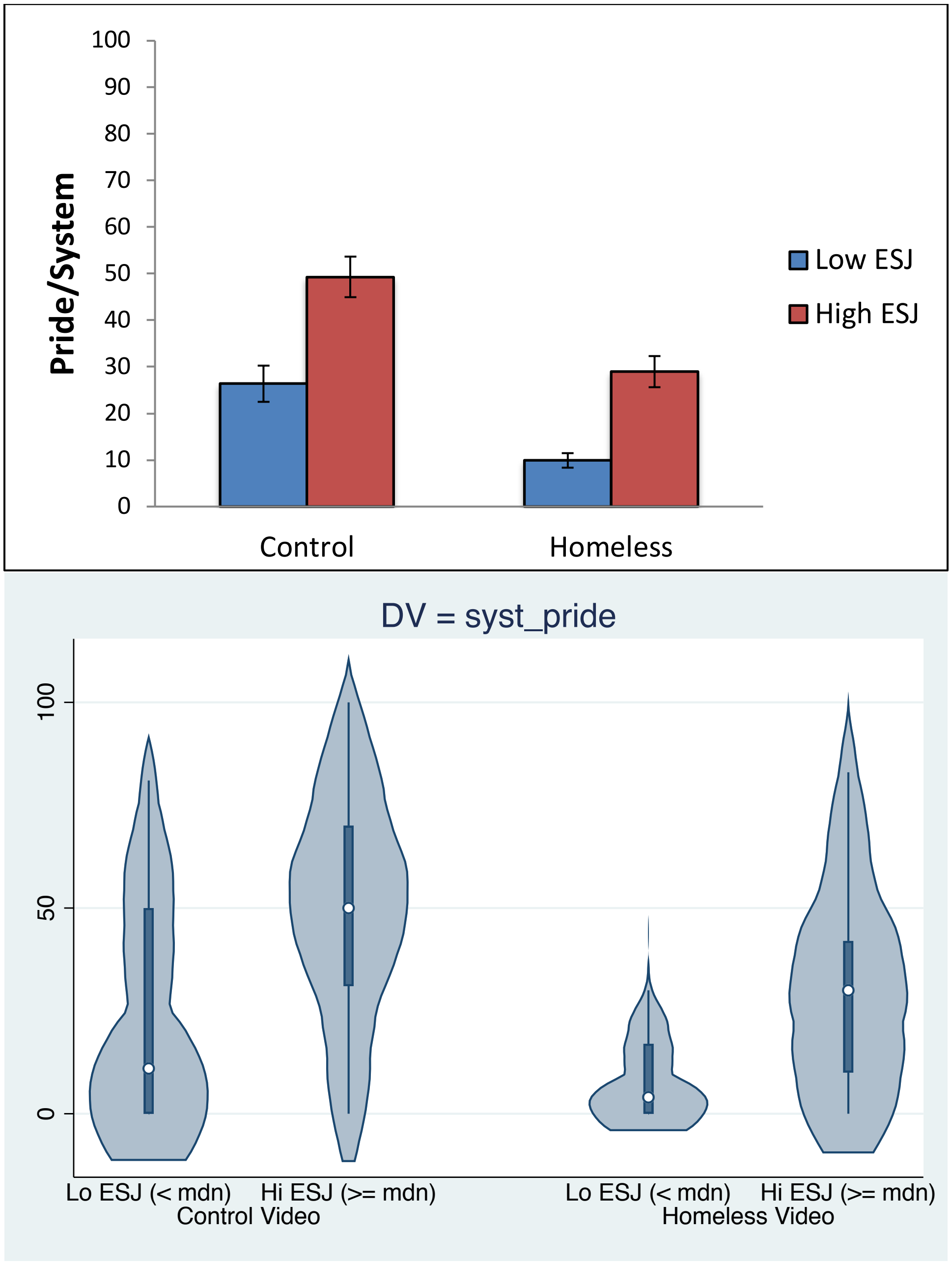

Supplementary Figure 11. *ESJ × Video Type interaction for system-directed pride in Study 1, p = .470. Simple effect of ESJ in control video condition, p < .001; simple effect of ESJ in homeless video condition, p < .001. Simple effect of video type (homeless vs. control) at low (-1 SD) ESJ, p < .001; simple effect of video type (homeless vs. control) at high (+1 SD) ESJ, p < .001. Emotions were rated on a 0–100 scale. Error bars represent standard errors. Bottom graph shows description statistics and distributional properties based on raw data.*

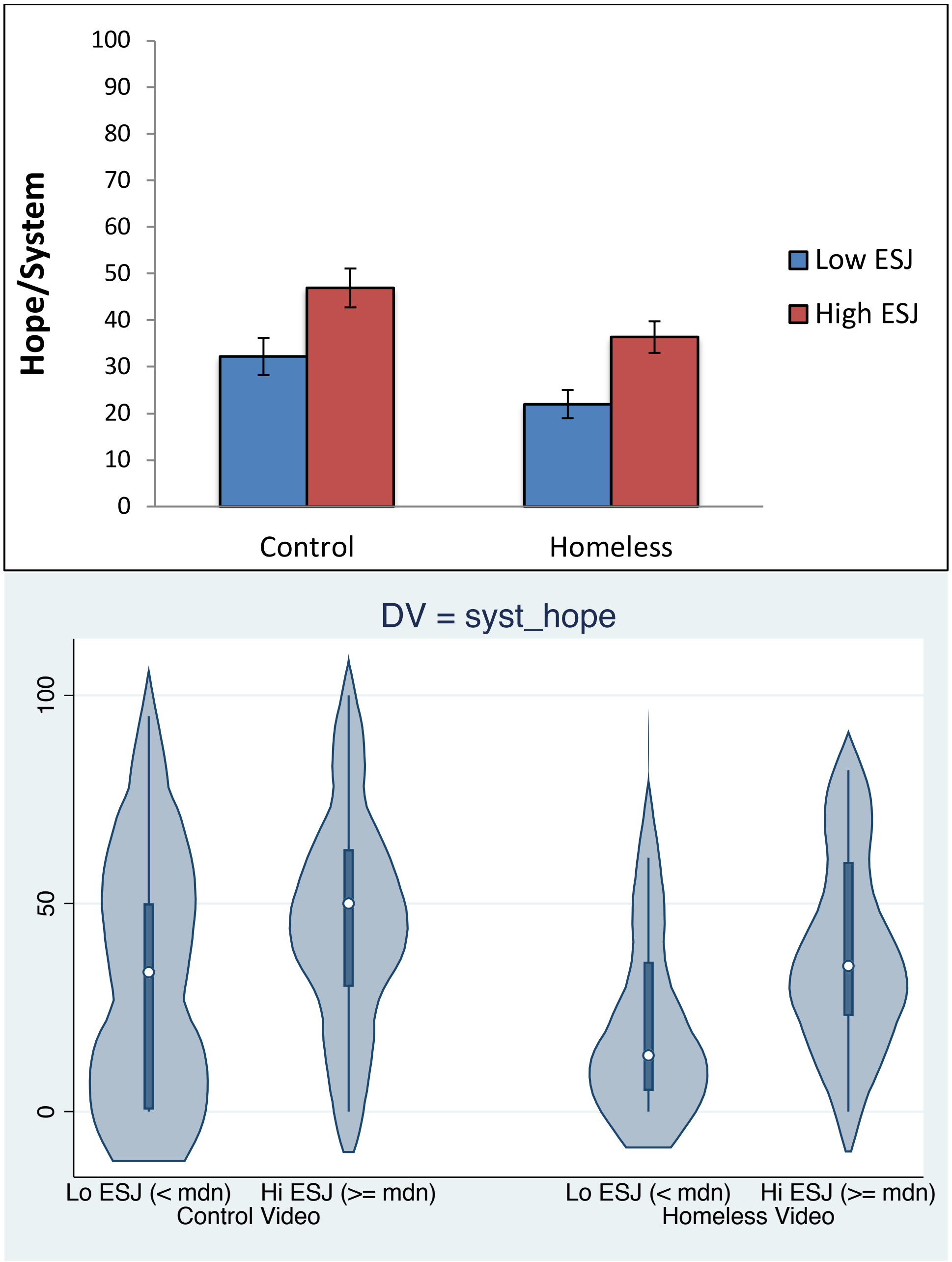

Supplementary Figure 12. *ESJ × Video Type interaction for system-directed hope in Study 1, p = .986. Simple effect of ESJ in control video condition, p = .013; simple effect of ESJ in homeless video condition, p = .002. Simple effect of video type (homeless vs. control) at low (-1 SD) ESJ, p = .014; simple effect of video type (homeless vs. control) at high (+1 SD) ESJ, p = .003. Emotions were rated on a 0–100 scale. Error bars represent standard errors. Bottom graph shows description statistics and distributional properties based on raw data.*

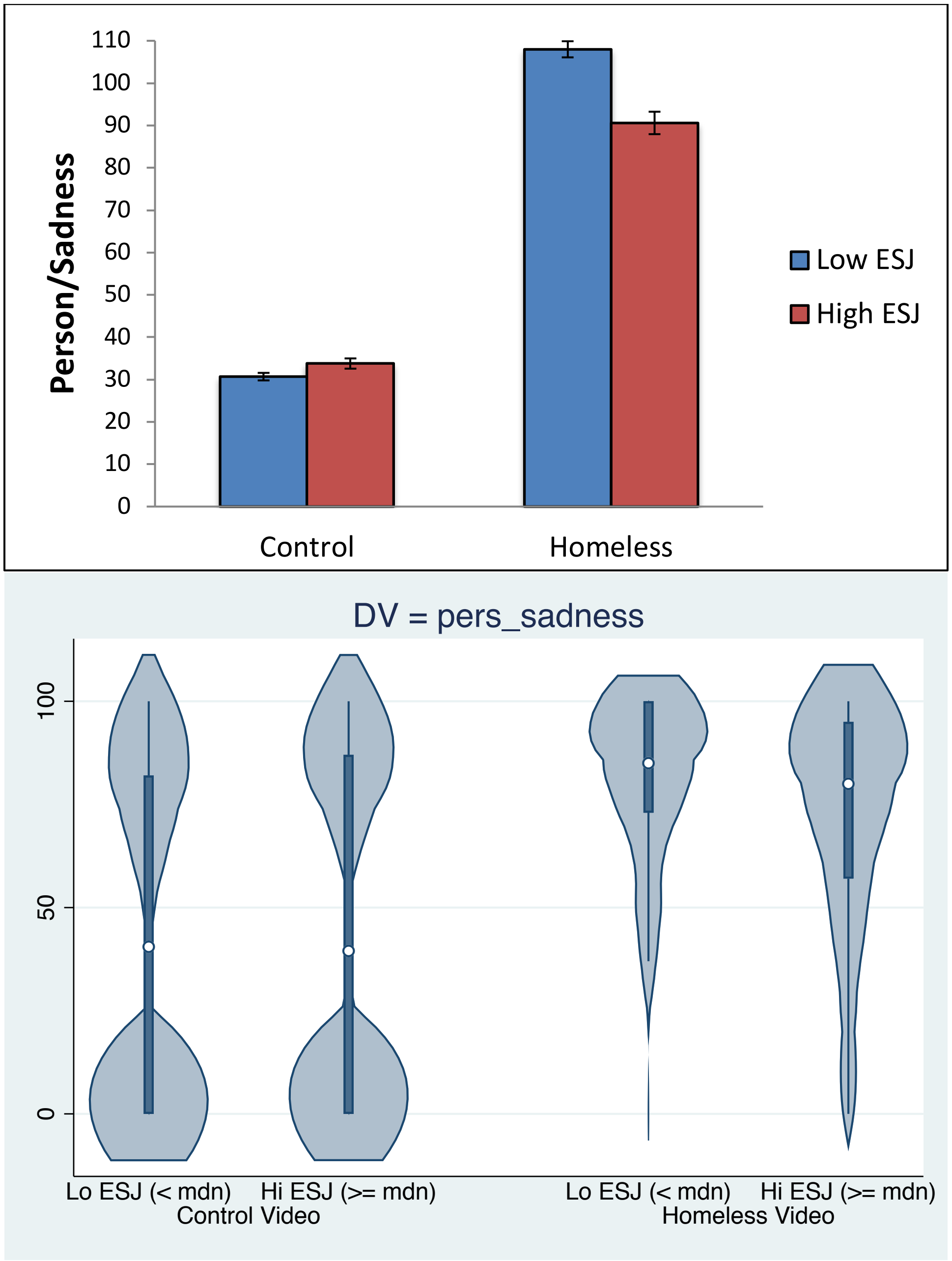

Supplementary Figure 13. *ESJ × Video Type interaction for person-directed sadness in Study 2, p < .001. Simple effect of ESJ in control video condition, p = .002; simple effect of ESJ in homeless video condition, p < .001. Simple effect of video type (homeless vs. control) at low (-1 SD) ESJ, p < .001; simple effect of video type (homeless vs. control) at high (+1 SD) ESJ, p < .001. Emotions were rated on a 0–100 scale. Error bars represent standard errors. Bottom graph shows description statistics and distributional properties based on raw data.*

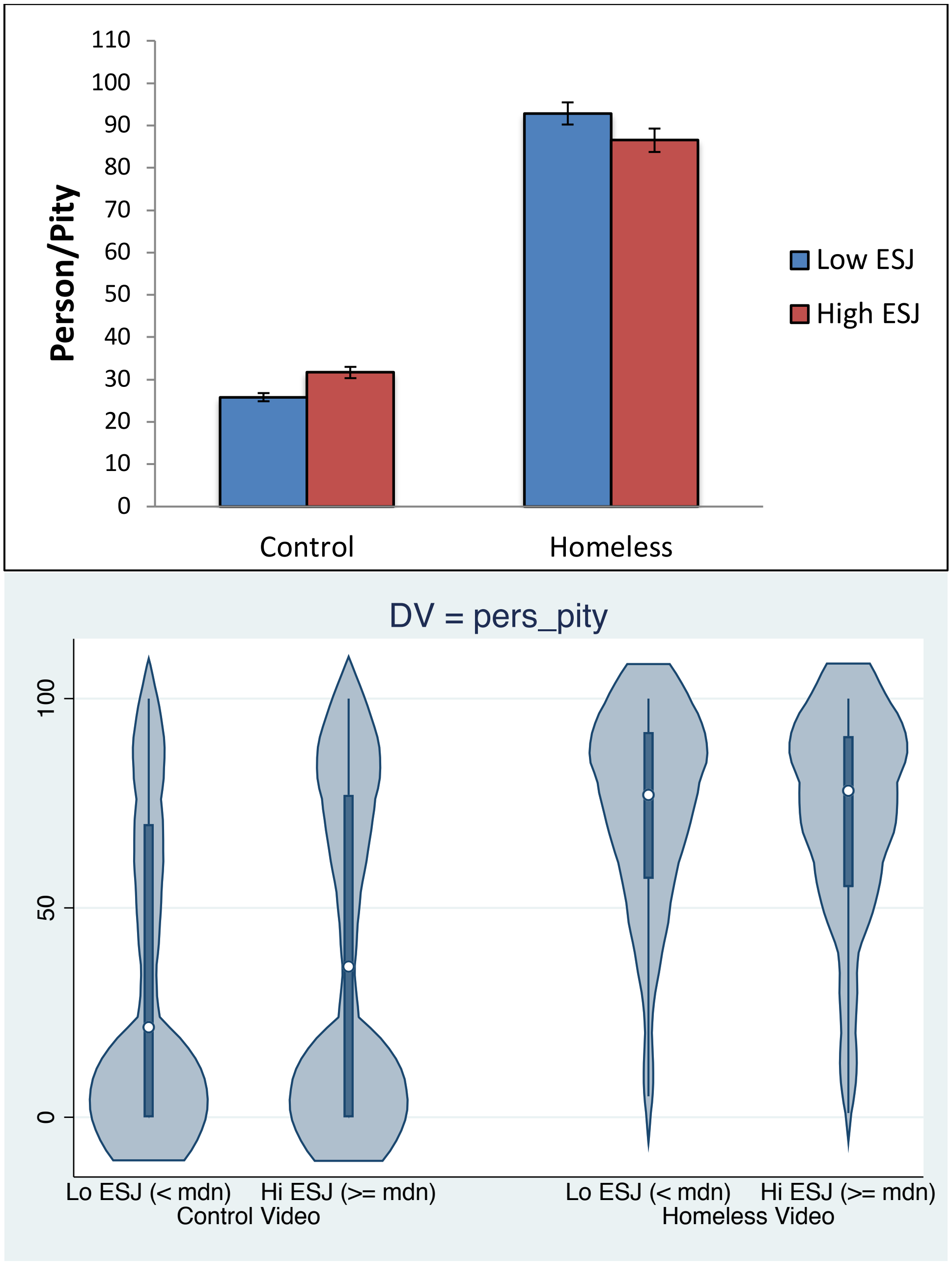

Supplementary Figure 14. *ESJ × Video Type interaction for person-directed pity in Study 2, p = .001. Simple effect of ESJ in control video condition, p < .001; simple effect of ESJ in homeless video condition, p = .127. Simple effect of video type (homeless vs. control) at low (-1 SD) ESJ, p < .001; simple effect of video type (homeless vs. control) at high (+1 SD) ESJ, p < .001. Emotions were rated on a 0–100 scale. Error bars represent standard errors. Bottom graph shows description statistics and distributional properties based on raw data.*

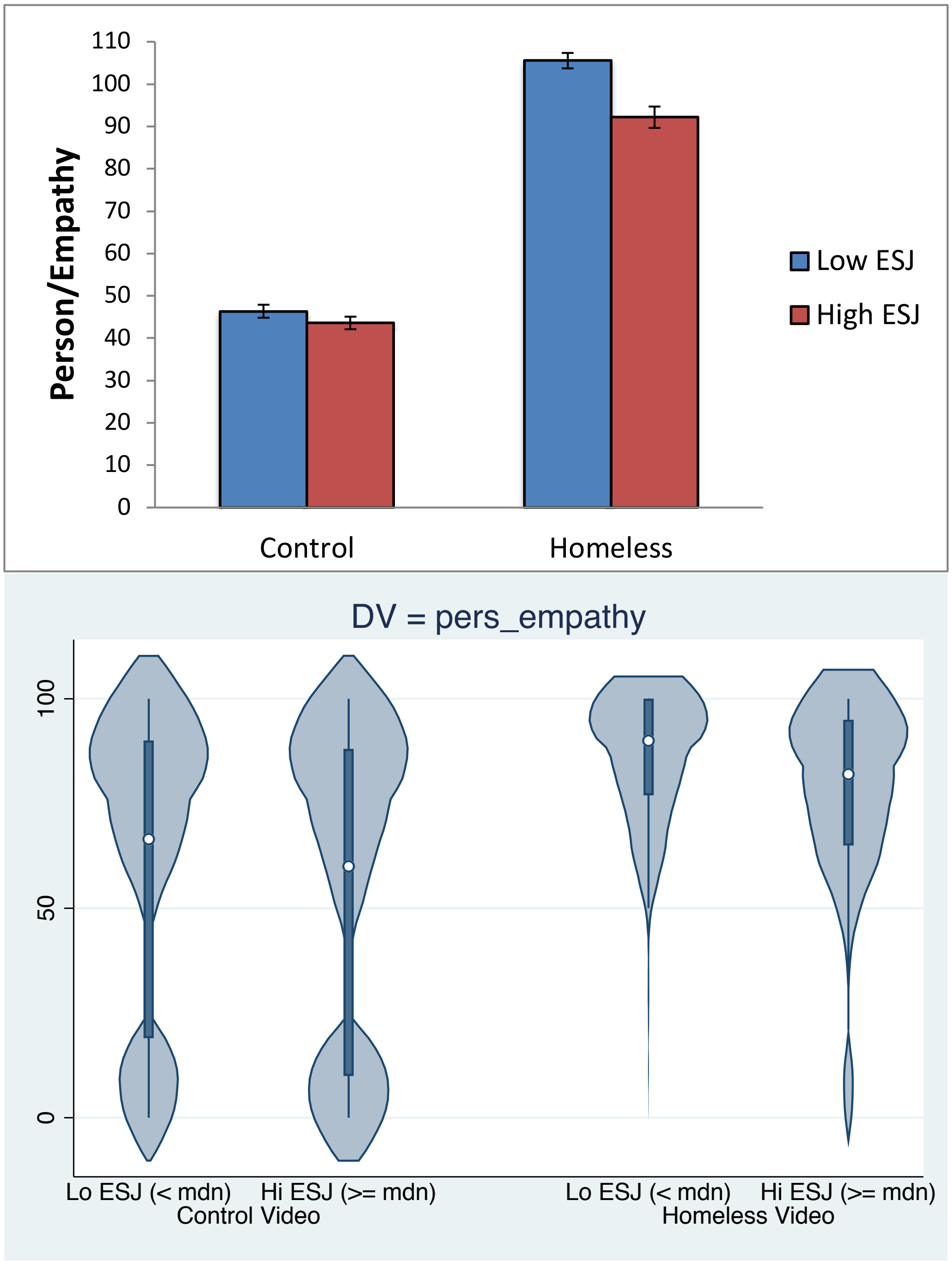

Supplementary Figure 15. *ESJ × Video Type interaction for person-directed empathy in Study 2, p = .011. Simple effect of ESJ in control video condition, p = .55; simple effect of ESJ in homeless video condition, p < .001. Simple effect of video type (homeless vs. control) at low (-1 SD) ESJ, p < .001; simple effect of video type (homeless vs. control) at high (+1 SD) ESJ, p < .001. Emotions were rated on a 0–100 scale. Error bars represent standard errors. Bottom graph shows description statistics and distributional properties based on raw data.*

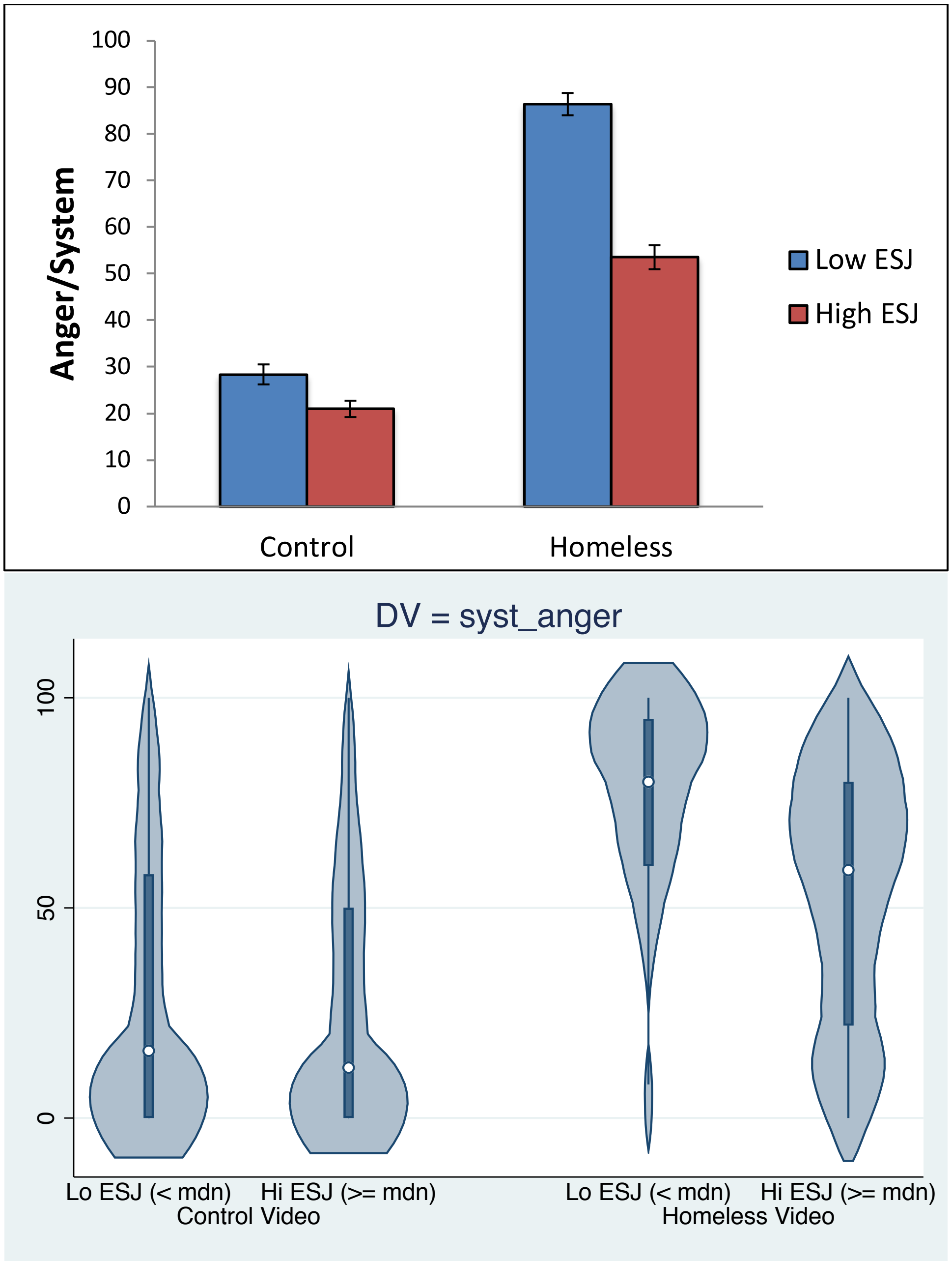

Supplementary Figure 16. *ESJ × Video Type interaction for system-directed anger in Study 2, p < .001. Simple effect of ESJ in control video condition, p = .294; simple effect of ESJ in homeless video condition, p < .001. Simple effect of video type (homeless vs. control) at low (-1 SD) ESJ, p < .001; simple effect of video type (homeless vs. control) at high (+1 SD) ESJ, p < .001. Emotions were rated on a 0–100 scale. Error bars represent standard errors. Bottom graph shows description statistics and distributional properties based on raw data.*

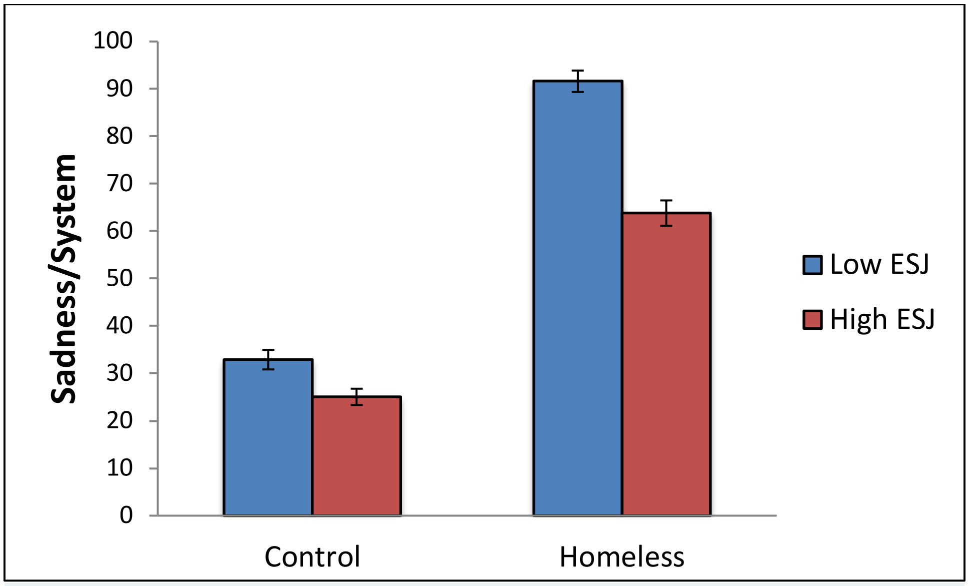

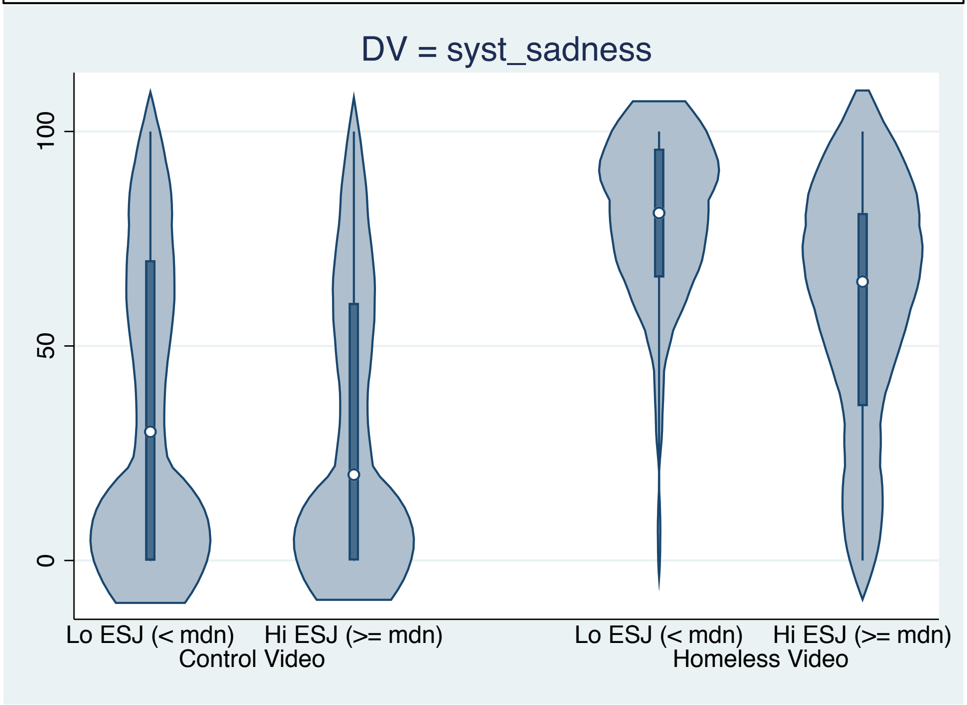

Supplementary Figure 17. *ESJ × Video Type interaction for system-directed sadness in Study 2, p < .001. Simple effect of ESJ in control video condition, p = .134; simple effect of ESJ in homeless video condition, p < .001. Simple effect of video type (homeless vs. control) at low (-1 SD) ESJ, p < .001; simple effect of video type (homeless vs. control) at high (+1 SD) ESJ, p < .001. Emotions were rated on a 0–100 scale. Error bars represent standard errors. Bottom graph shows description statistics and distributional properties based on raw data.*

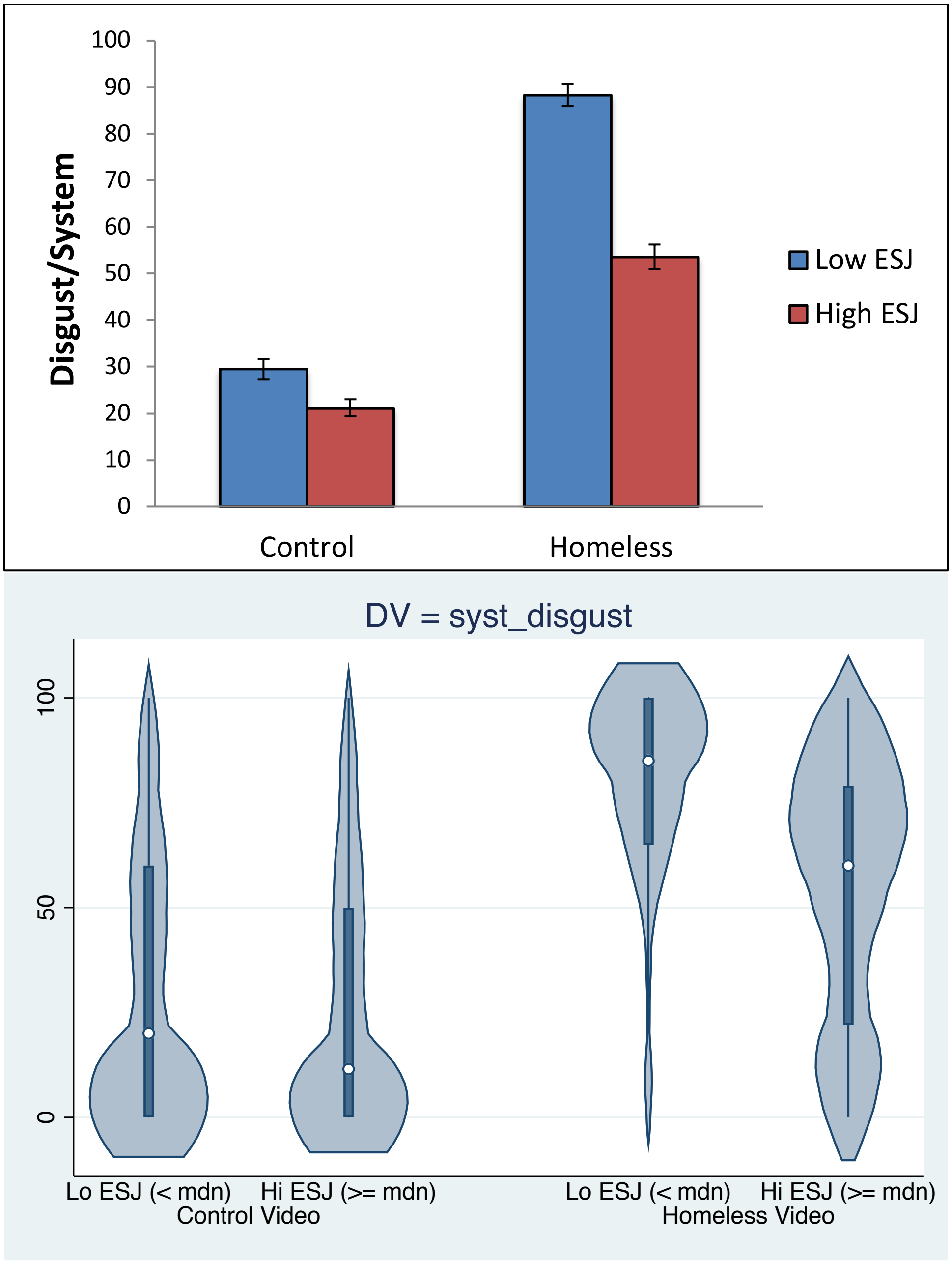

Supplementary Figure 18. *ESJ × Video Type interaction for system-directed disgust in Study 2, $p < .001$. Simple effect of ESJ in control video condition, $p = .201$; simple effect of ESJ in homeless video condition, $p < .001$. Simple effect of video type (homeless vs. control) at low (-1 SD) ESJ, $p < .001$; simple effect of video type (homeless vs. control) at high (+1 SD) ESJ, $p < .001$. Emotions were rated on a 0–100 scale. Error bars represent standard errors. Bottom graph shows description statistics and distributional properties based on raw data.*

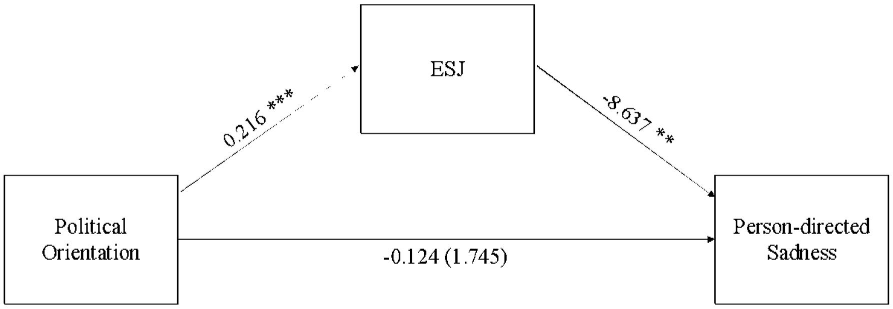

Supplementary Figure 19. *Regression coefficients for the relationship between political orientation and person-directed sadness as mediated by Economic System Justification (ESJ) in Study 1. The regression coefficient between political orientation and person-directed sadness, adjusting for ESJ, is in parentheses.* ** $p < .01$, *** $p < .001$.

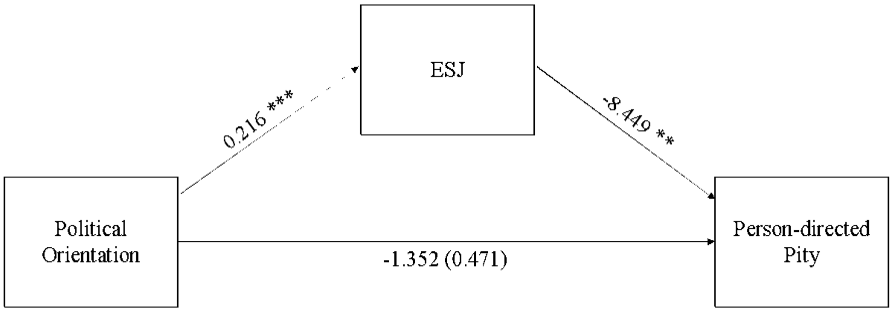

Supplementary Figure 20. *Regression coefficients for the relationship between political orientation and person-directed pity as mediated by Economic System Justification (ESJ) in Study 1. The regression coefficient between political orientation and person-directed pity, adjusting for ESJ, is in parentheses.* ** $p < .01$, *** $p < .001$.

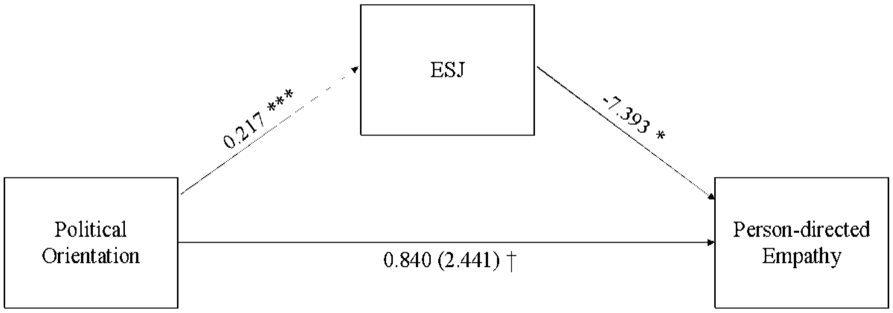

Supplementary Figure 21. *Regression coefficients for the relationship between political orientation and person-directed empathy as mediated by Economic System Justification (ESJ) in Study 1. The regression coefficient between political orientation and person-directed empathy, adjusting for ESJ, is in parentheses.* † *p* < .1, * *p* < .05, *** *p* < .001.

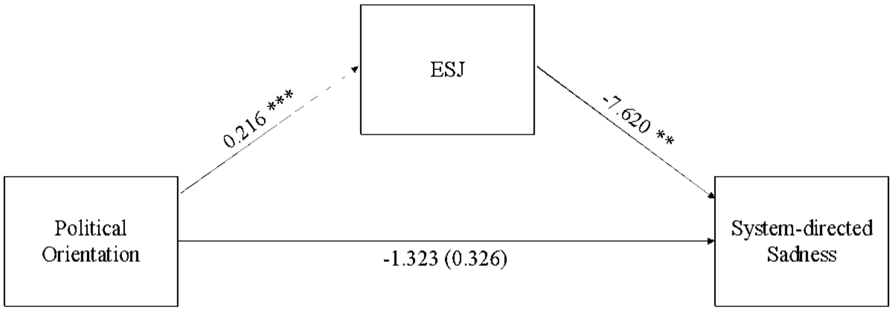

Supplementary Figure 22. *Regression coefficients for the relationship between political orientation and system-directed sadness as mediated by Economic System Justification (ESJ) in Study 1. The regression coefficient between political orientation and system-directed sadness, adjusting for ESJ, is in parentheses.* ** *p* < .01, *** *p* < .001.

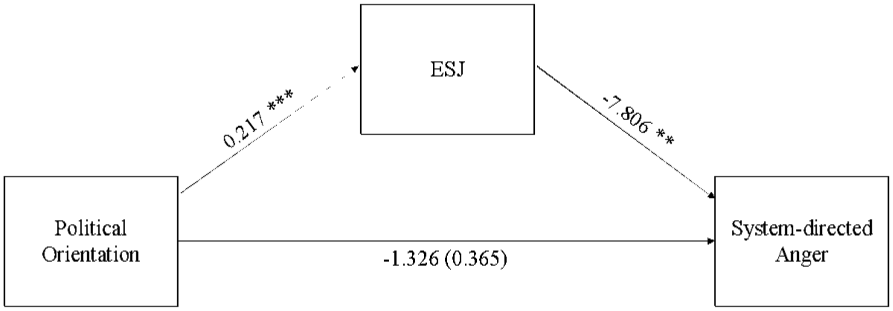

Supplementary Figure 23. *Regression coefficients for the relationship between political orientation and system-directed anger as mediated by Economic System Justification (ESJ) in Study 1. The regression coefficient between political orientation and system-directed anger, adjusting for ESJ, is in parentheses.* ** p < .01, *** p < .001

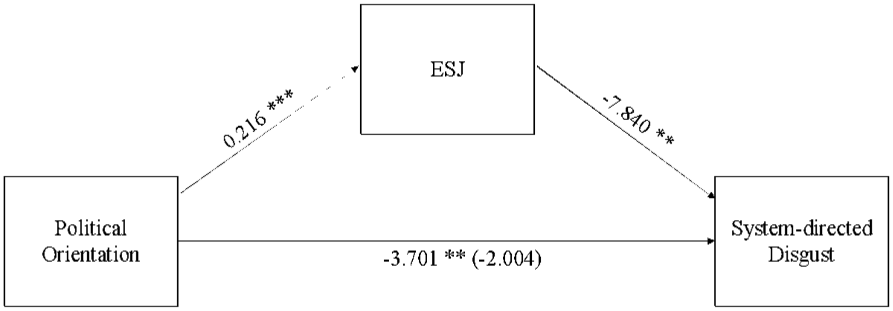

Supplementary Figure 24. *Regression coefficients for the relationship between political orientation and system-directed disgust as mediated by Economic System Justification (ESJ) in Study 1. The regression coefficient between political orientation and system-directed disgust, adjusting for ESJ, is in parentheses.* ** p < .01, *** p < .001.

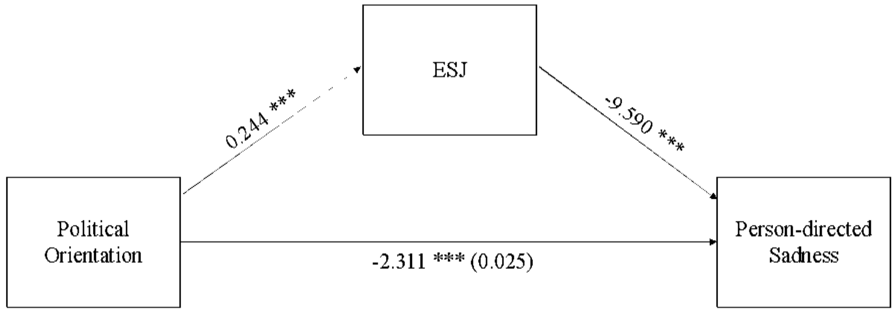

Supplementary Figure 25. *Regression coefficients for the relationship between political orientation and person-directed sadness as mediated by Economic System Justification (ESJ) in Study 2. The regression coefficient between political orientation and person-directed sadness, adjusting for ESJ, is in parentheses.* *** $p < .001$.

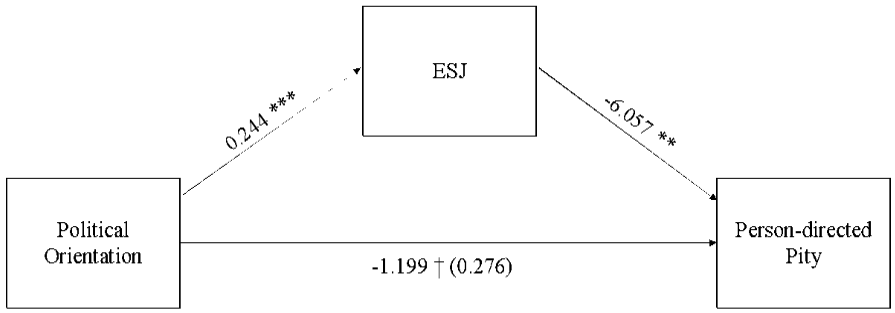

Supplementary Figure 26. *Regression coefficients for the relationship between political orientation and person-directed pity as mediated by Economic System Justification (ESJ) in Study 2. The regression coefficient between political orientation and person-directed pity, adjusting for ESJ, is in parentheses.* † $p < .1$, ** $p < .01$, *** $p < .001$.

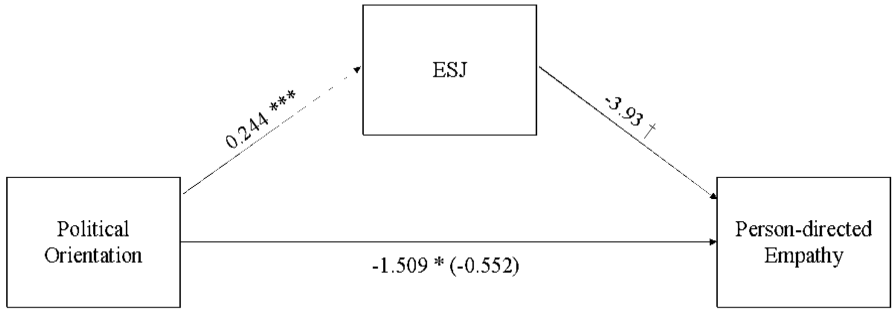

Supplementary Figure 27. *Regression coefficients for the relationship between political orientation and person-directed empathy as mediated by Economic System Justification (ESJ) in Study 2. The regression coefficient between political orientation and person-directed empathy, adjusting for ESJ, is in parentheses.* † $p < .1$, * $p < .05$, *** $p < .001$.

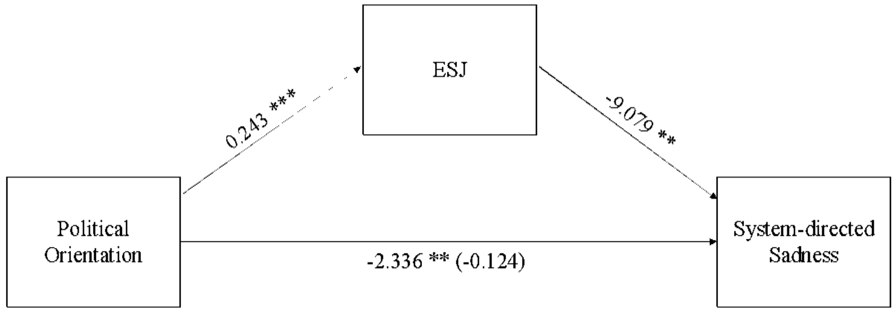

Supplementary Figure 28. *Regression coefficients for the relationship between political orientation and system-directed sadness as mediated by Economic System Justification (ESJ) in Study 2. The regression coefficient between political orientation and system-directed sadness, adjusting for ESJ, is in parentheses.* ** $p < .01$, *** $p < .001$.

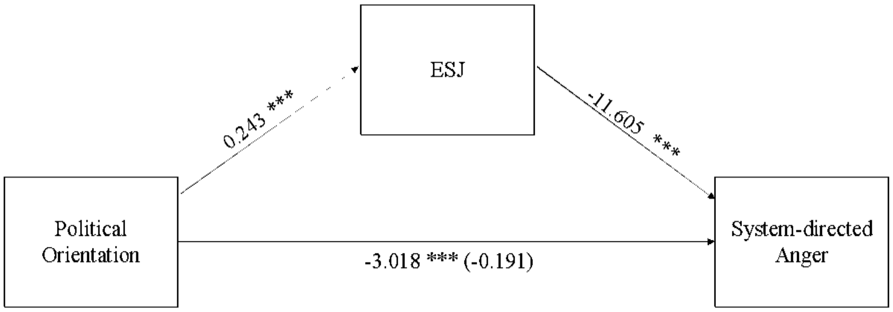

Supplementary Figure 29. *Regression coefficients for the relationship between political orientation and system-directed anger as mediated by Economic System Justification (ESJ) in Study 2. The regression coefficient between political orientation and system-directed anger, adjusting for ESJ, is in parentheses. *** p <* .001.

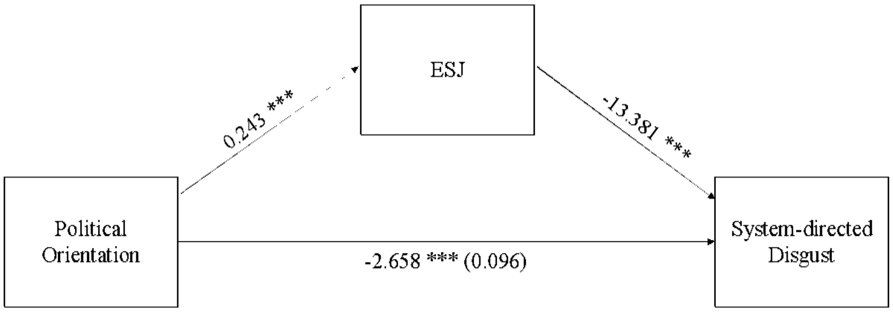

Supplementary Figure 30. *Regression coefficients for the relationship between political orientation and system-directed disgust as mediated by Economic System Justification (ESJ) in Study 2. The regression coefficient between political orientation and system-directed disgust, adjusting for ESJ, is in parentheses. *** p <* .001.

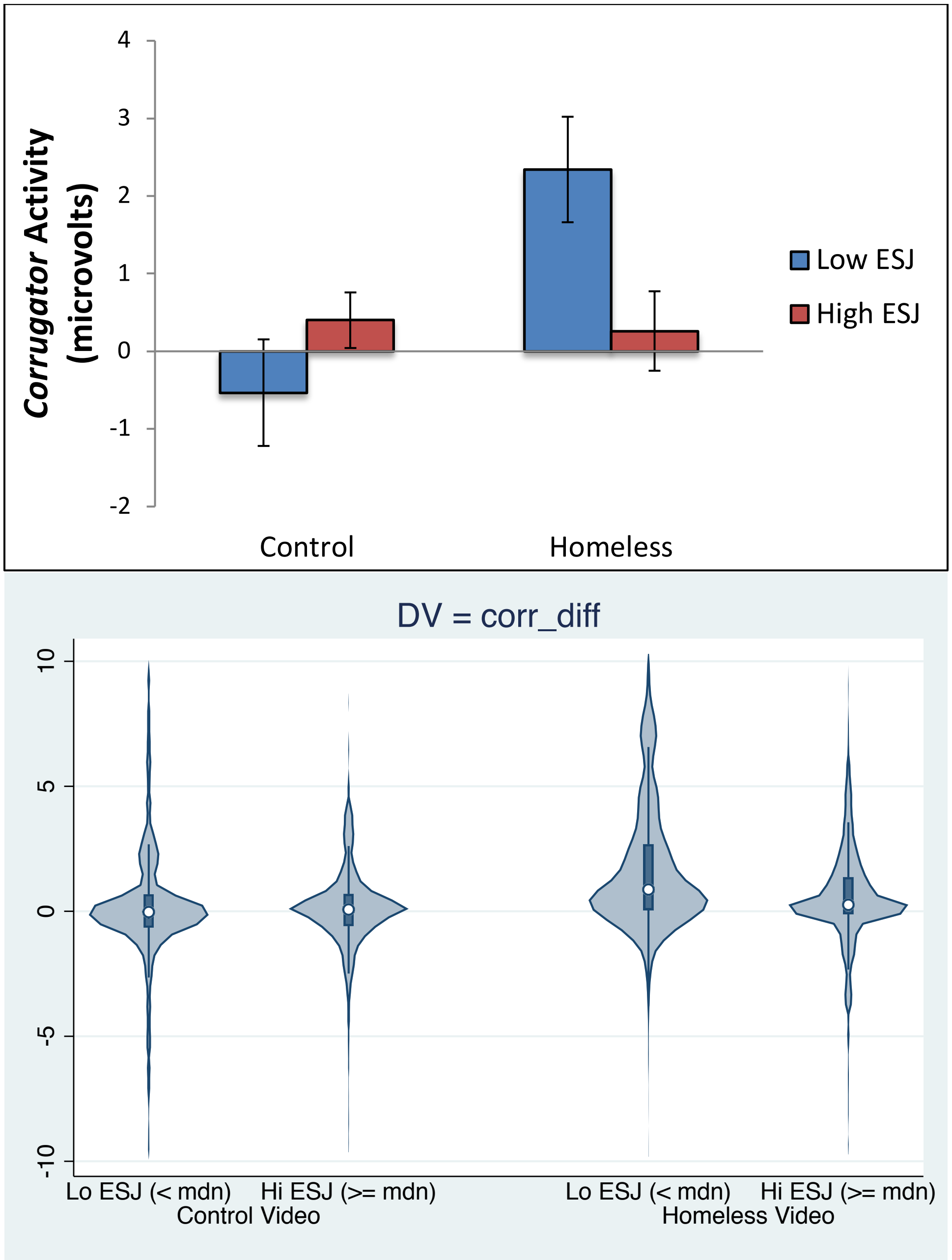

Supplementary Figure 31. *ESJ × Video Type interaction for corrugator activity in Study 3, p = .041. Simple effect of ESJ in control video condition, p = .22; simple effect of ESJ in homeless video condition, p = .017. Simple effect of video type (homeless vs. control) at low (-1 SD) ESJ, p = .022; simple effect of video type (homeless vs. control) at high (+1 SD) ESJ, p = .829. Error bars represent standard errors. Bottom graph shows description statistics and distributional properties based on raw data.*

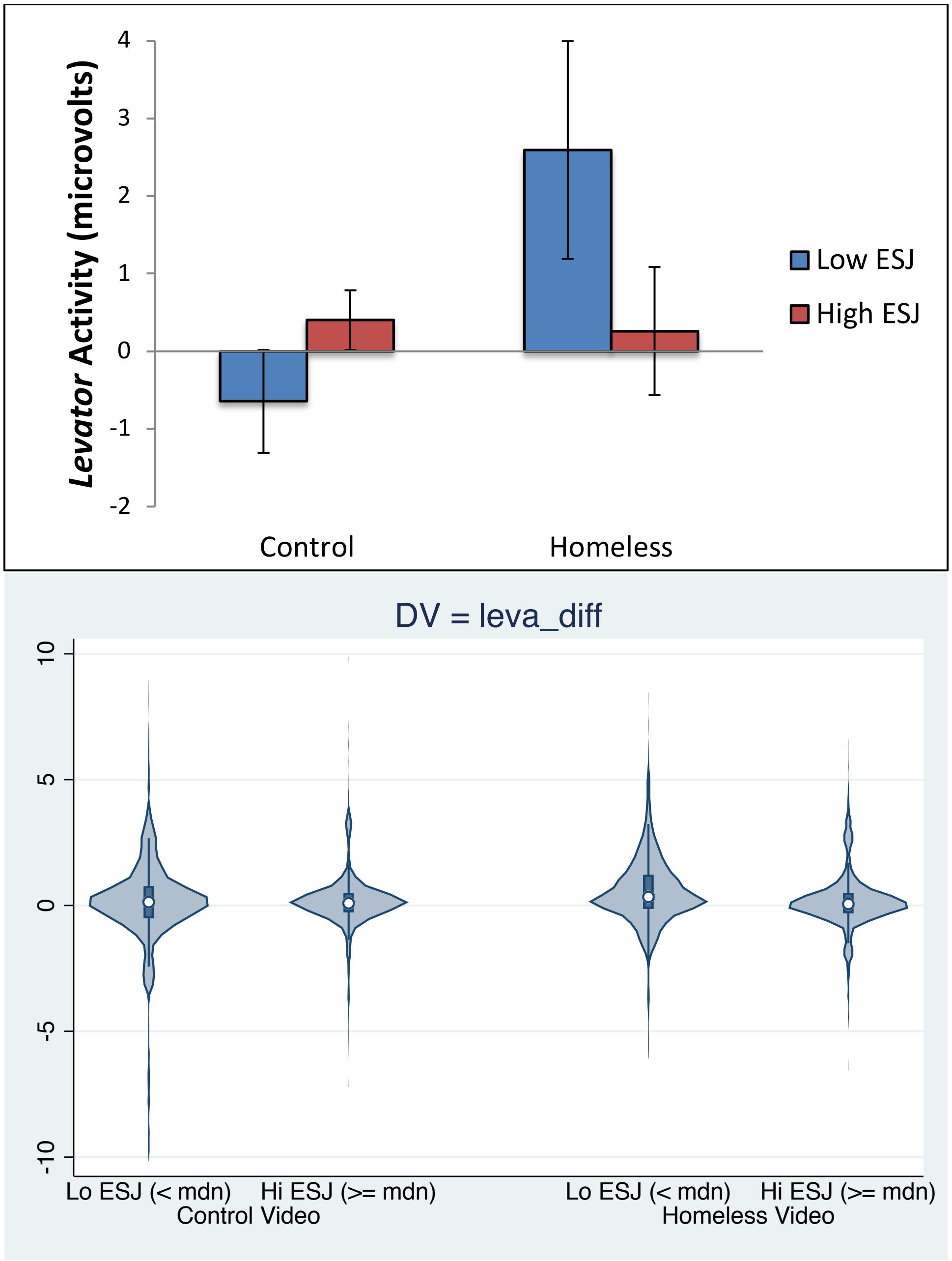

Supplementary Figure 32. *ESJ × Video Type interaction for levator activity in Study 3, p = .118. Simple effect of ESJ in control video condition, p = .177; simple effect of ESJ in homeless video condition, p = .101. Simple effect of video type (homeless vs. control) at low (-1 SD) ESJ, p = .112; simple effect of video type (homeless vs. control) at high (+1 SD) ESJ, p = .155. Error bars represent standard errors. Bottom graph shows description statistics and distributional properties based on raw data.*

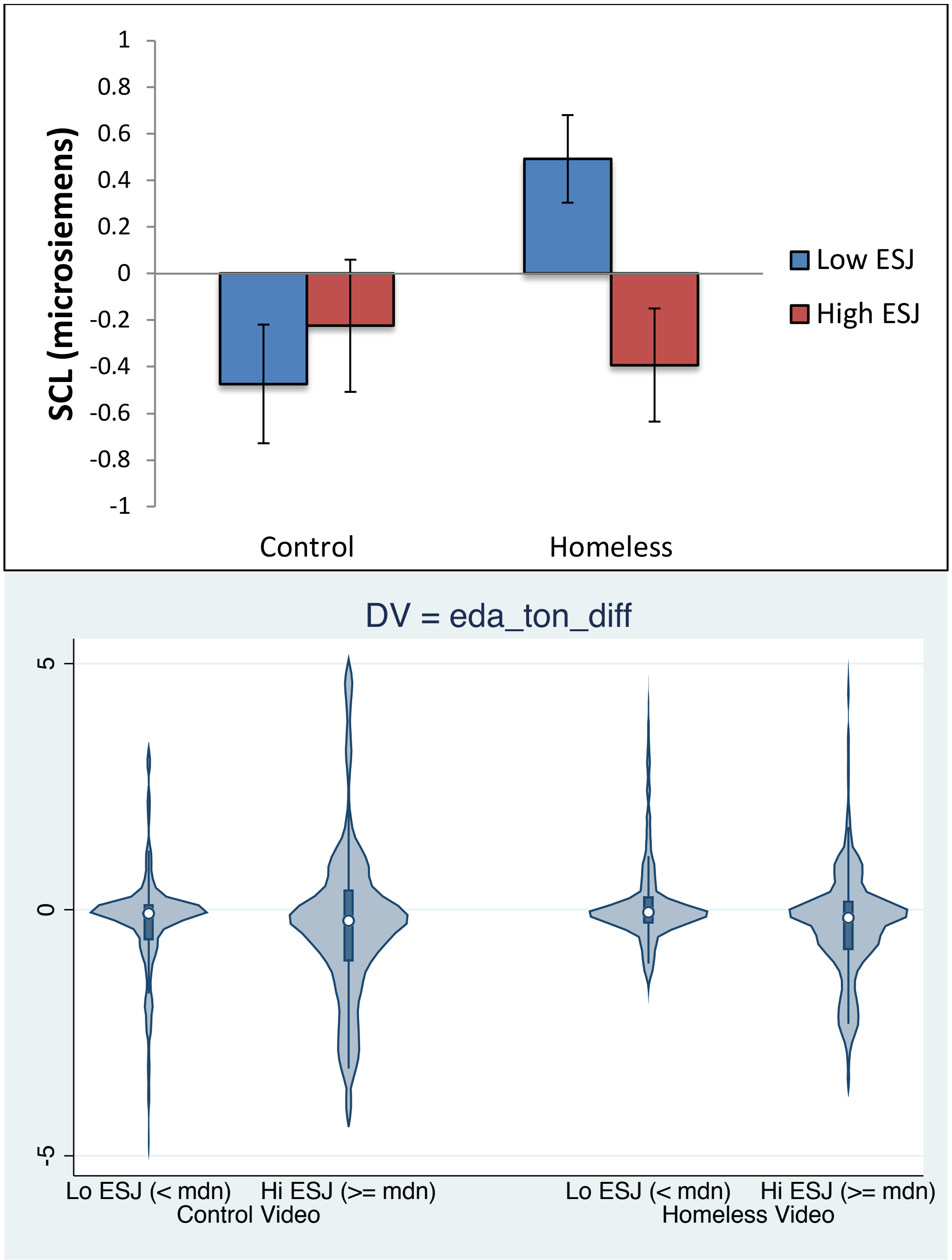

Supplementary Figure 33. *ESJ × Video Type interaction for SCL in Study 2, p = .027. Simple effect of ESJ in control video condition, p = .467; simple effect of ESJ in homeless video condition, p = .006. Simple effect of video type (homeless vs. control) at low (-1 SD) ESJ, p = .007; simple effect of video type (homeless vs. control) at high (+1 SD) ESJ, p = .548. Error bars represent standard errors. Bottom graph shows description statistics and distributional properties based on raw data.*

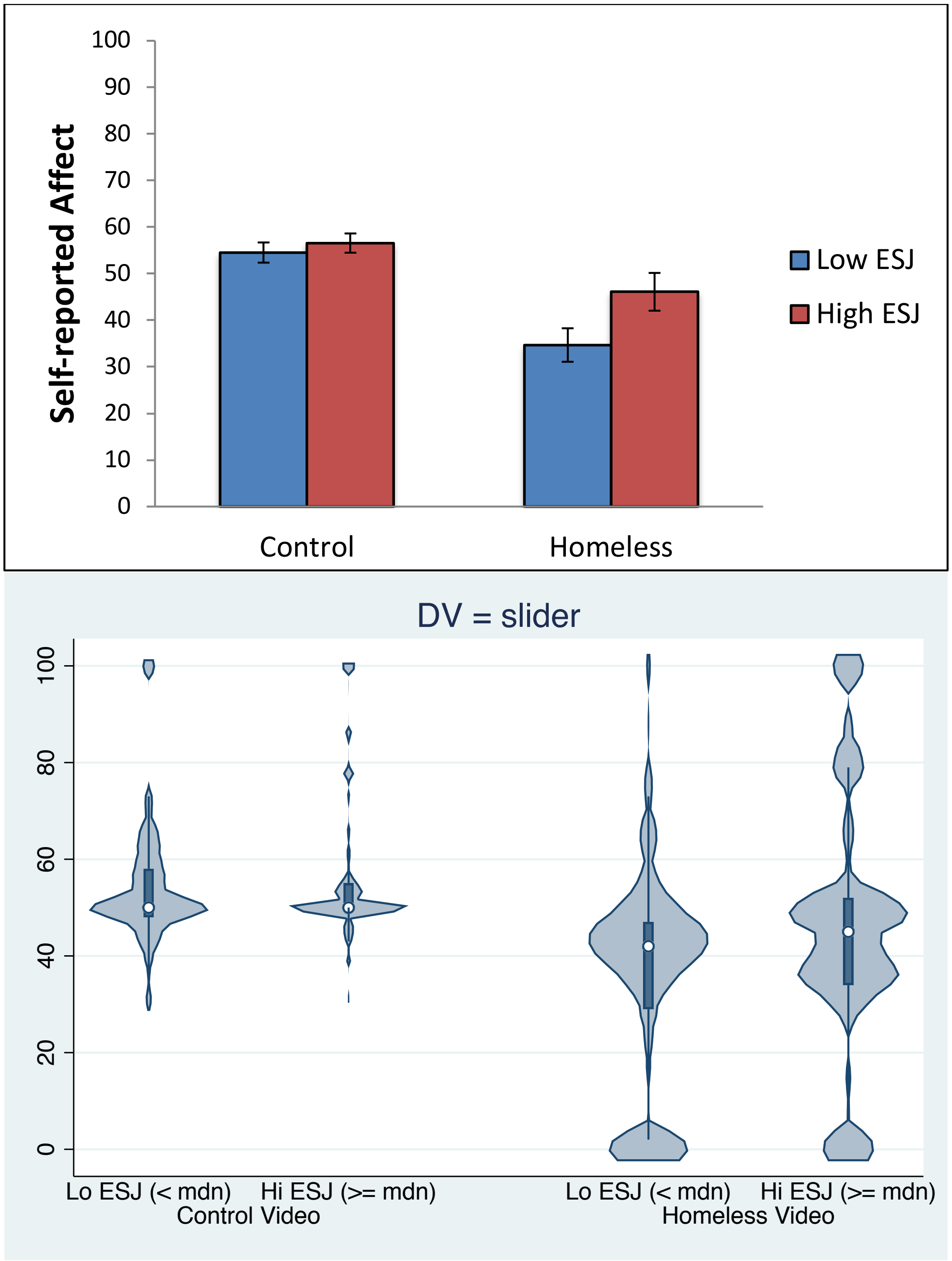

Supplementary Figure 34. *ESJ × Video Type interaction for positive affect in Study 3, p = .05. Simple effect of ESJ in control video condition, p = .409; simple effect of ESJ in homeless video condition, p = .012. Simple effect of video type (homeless vs. control) at low (-1 SD) ESJ, p < .001; simple effect of video type (homeless vs. control) at high (+1 SD) ESJ, p = .01. Error bars represent standard errors. Bottom graph shows description statistics and distributional properties based on raw data.*

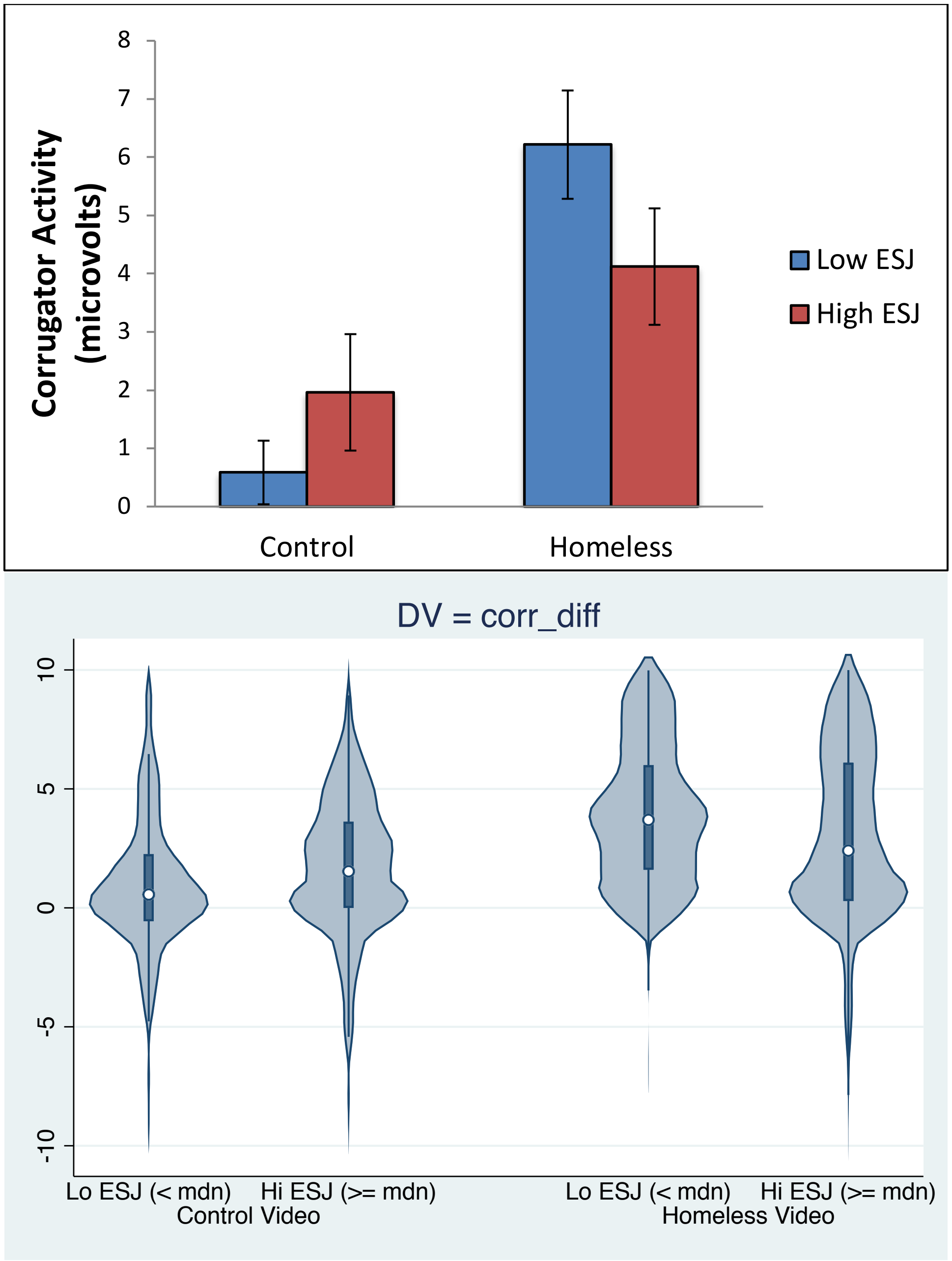

Supplementary Figure 35. *ESJ × Video Type interaction for corrugator activity in Study 4, p = .019. Simple effect of ESJ in control video condition, p = .108; simple effect of ESJ in homeless video condition, p = .138. Simple effect of video type (homeless vs. control) at low (-1 SD) ESJ, p < .001; simple effect of video type (homeless vs. control) at high (+1 SD) ESJ, p = .063. Error bars represent standard errors. Bottom graph shows description statistics and distributional properties based on raw data.*

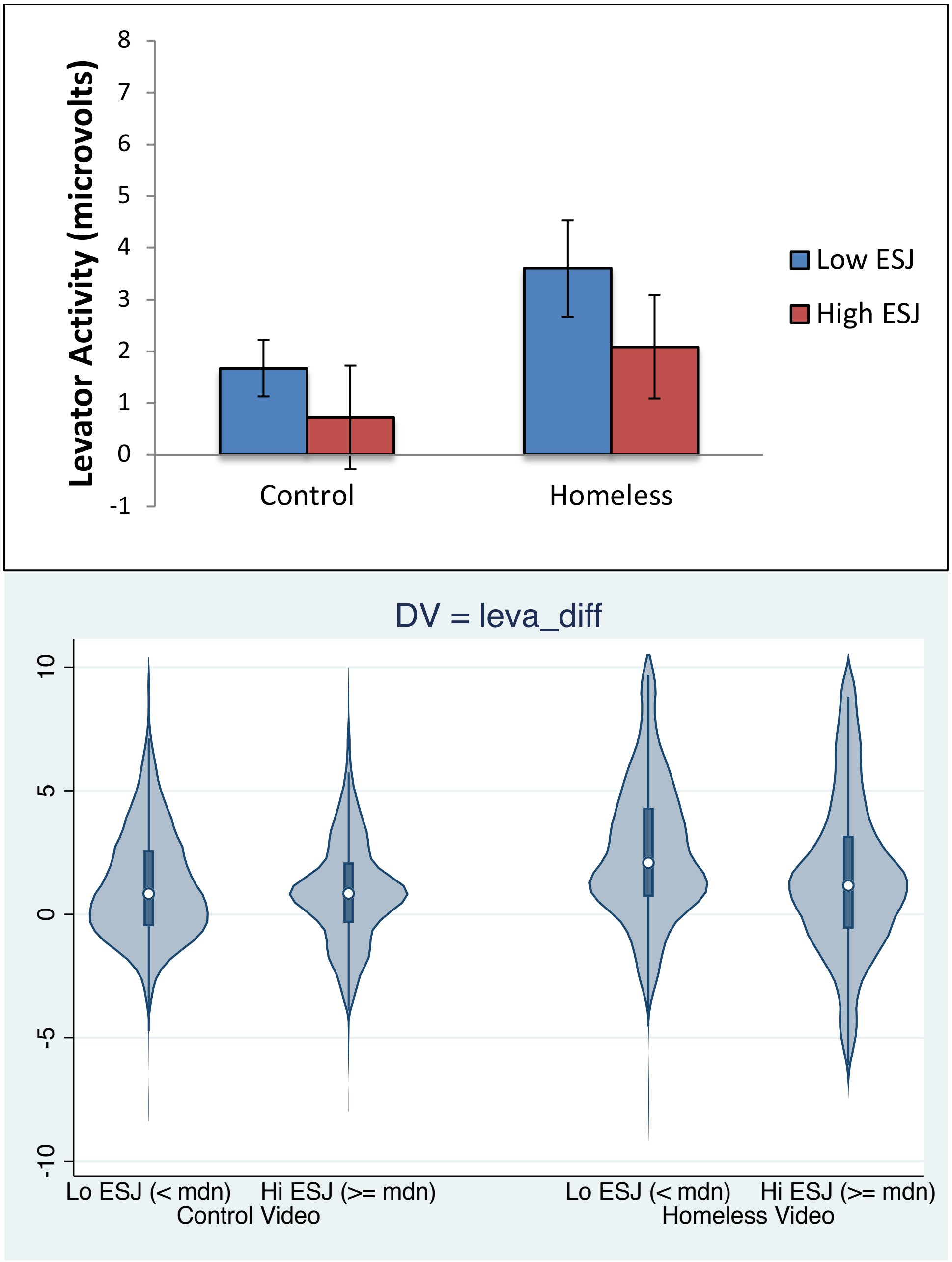

Supplementary Figure 36. *ESJ × Video Type interaction for levator activity in Study 4, p = .656. Simple effect of ESJ in control video condition, p = .085; simple effect of ESJ in homeless video condition, p = .194. Simple effect of video type (homeless vs. control) at low (-1 SD) ESJ, p = .041; simple effect of video type (homeless vs. control) at high (+1 SD) ESJ, p = .12. Error bars represent standard errors. Bottom graph shows description statistics and distributional properties based on raw data.*

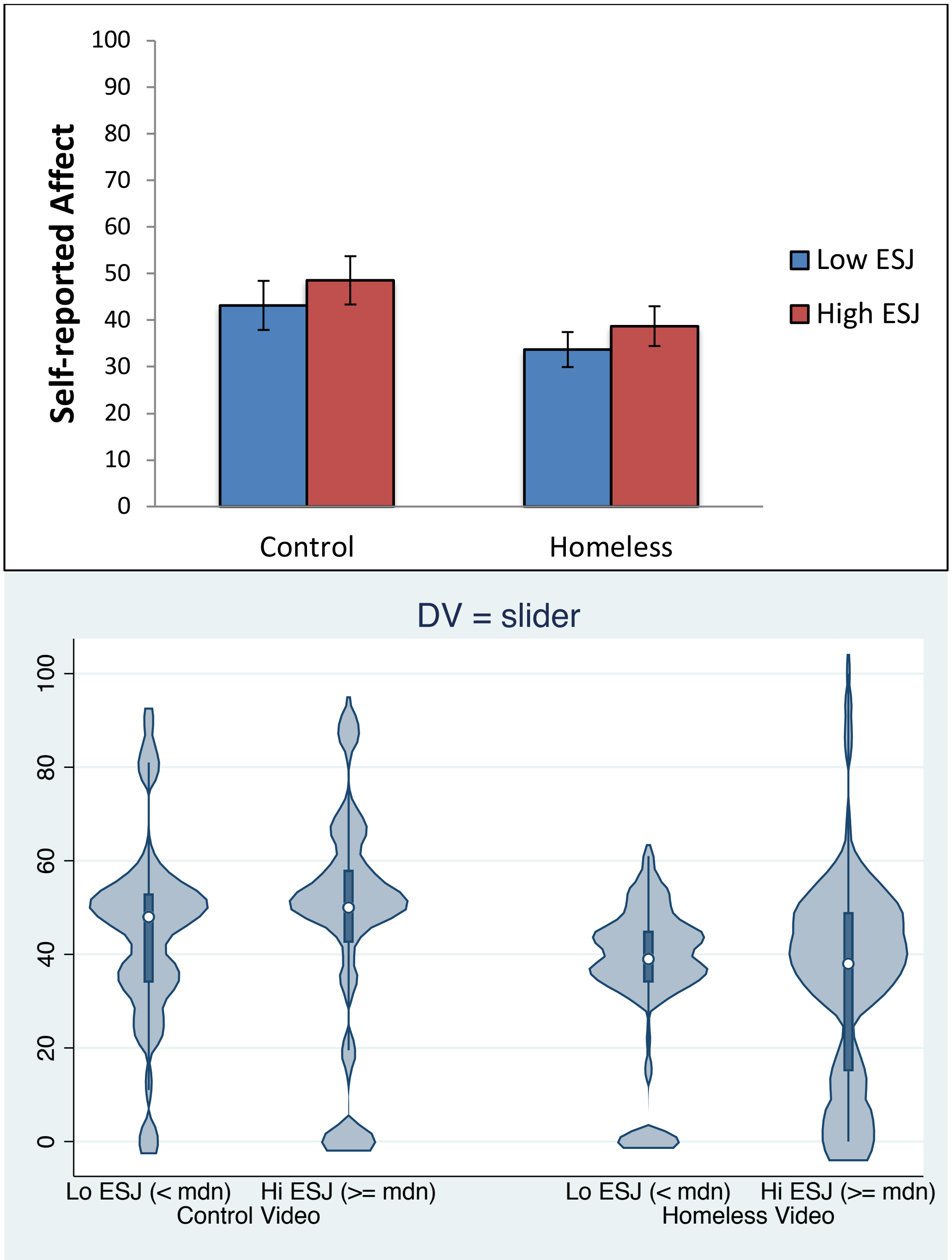

Supplementary Figure 37. *ESJ × Video Type interaction for positive affect in Study 4, p = .968. Simple effect of ESJ in control video condition, p = .492; simple effect of ESJ in homeless video condition, p = .391. Simple effect of video type (homeless vs. control) at low (-1 SD) ESJ, p = .035; simple effect of video type (homeless vs. control) at high (+1 SD) ESJ, p = .142. Error bars represent standard errors. Bottom graph shows description statistics and distributional properties based on raw data.*

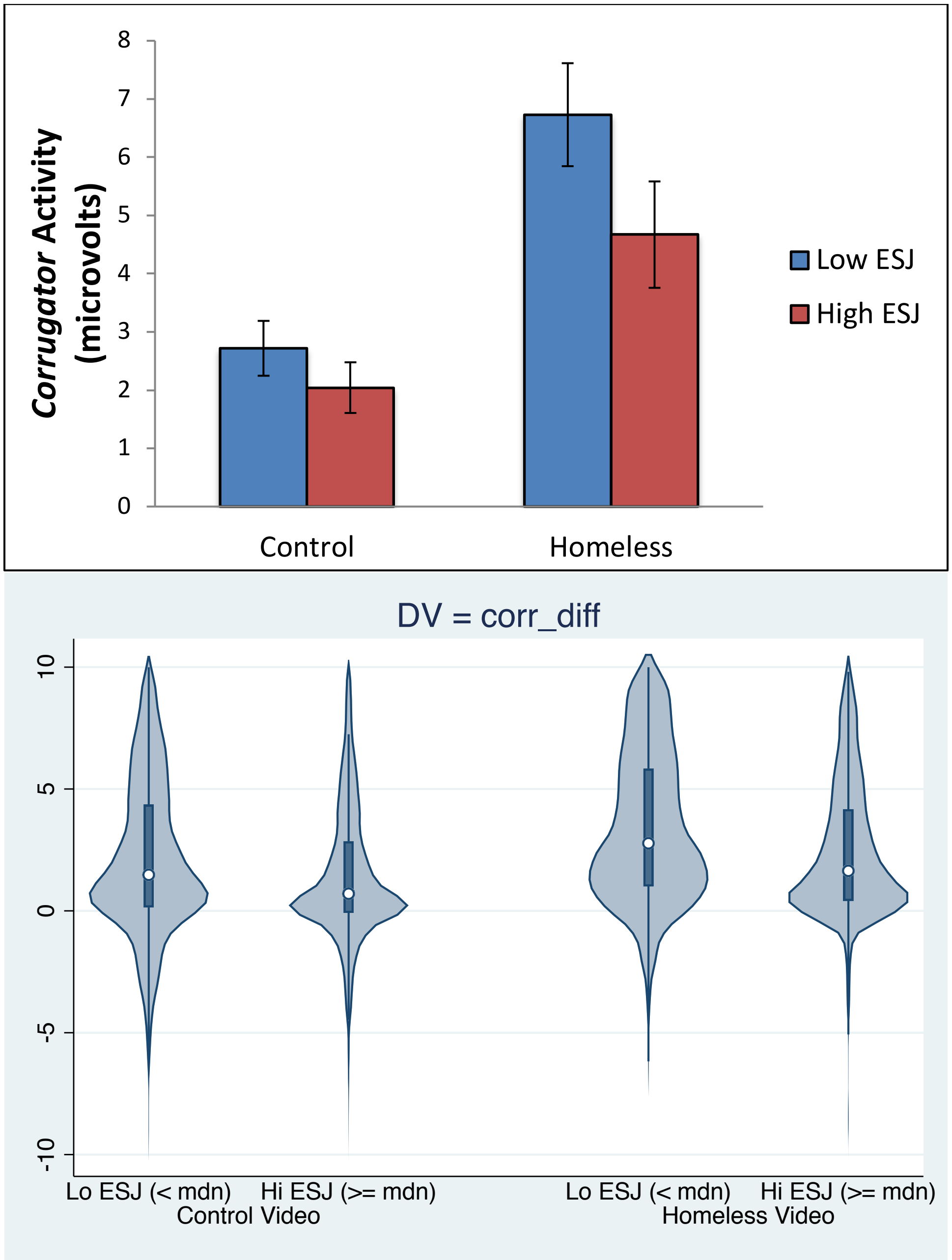

Supplementary Figure 38. *ESJ × Video Type interaction for corrugator activity in Study 5, p = .066. Simple effect of ESJ in control video condition, p = .734; simple effect of ESJ in homeless video condition, p = .149. Simple effect of video type (homeless vs. control) at low (-1 SD) ESJ, p < .001; simple effect of video type (homeless vs. control) at high (+1 SD) ESJ, p = .001. Error bars represent standard errors. Bottom graph shows description statistics and distributional properties based on raw data.*

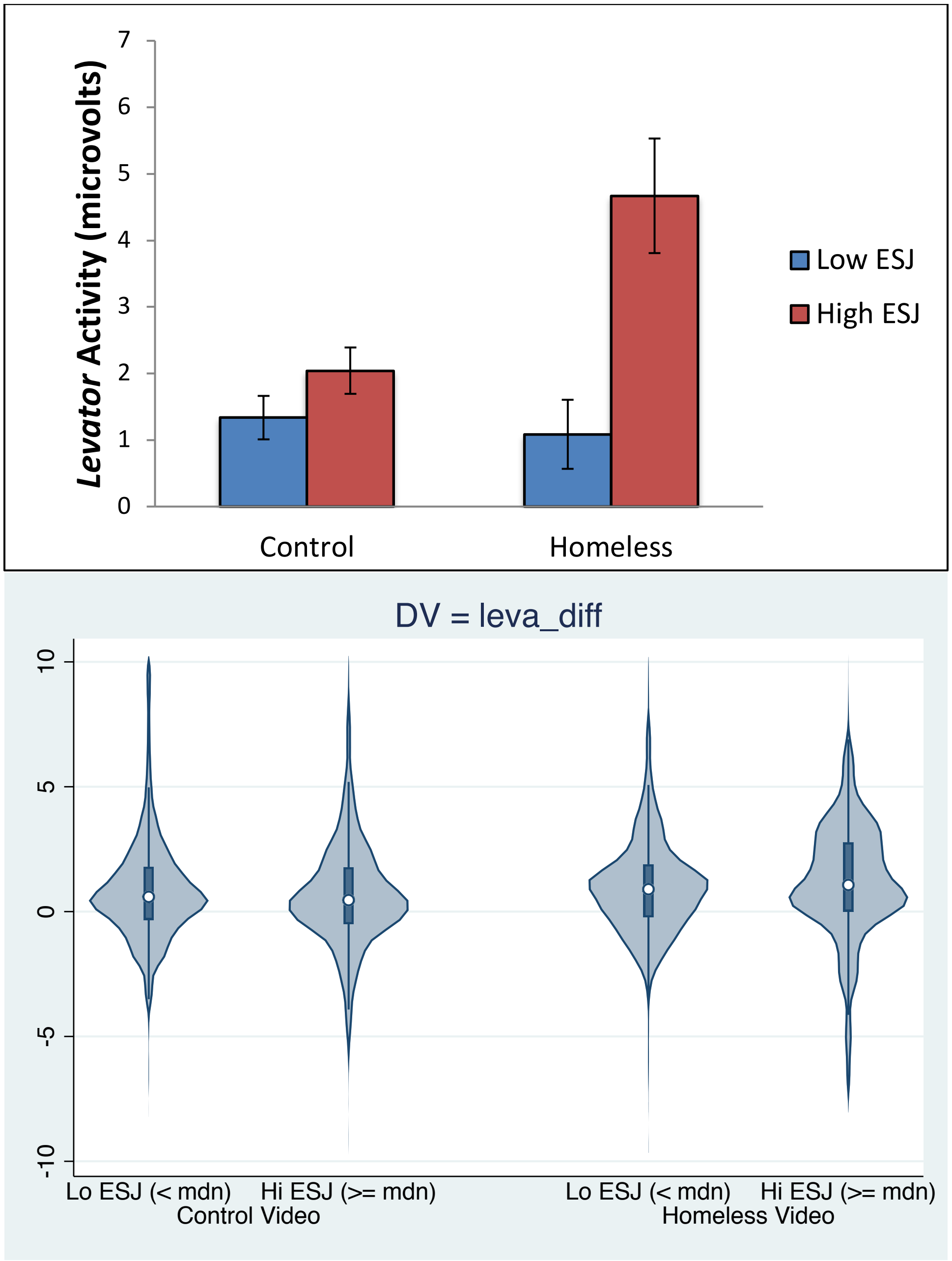

Supplementary Figure 39. *ESJ × Video Type interaction for levator activity in Study 5, p = .301. Simple effect of ESJ in control video condition, p = .20; simple effect of ESJ in homeless video condition, p = .46. Simple effect of video type (homeless vs. control) at low (-1 SD) ESJ, p = .714; simple effect of video type (homeless vs. control) at high (+1 SD) ESJ, p = .175. Error bars represent standard errors. Bottom graph shows description statistics and distributional properties based on raw data.*

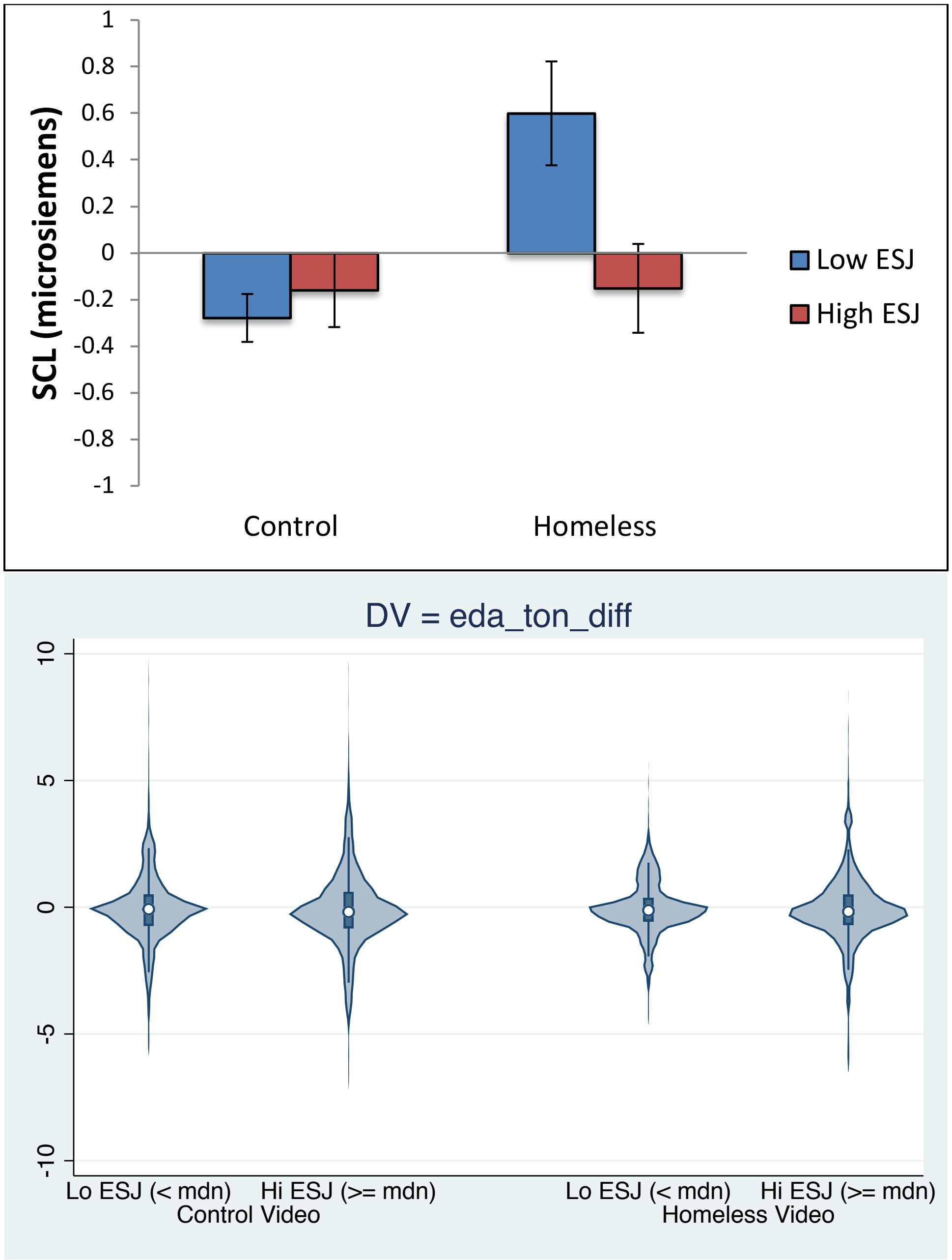

Supplementary Figure 40. *ESJ × Video Type interaction for SCL in Study 5, p = .034. Simple effect of ESJ in control video condition, p = .112; simple effect of ESJ in homeless video condition, p = .052. Simple effect of video type (homeless vs. control) at low (-1 SD) ESJ, p < .001; simple effect of video type (homeless vs. control) at high (+1 SD) ESJ, p = .973. Error bars represent standard errors. Bottom graph shows description statistics and distributional properties based on raw data.*

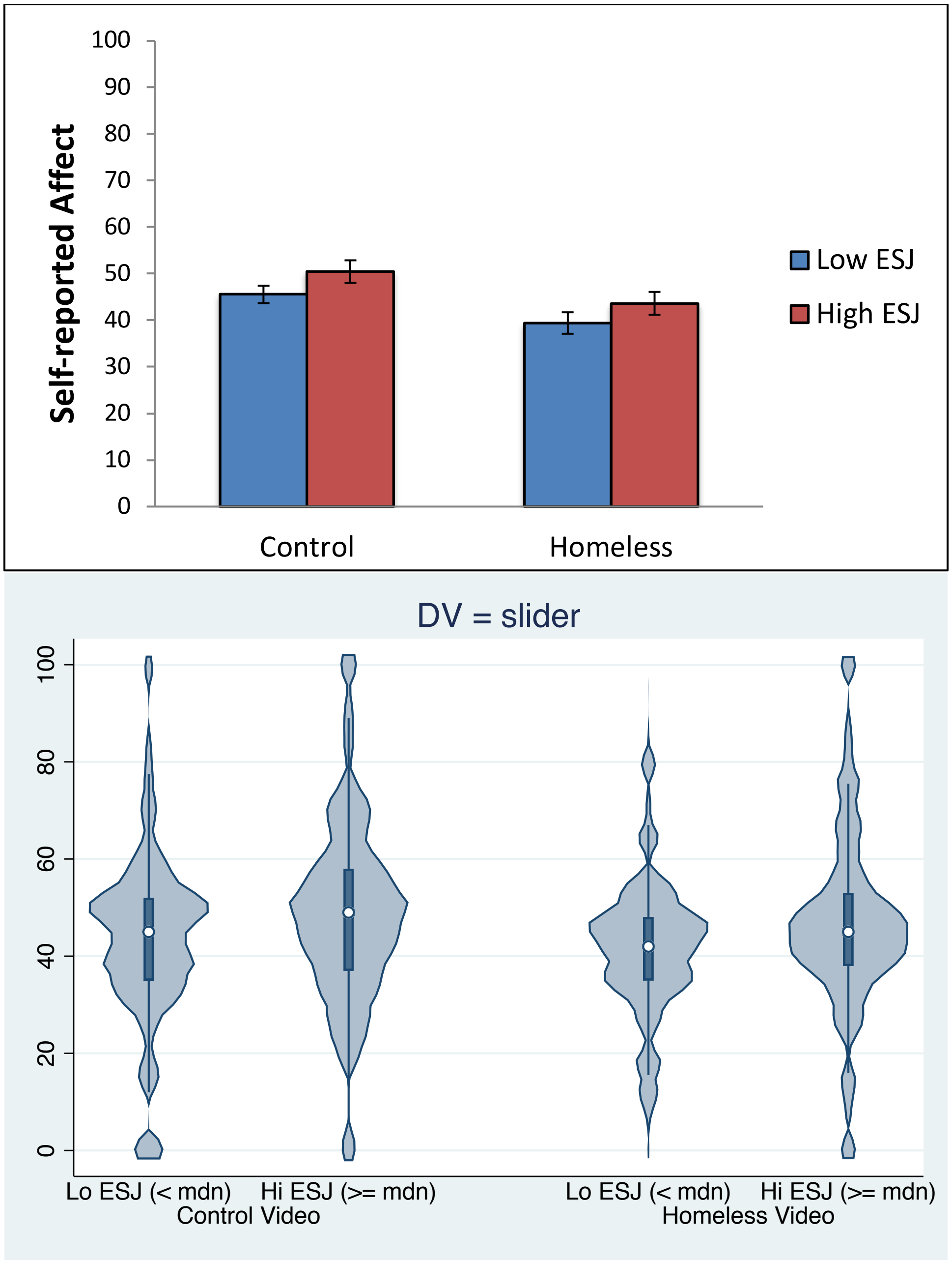

Supplementary Figure 41. *ESJ × Video Type interaction for positive affect in Study 5, p = .884. Simple effect of ESJ in control video condition, p = .133; simple effect of ESJ in homeless video condition, p = .208. Simple effect of video type (homeless vs. control) at low (-1 SD) ESJ, p = .006; simple effect of video type (homeless vs. control) at high (+1 SD) ESJ, p < .001. Error bars represent standard errors. Bottom graph shows description statistics and distributional properties based on raw data.*

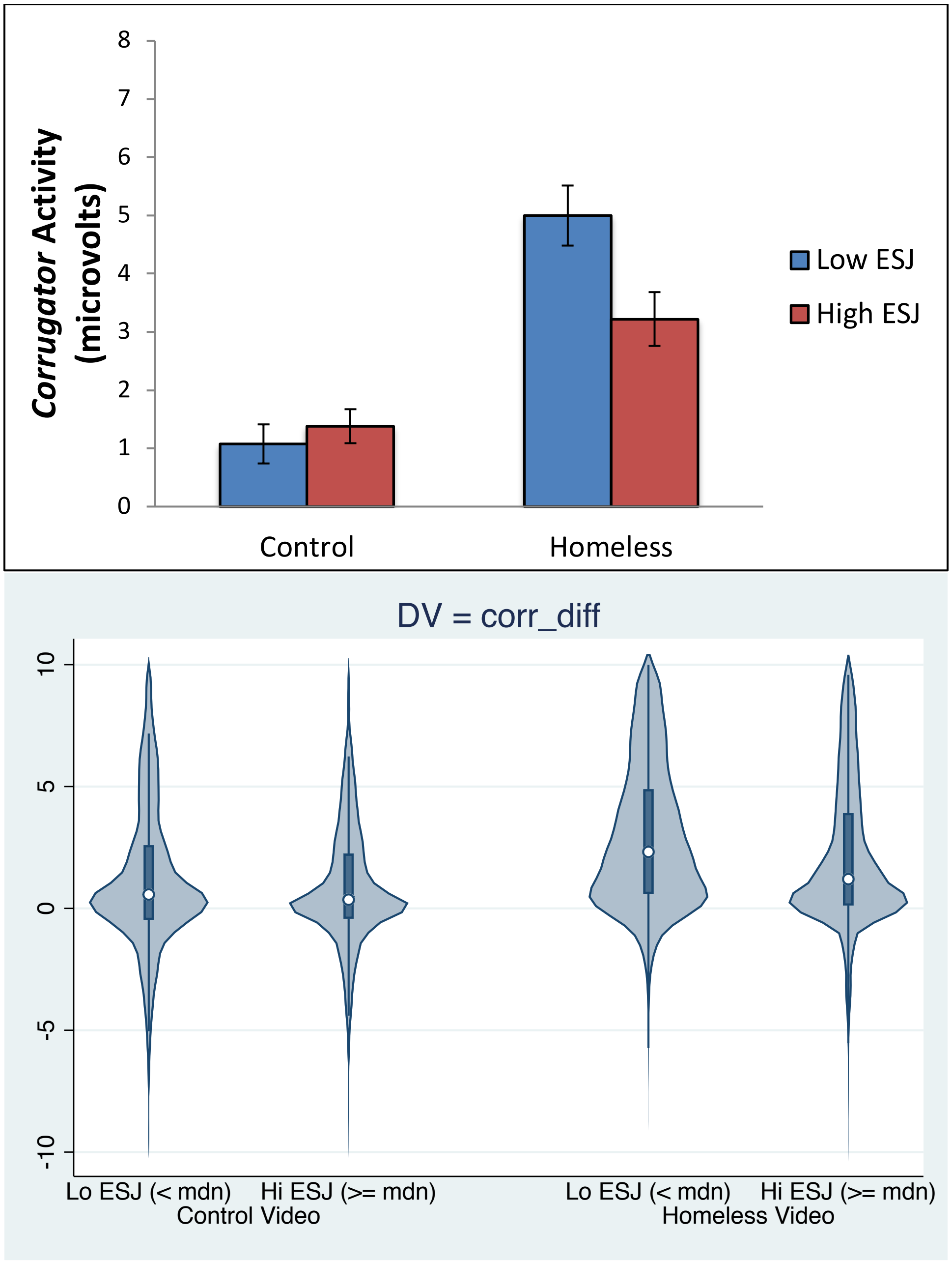

Supplementary Figure 42. *ESJ × Video Type interaction for corrugator activity in IDA, p = .001. Simple effect of ESJ in control video condition, p = .493; simple effect of ESJ in homeless video condition, p = .009. Simple effect of video type (homeless vs. control) at low (-1 SD) ESJ, p < .001; simple effect of video type (homeless vs. control) at high (+1 SD) ESJ, p < .001. Error bars represent standard errors. Bottom graph shows description statistics and distributional properties based on raw data.*

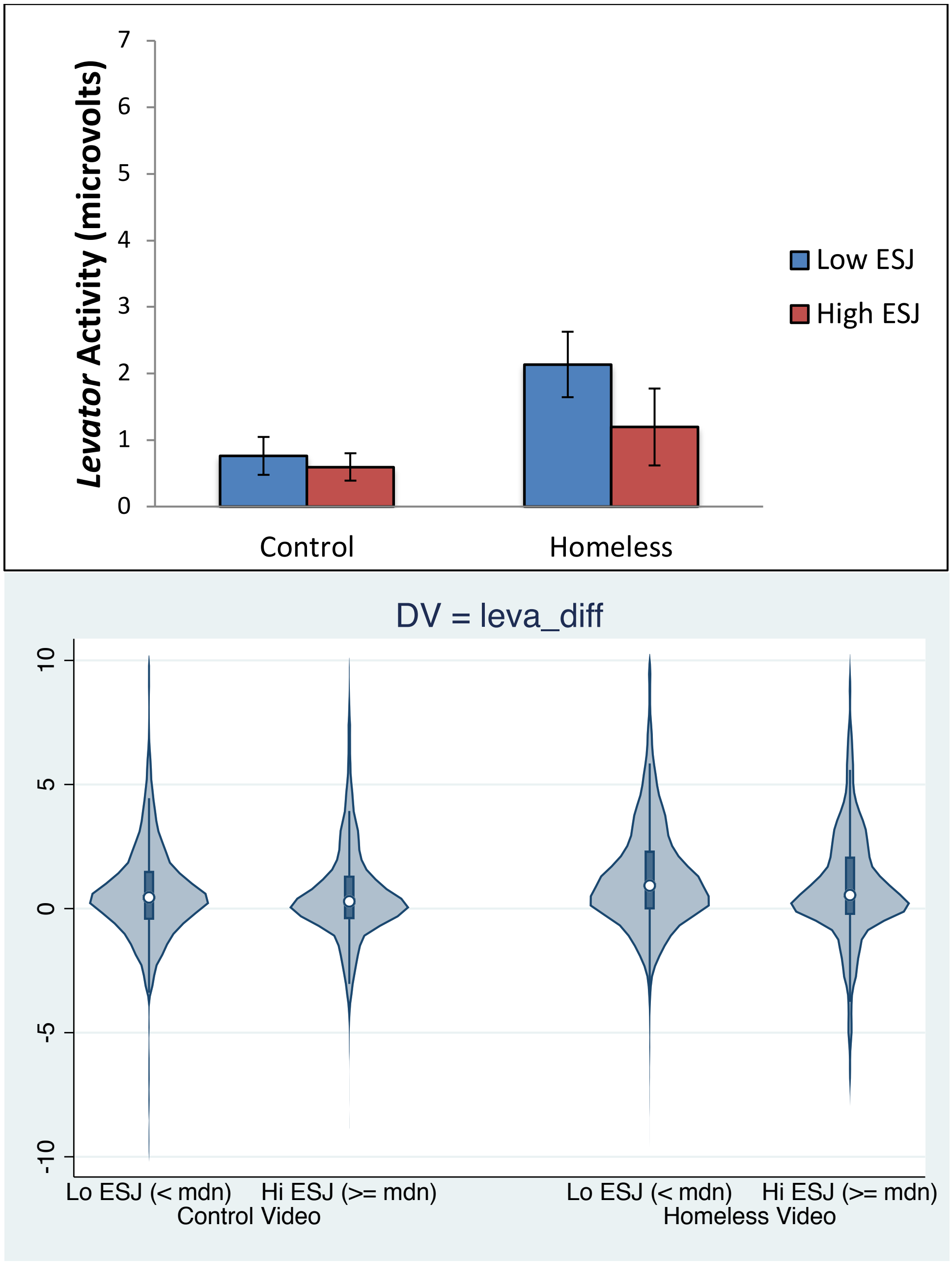

Supplementary Figure 43. *ESJ × Video Type interaction for levator activity in IDA, p = .268. Simple effect of ESJ in control video condition, p = .694; simple effect of ESJ in homeless video condition, p = .288. Simple effect of video type (homeless vs. control) at low (-1 SD) ESJ, p = .046; simple effect of video type (homeless vs. control) at high (+1 SD) ESJ, p = .363. Error bars represent standard errors. Bottom graph shows description statistics and distributional properties based on raw data.*

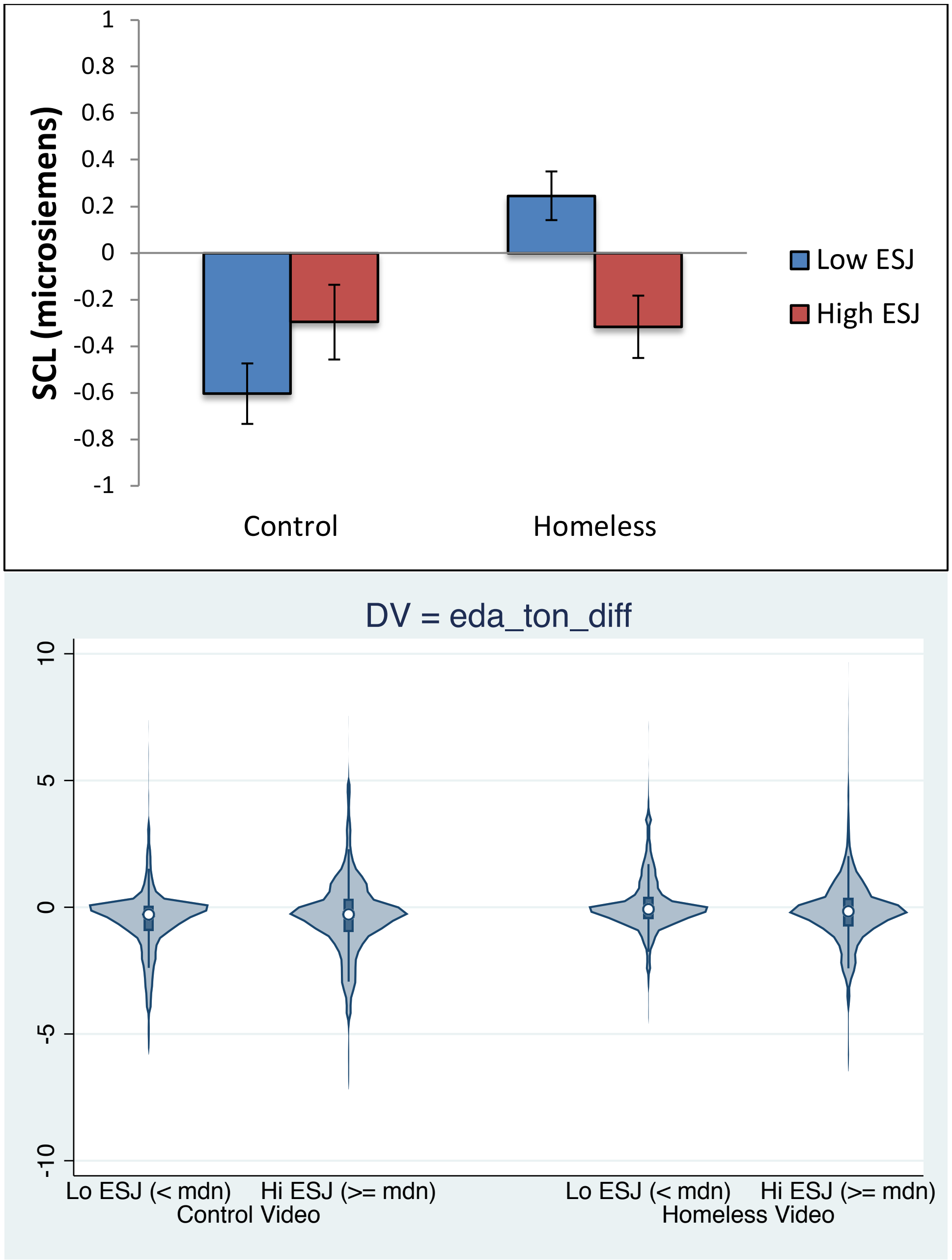

Supplementary Figure 44. *ESJ × Video Type interaction for SCL in IDA, p = .001. Simple effect of ESJ in control video condition, p = .108; simple effect of ESJ in homeless video condition, p = .001. Simple effect of video type (homeless vs. control) at low (-1 SD) ESJ, p < .001; simple effect of video type (homeless vs. control) at high (+1 SD) ESJ, p = .905. Error bars represent standard errors. Bottom graph shows description statistics and distributional properties based on raw data.*

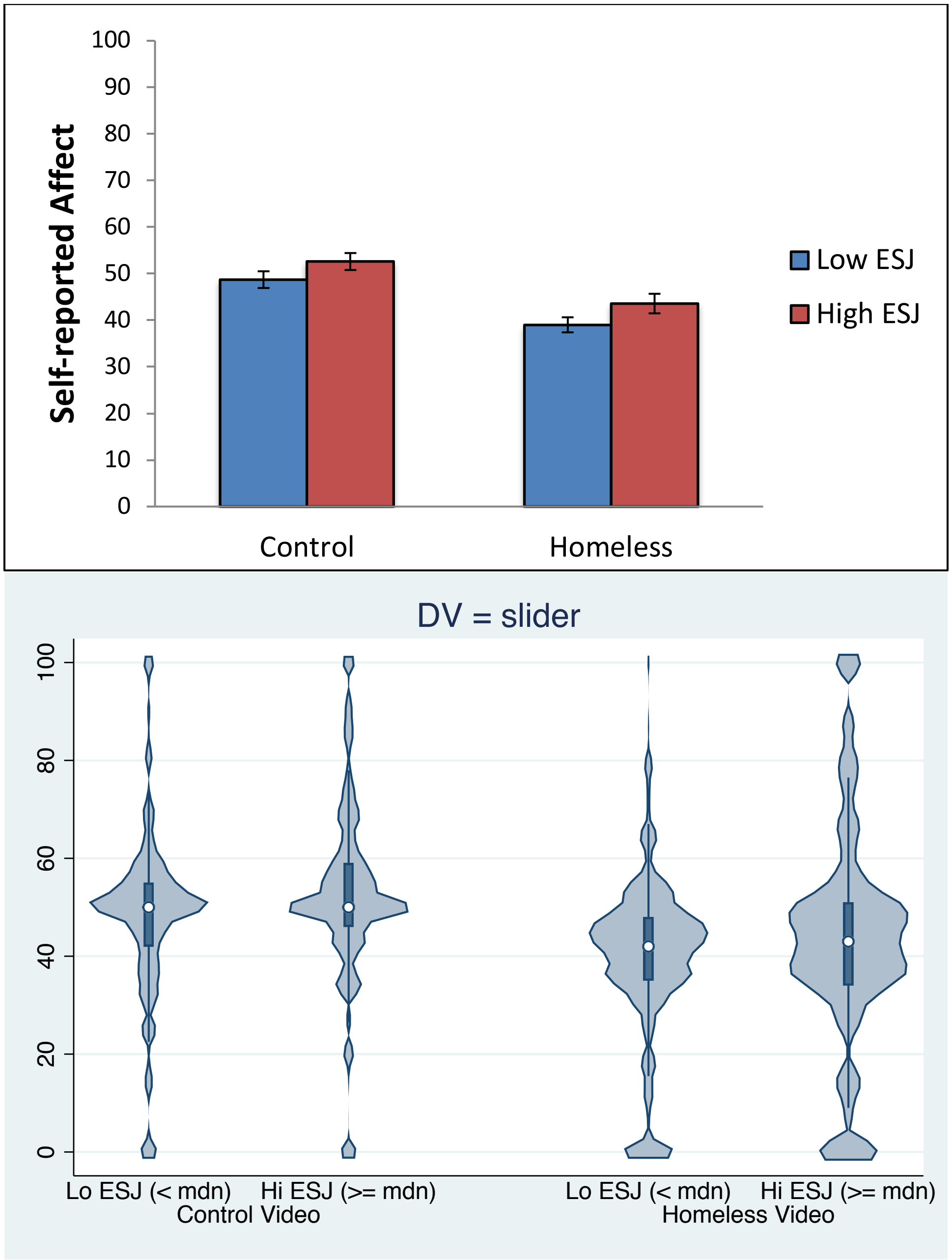

Supplementary Figure 45. *ESJ × Video Type interaction for positive affect in IDA, $p = .762$. Simple effect of ESJ in control video condition, $p = .105$; simple effect of ESJ in homeless video condition, $p = .058$. Simple effect of video type (homeless vs. control) at low (-1 SD) ESJ, $p < .001$; simple effect of video type (homeless vs. control) at high (+1 SD) ESJ, $p < .001$. Error bars represent standard errors. Bottom graph shows description statistics and distributional properties based on raw data.*

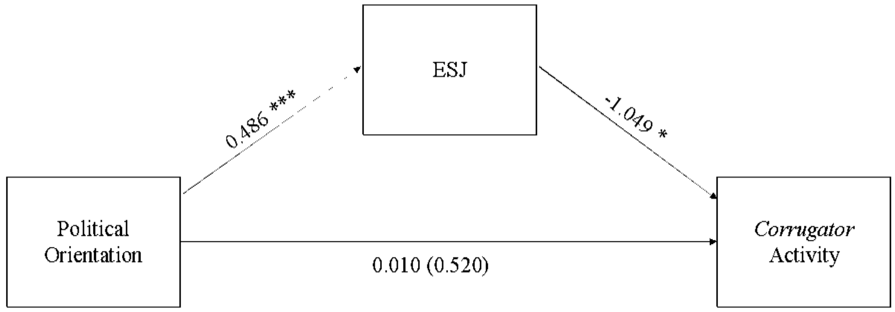

Supplementary Figure 46. *Regression coefficients for the relationship between political orientation and corrugator activity as mediated by Economic System Justification (ESJ) in IDA. The regression coefficient between political orientation and corrugator activity, adjusting for ESJ, is in parentheses.*
* $p < .05$, *** $p < .001$.

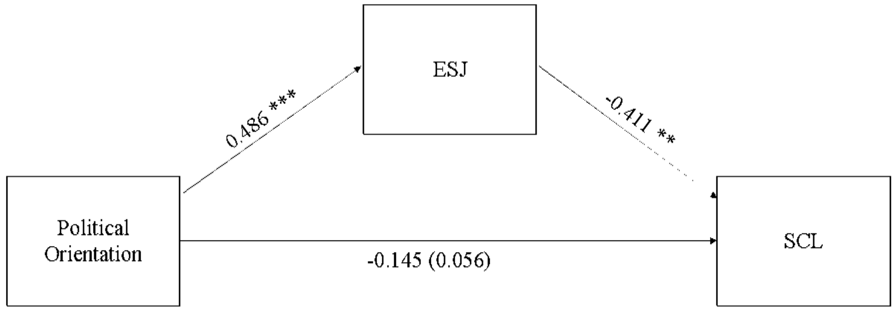

Supplementary Figure 47. *Regression coefficients for the relationship between political orientation and SCL as mediated by Economic System Justification (ESJ) in IDA. The regression coefficient between political orientation and SCL activity, adjusting for ESJ, is in parentheses.*
** $p < .01$, *** $p < .001$.

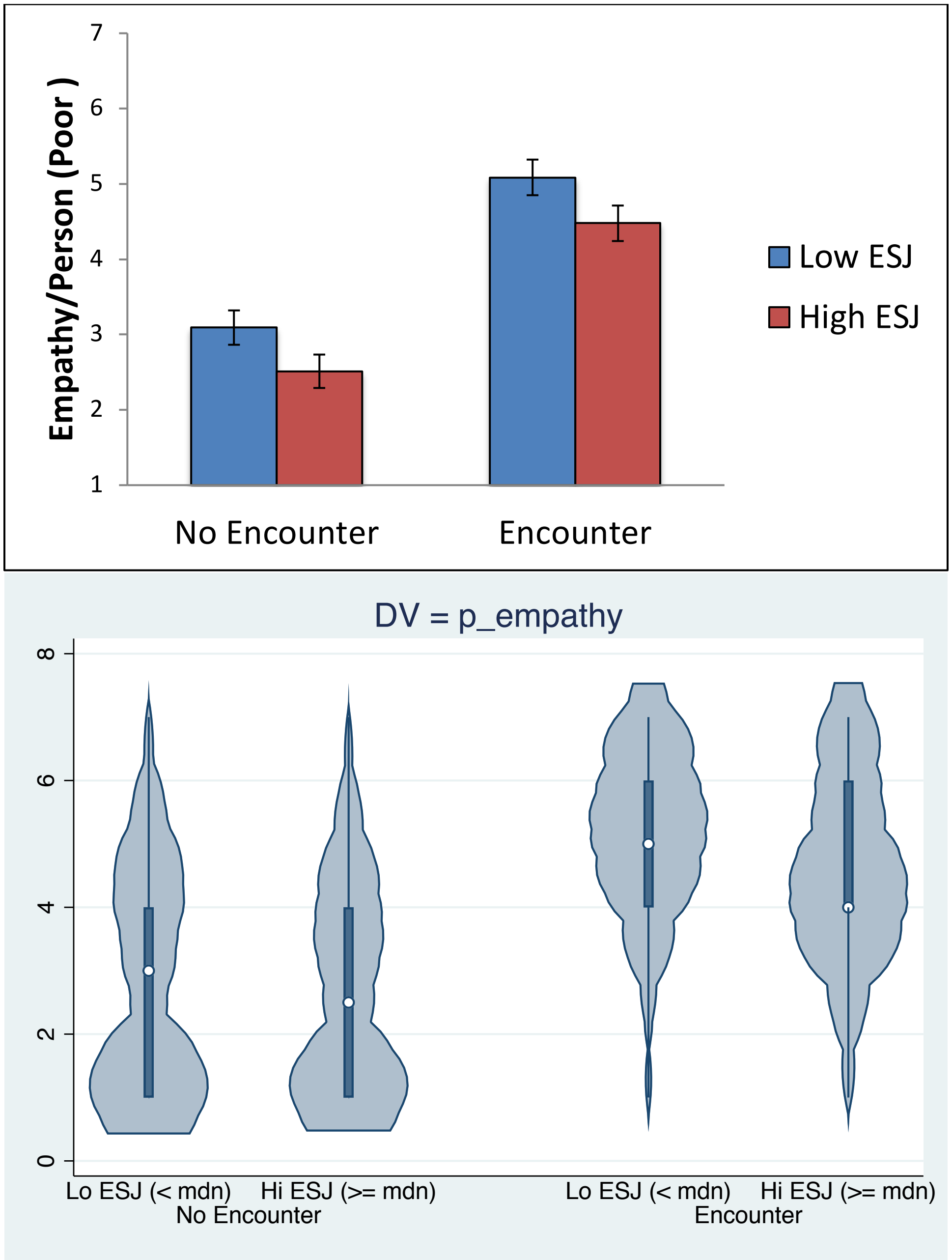

Supplementary Figure 48. *ESJ × Poor Encounter interaction for person-directed empathy in Study 6, p = .937. Simple effect of ESJ in no encounter condition, p = .074; simple effect of ESJ in encounter condition, p = .068. Simple effect of encounter (vs. no encounter) at low (-1 SD) ESJ, p < .001; simple effect of encounter (vs. no encounter) at high (+1 SD) ESJ, p < .001. Emotion was rated on a 1 (not at all) to 7 (extremely) scale. Error bars represent standard errors. Bottom graph shows description statistics and distributional properties based on raw data.*

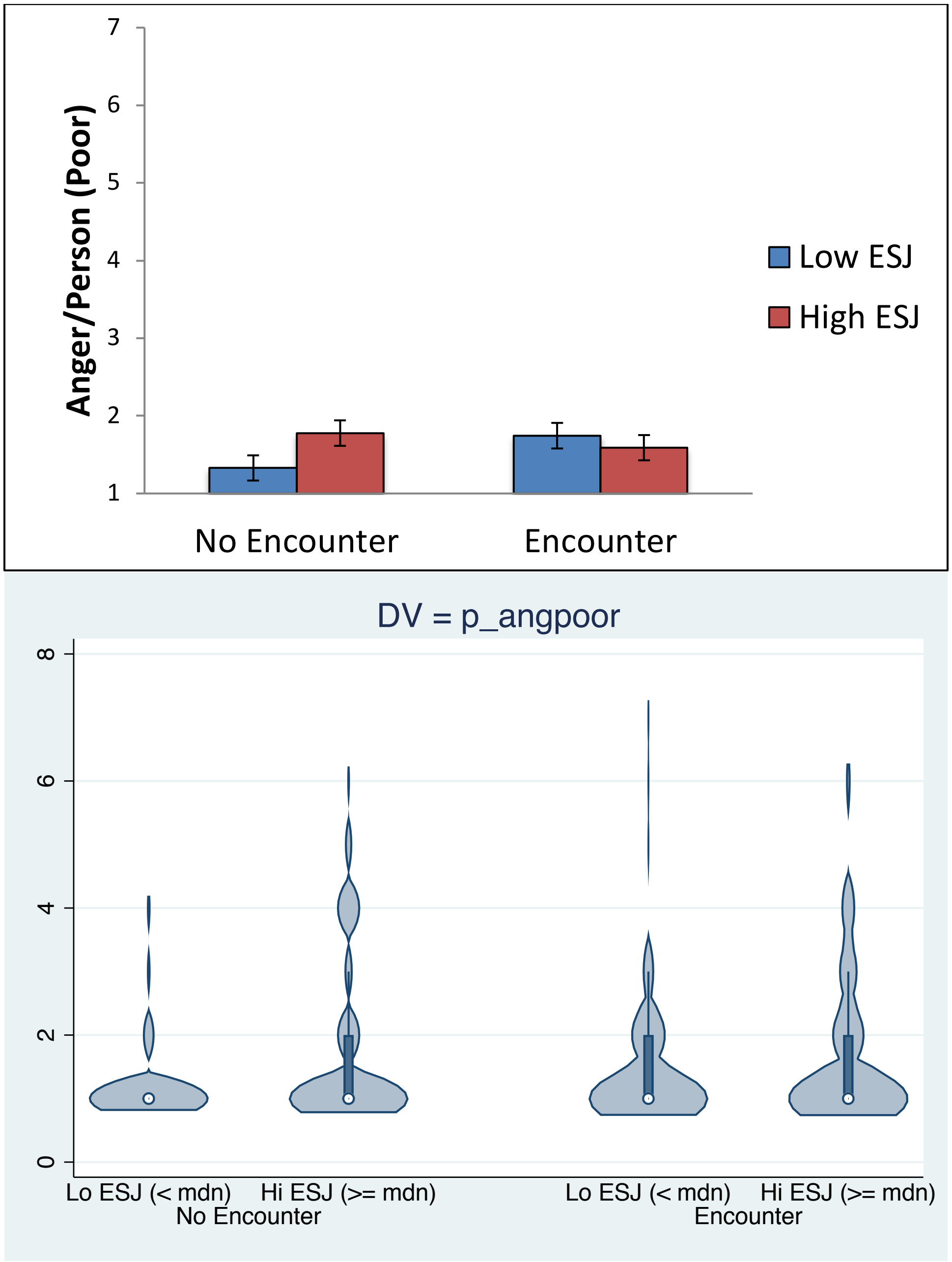

Supplementary Figure 49. *ESJ × Poor Encounter interaction for person-directed anger, p = .006. Simple effect of ESJ in no encounter condition, p = .052; simple effect of ESJ in encounter condition, p = .506. Simple effect of encounter (vs. no encounter) at low (-1 SD) ESJ, p = .007; simple effect of encounter (vs. no encounter) at high (+1 SD) ESJ, p = .24. Emotion was rated on a 1 (not at all) to 7 (extremely) scale. Error bars represent standard errors. Bottom graph shows description statistics and distributional properties based on raw data.*

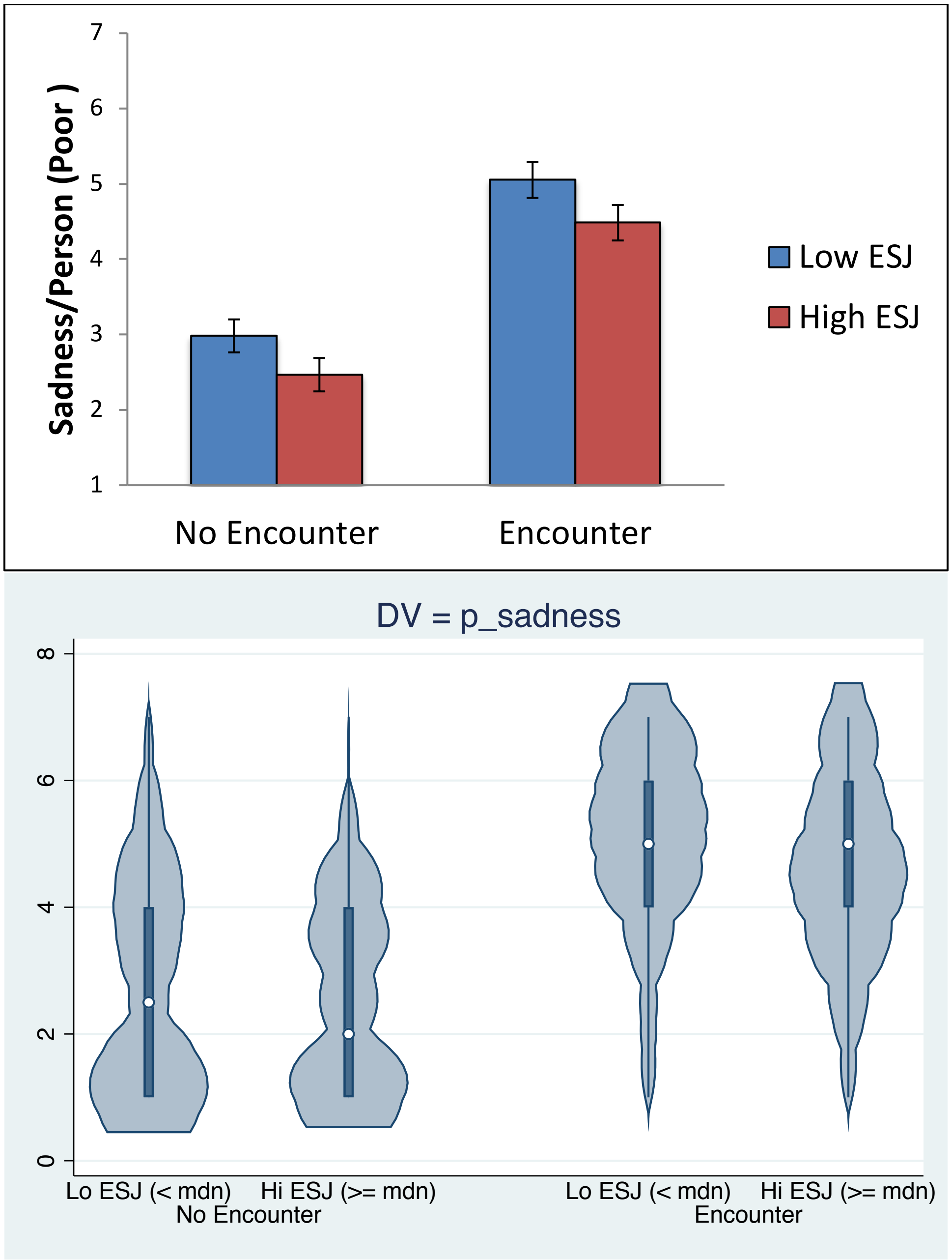

Supplementary Figure 50. *ESJ × Poor Encounter interaction for person-directed sadness in Study 6, p = .890. Simple effect of ESJ in no encounter condition, p = .099; simple effect of ESJ in encounter condition, p = .092. Simple effect of encounter (vs. no encounter) at low (-1 SD) ESJ, p < .001; simple effect of encounter (vs. no encounter) at high (+1 SD) ESJ, p < .001. Emotion was rated on a 1 (not at all) to 7 (extremely) scale. Error bars represent standard errors. Bottom graph shows description statistics and distributional properties based on raw data.*

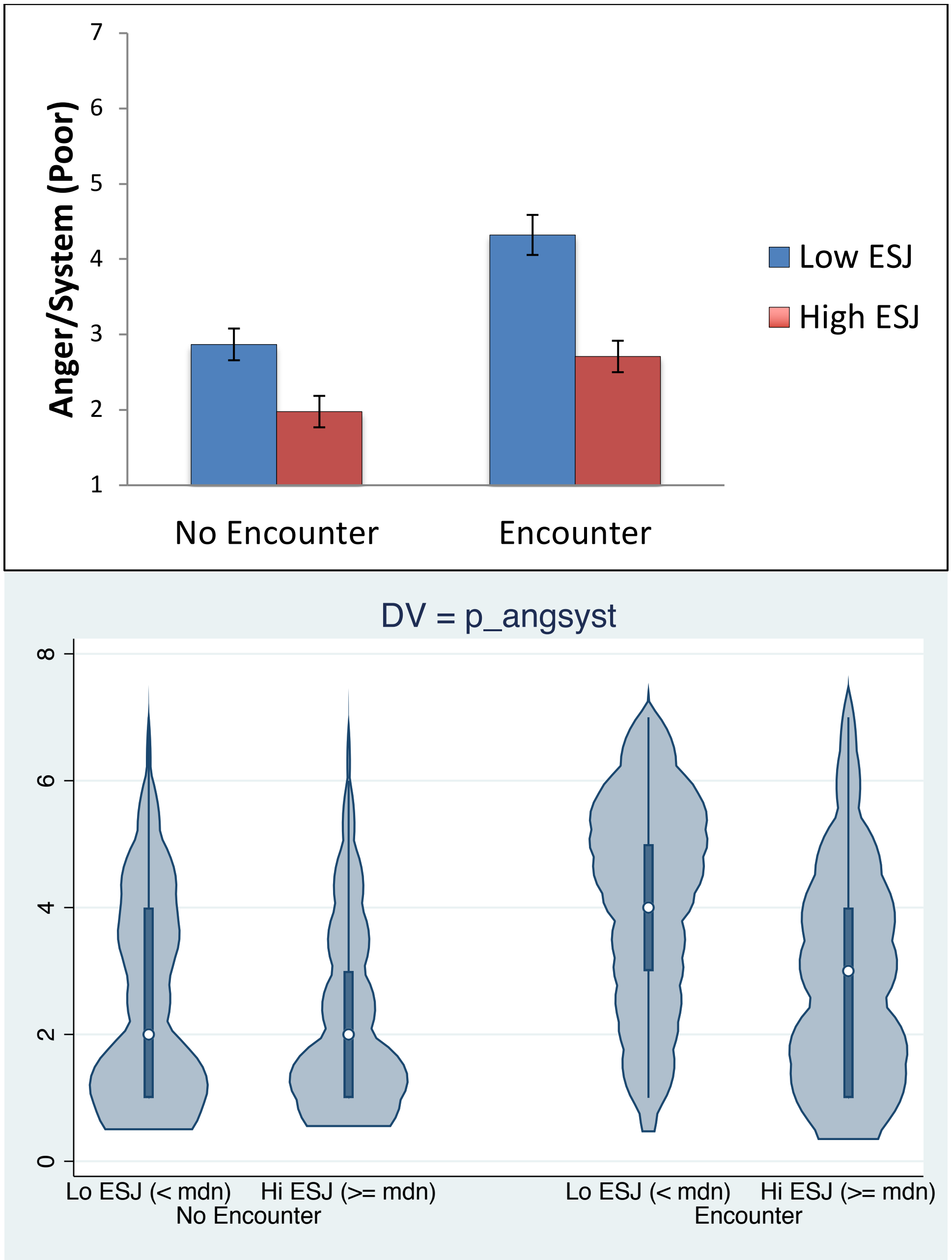

Supplementary Figure 51. *ESJ × Poor Encounter interaction for system-directed anger in Study 6, $p = .017$. Simple effect of ESJ in no encounter condition, $p = .003$; simple effect of ESJ in encounter condition, $p < .001$. Simple effect of encounter (vs. no encounter) at low (-1 SD) ESJ, $p < .001$; simple effect of encounter (vs. no encounter) at high (+1 SD) ESJ, $p = .001$. Emotion was rated on a 1 (not at all) to 7 (extremely) scale. Error bars represent standard errors. Bottom graph shows description statistics and distributional properties based on raw data.*

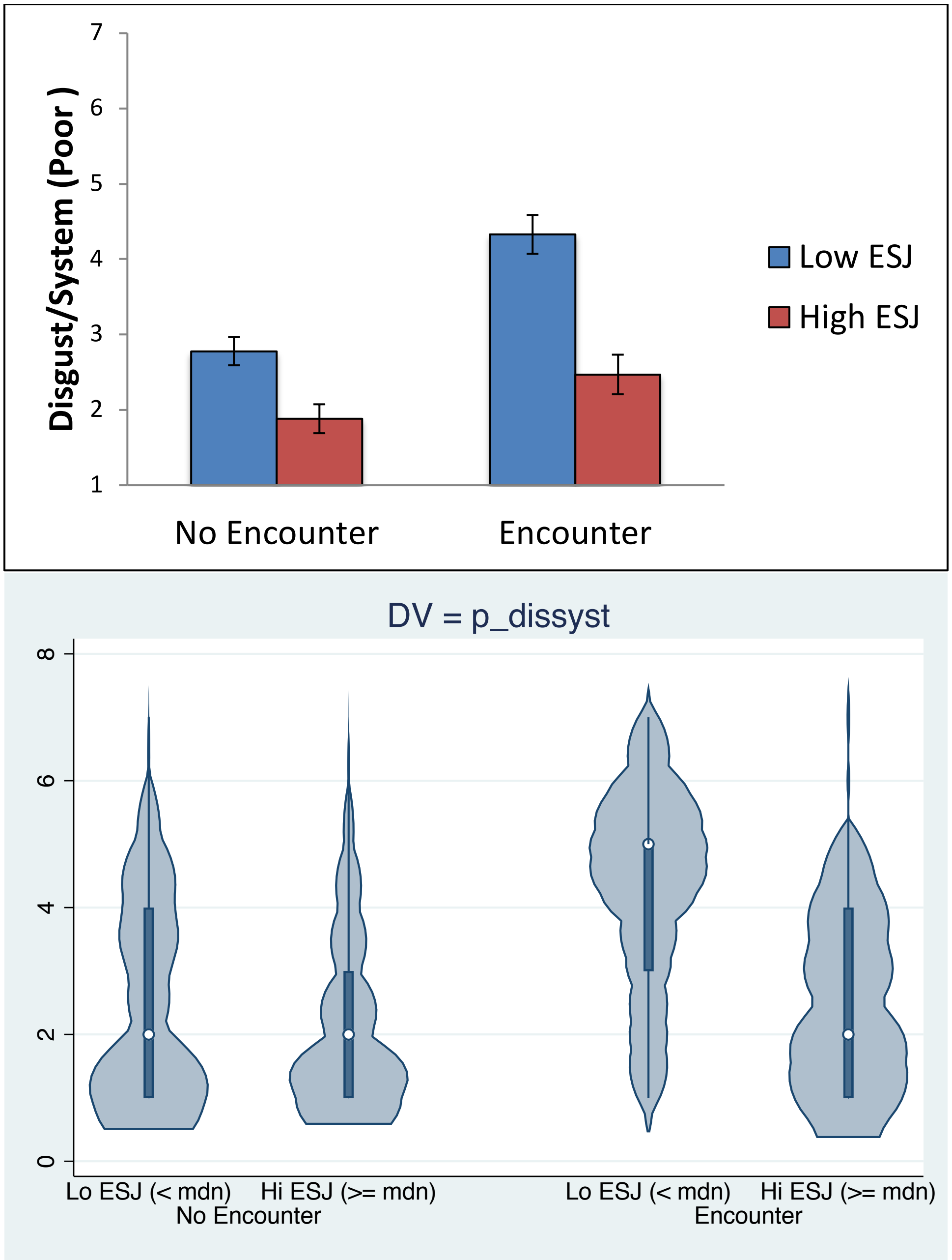

Supplementary Figure 52. *ESJ × Poor Encounter interaction for system-directed disgust in Study 6, p = .001. Simple effect of ESJ in no encounter condition, p = .001; simple effect of ESJ in encounter condition, p < .001. Simple effect of encounter (vs. no encounter) at low (-1 SD) ESJ, p < .001; simple effect of encounter (vs. no encounter) at high (+1 SD) ESJ, p = .006. Emotion was rated on a 1 (not at all) to 7 (extremely) scale. Error bars represent standard errors. Bottom graph shows description statistics and distributional properties based on raw data.*

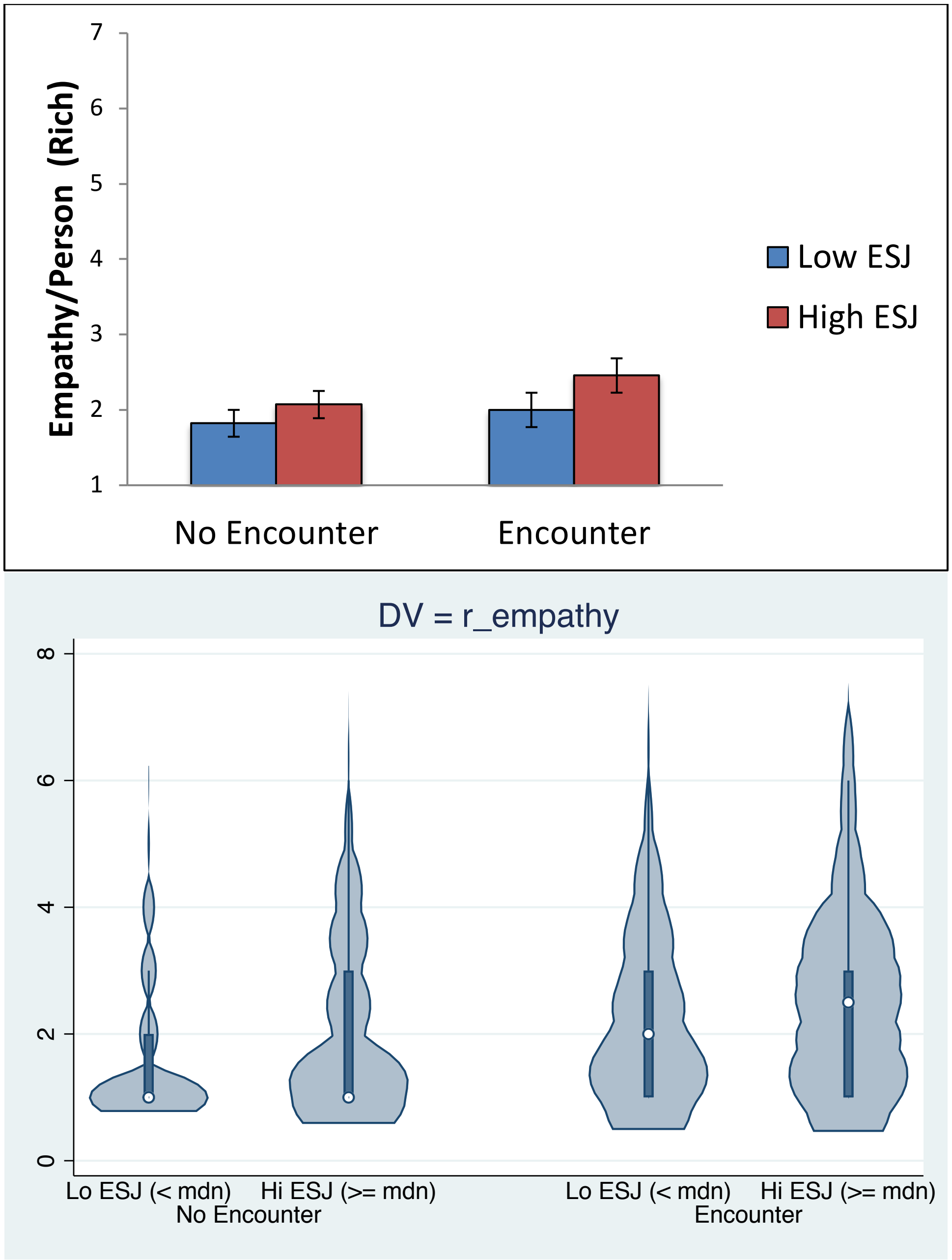

Supplementary Figure 53. *ESJ × Rich Encounter interaction for person-directed empathy in Study 6, p = .550. Simple effect of ESJ in no encounter condition, p = .329; simple effect of ESJ in encounter condition, p = .156. Simple effect of encounter (vs. no encounter) at low (-1 SD) ESJ, p = .473; simple effect of encounter (vs. no encounter) at high (+1 SD) ESJ, p = .122. Emotion was rated on a 1 (not at all) to 7 (extremely) scale. Error bars represent standard errors. Bottom graph shows description statistics and distributional properties based on raw data.*

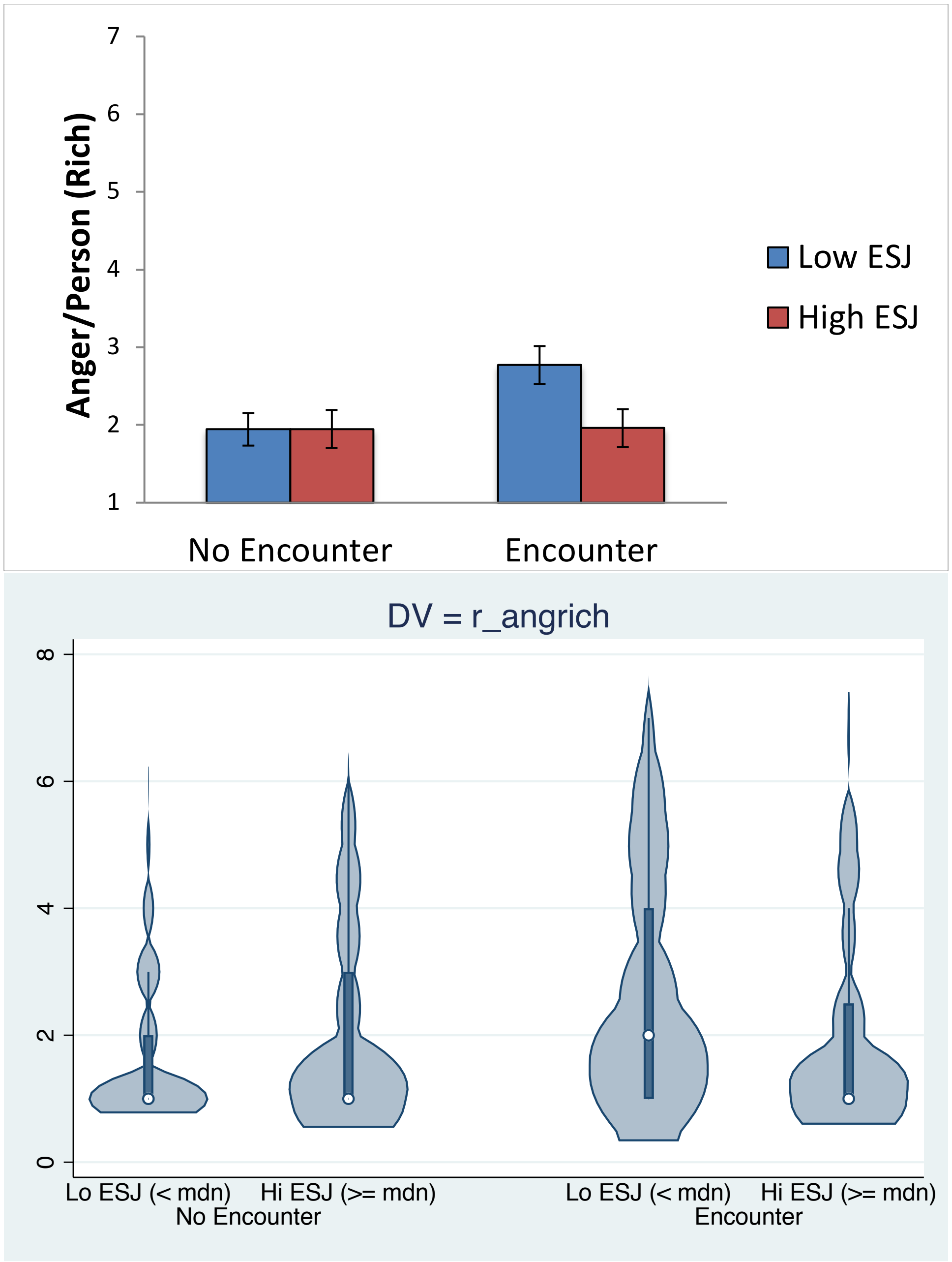

Supplementary Figure 54. *ESJ × Rich Encounter interaction for person-directed anger in Study 6, p = .021. Simple effect of ESJ in no encounter condition, p = .993; simple effect of ESJ in encounter condition, p = .019. Simple effect of encounter (vs. no encounter) at low (-1 SD) ESJ, p = .001; simple effect of encounter (vs. no encounter) at high (+1 SD) ESJ, p = .968. Emotion was rated on a 1 (not at all) to 7 (extremely) scale. Error bars represent standard errors. Bottom graph shows description statistics and distributional properties based on raw data.*

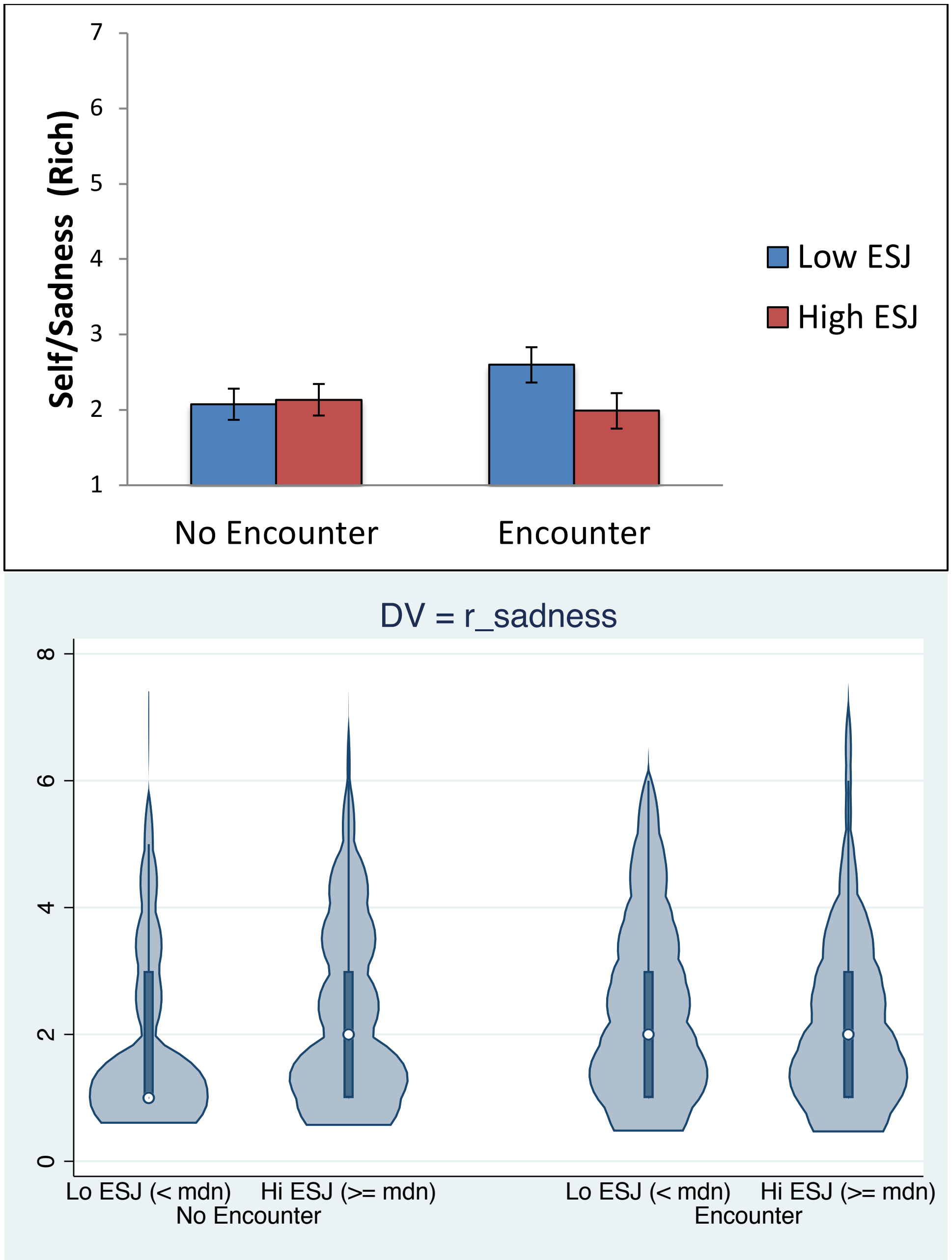

Supplementary Figure 55. *ESJ × Rich Encounter interaction for self-directed sadness in Study 6, p = .022. Simple effect of ESJ in no encounter condition, p = .84; simple effect of ESJ in encounter condition, p = .067. Simple effect of encounter (vs. no encounter) at low (-1 SD) ESJ, p = .012; simple effect of encounter (vs. no encounter) at high (+1 SD) ESJ, p = 485. Emotion was rated on a 1 (not at all) to 7 (extremely) scale. Error bars represent standard errors. Bottom graph shows description statistics and distributional properties based on raw data.*

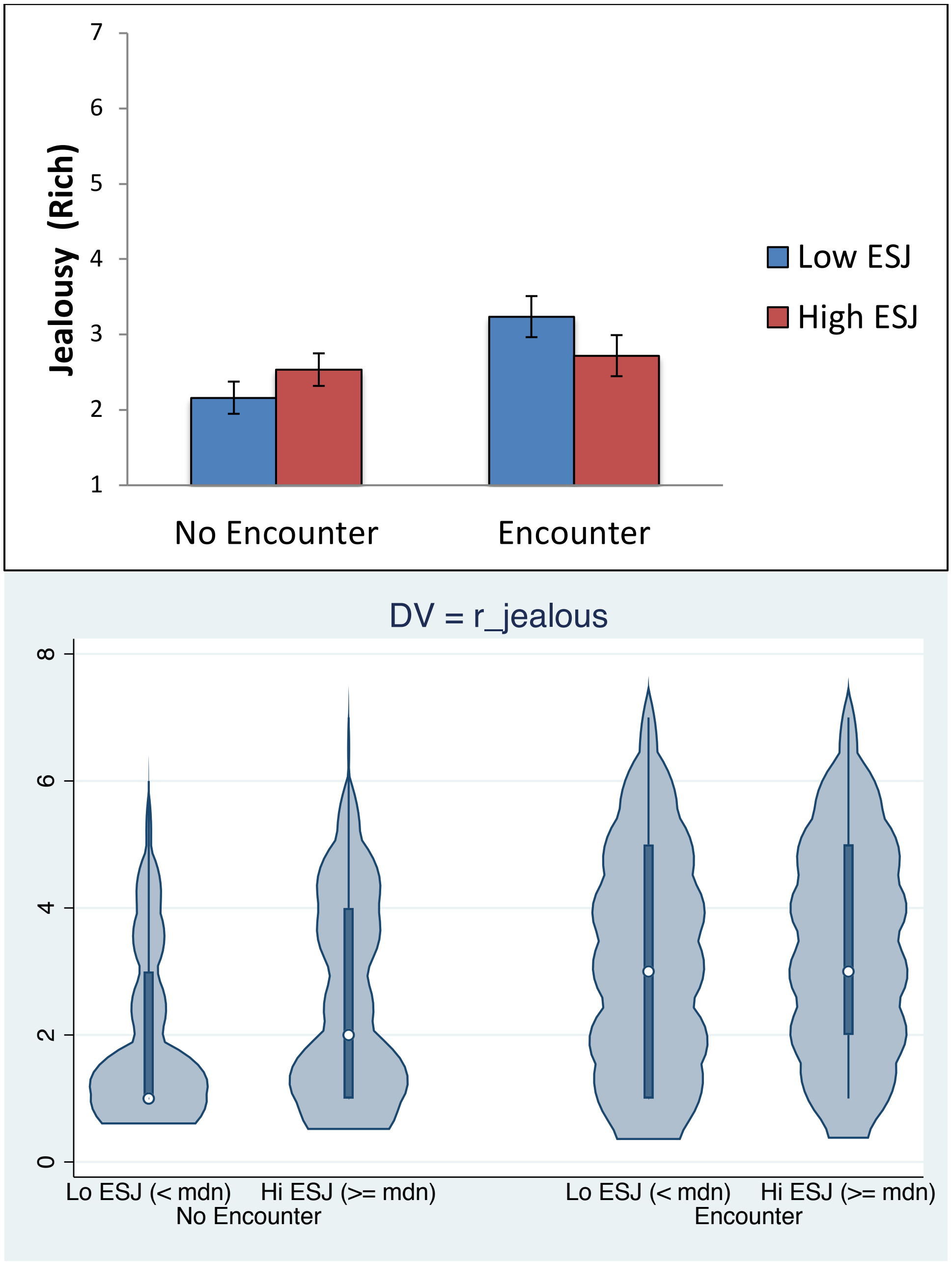

Supplementary Figure 56. *ESJ × Rich Encounter interaction for jealousy in Study 6, p = .014. Simple effect of ESJ in no encounter condition, p = .221; simple effect of ESJ in encounter condition, p = .179. Simple effect of encounter (vs. no encounter) at low (-1 SD) ESJ, p < .001; simple effect of encounter (vs. no encounter) at high (+1 SD) ESJ, p = 476. Emotion was rated on a 1 (not at all) to 7 (extremely) scale. Error bars represent standard errors. Bottom graph shows description statistics and distributional properties based on raw data.*

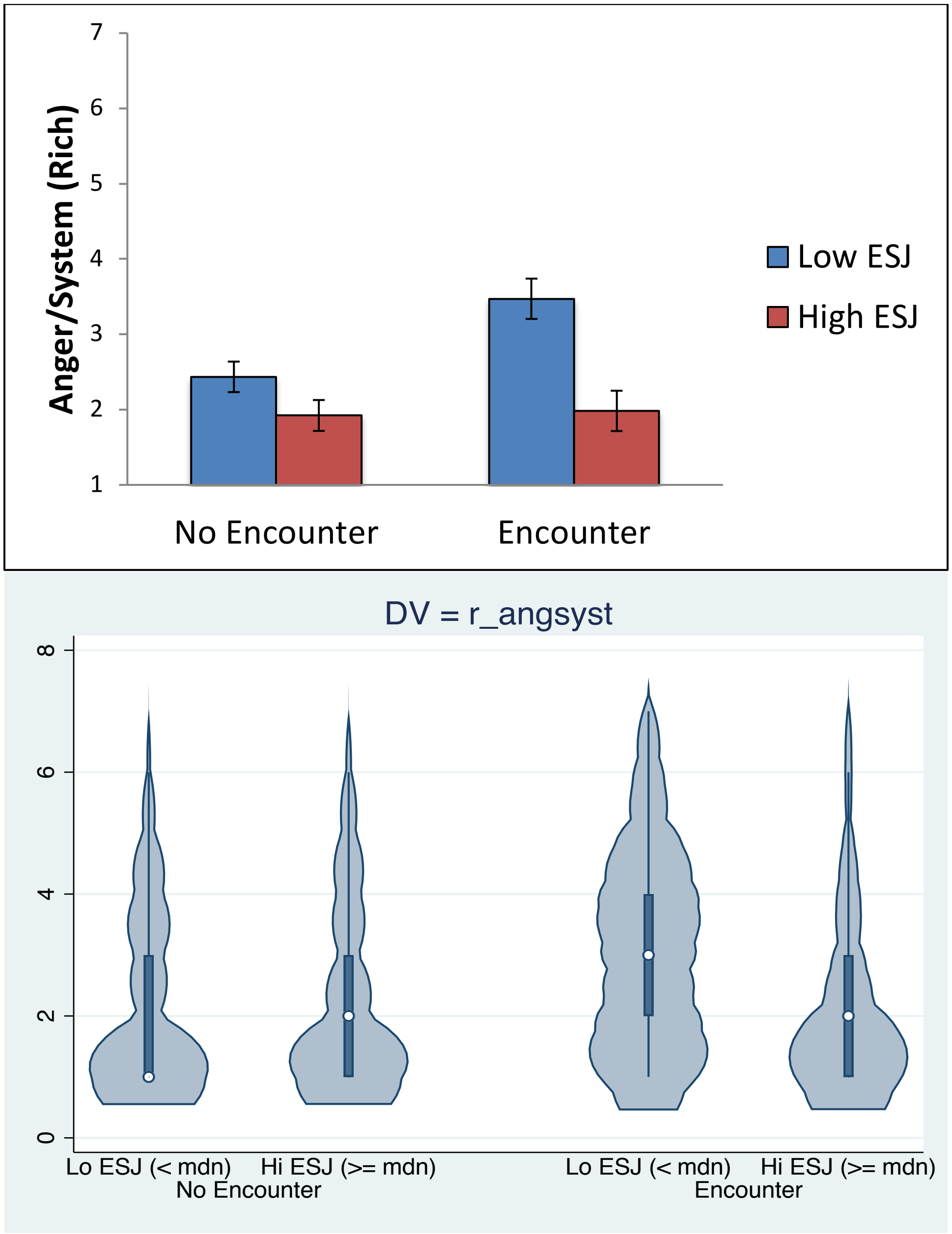

Supplementary Figure 57. *ESJ × Rich Encounter interaction for system-directed anger in Study 6, p = .008. Simple effect of ESJ in no encounter condition, p = .076; simple effect of ESJ in encounter condition, p < .001. Simple effect of encounter (vs. no encounter) at low (-1 SD) ESJ, p < .001; simple effect of encounter (vs. no encounter) at high (+1 SD) ESJ, p = .818. Emotion was rated on a 1 (not at all) to 7 (extremely) scale. Error bars represent standard errors. Bottom graph shows description statistics and distributional properties based on raw data.*

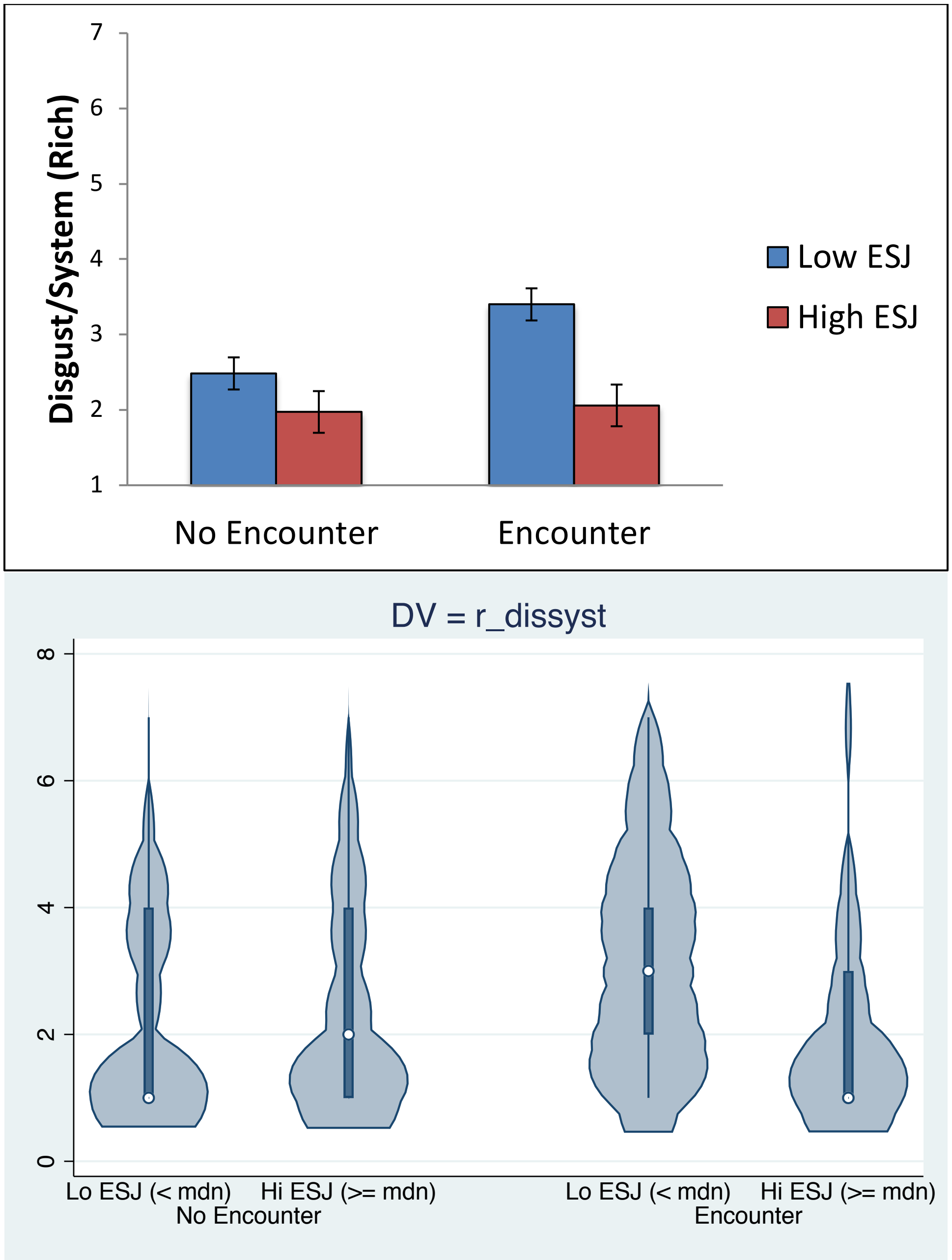

Supplementary Figure 58. *ESJ × Rich Encounter interaction for system-directed disgust in Study 6, p = .03. Simple effect of ESJ in no encounter condition, p = .089; simple effect of ESJ in encounter condition, p = .001. Simple effect of encounter (vs. no encounter) at low (-1 SD) ESJ, p = .001; simple effect of encounter (vs. no encounter) at high (+1 SD) ESJ, p = .752. Emotion was rated on a 1 (not at all) to 7 (extremely) scale. Error bars represent standard errors. Bottom graph shows description statistics and distributional properties based on raw data.*

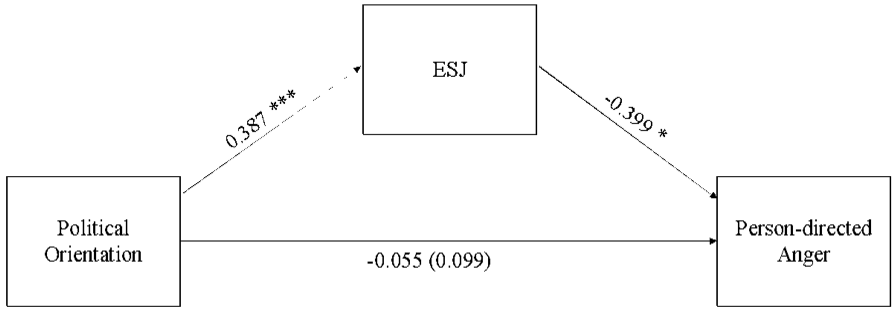

Supplementary Figure 59. *Regression coefficients for the relationship between political orientation and anger at the poor target as mediated by Economic System Justification (ESJ) in Study 6. The regression coefficient between political orientation and anger at the poor target, adjusting for ESJ, is in parentheses.* * p < .05, *** p < .001.

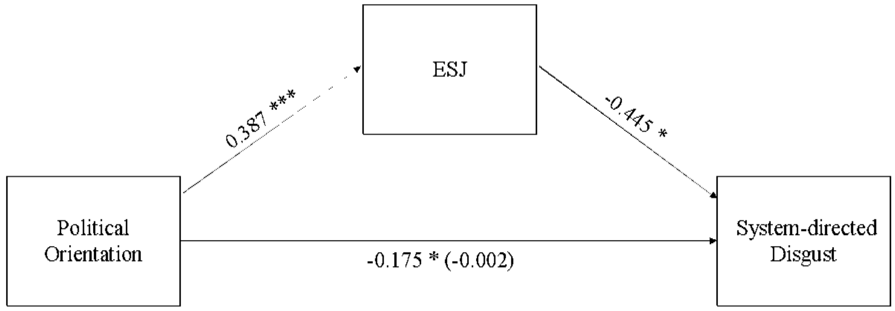

Supplementary Figure 60. *Regression coefficients for the relationship between political orientation and disgust at the system when encountering poor target as mediated by Economic System Justification (ESJ) in Study 6. The regression coefficient between political orientation and disgust at the system, adjusting for ESJ, is in parentheses.* * p < .05, *** p < .001.

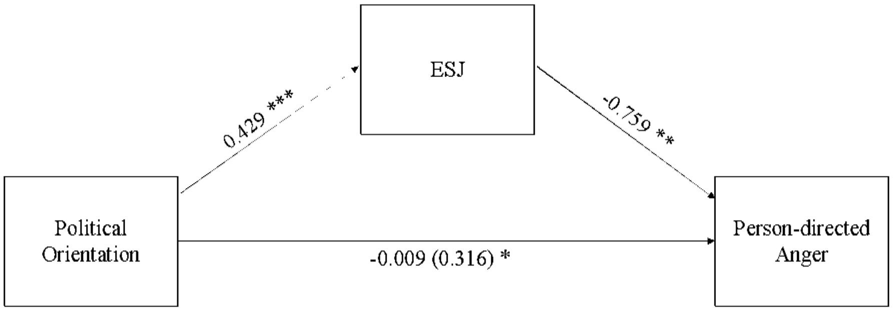

Supplementary Figure 61. *Regression coefficients for the relationship between political orientation and anger at the rich target as mediated by Economic System Justification (ESJ) in Study 6. The regression coefficient between political orientation and anger at the rich target, adjusting for ESJ, is in parentheses.* *$p < .05$, **$p < .001$, ***$p < .001$.

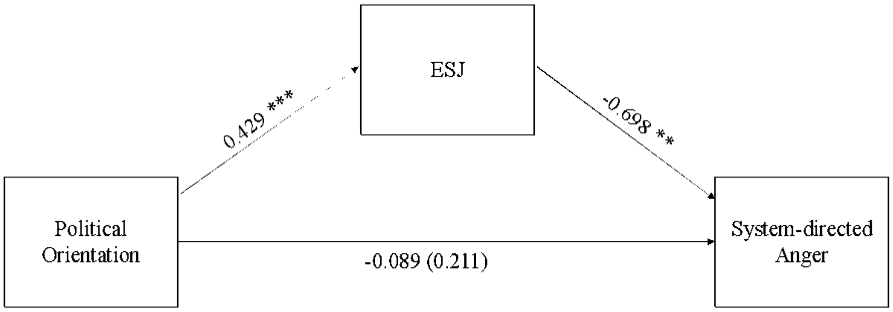

Supplementary Figure 62. *Regression coefficients for the relationship between political orientation and anger at the system when encountering rich target as mediated by Economic System Justification (ESJ) in Study 6. The regression coefficient between political orientation and anger at the system, adjusting for ESJ, is in parentheses.* **$p < .01$, ***$p < .001$.

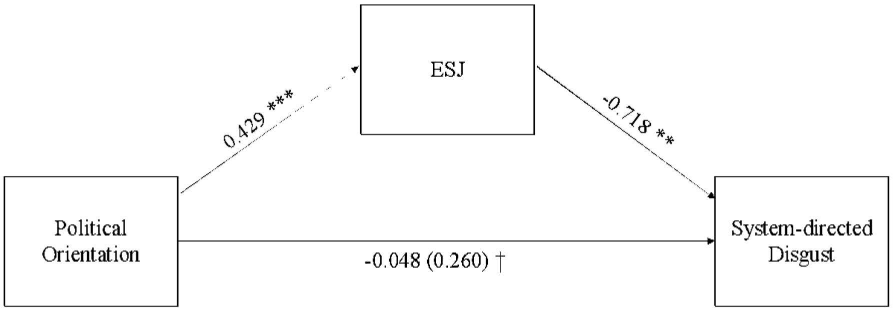

Supplementary Figure 63. *Regression coefficients for the relationship between political orientation and disgust at the system when encountering rich target as mediated by Economic System Justification (ESJ) in Study 6. The regression coefficient between political orientation and disgust at the system, adjusting for ESJ, is in parentheses.*† $p < .1$, ** $p < .01$, *** $p < .001$.

Supplementary Tables

Descriptive Statistics and Correlations (Study 1)

Supplementary Table 1. Person-directed emotions (homeless condition) in Study 1.

Emotion	M	SD	Anger	Sadness	Disgust	Guilt	Pity	Empathy
Anger	19.00	26.76	—	-0.10	0.70 ***	0.36 ***	0.05	-0.18 †
Sadness	70.50	25.70		—	-0.24 *	0.16	0.61 ***	0.75 ***
Disgust	14.54	22.22			—	0.27 **	-0.05	-0.37 ***
Guilt	35.78	31.42				—	0.21 *	0.11
Pity	64.55	27.02					—	0.55 ***
Empathy	75.49	23.21						—

Note. † $p < .10$, * $p < .05$, ** $p < .01$, *** $p < .001$. Emotions were rated on a 0–100 scale.

Supplementary Table 2. Person-directed emotions (control condition) in Study 1.

Emotion	M	SD	Anger	Sadness	Disgust	Guilt	Pity	Empathy
Anger	5.22	12.68	—	0.58 ***	0.88 ***	0.57 ***	0.59 ***	0.18 †
Sadness	6.88	13.64		—	0.63 ***	0.89 ***	0.85 ***	0.30 **
Disgust	7.15	16.27			—	0.70 ***	0.60 ***	0.19 †
Guilt	6.01	14.11				—	0.75 ***	0.23 **
Pity	7.10	13.29					—	0.34 ***
Empathy	21.82	26.57						—

Note. † $p < .10$, ** $p < .01$, *** $p < .001$. Emotions were rated on a 0–100 scale.

Supplementary Table 3. System-directed emotions (homeless condition) in Study 1.

Emotion	M	SD	Anger	Sadness	Disgust	Guilt	Pride	Hope
Anger	57.50	31.88	—	0.64 ***	0.77 ***	0.50 ***	-0.14	-0.05
Sadness	64.65	26.75		—	0.64 ***	0.53 ***	-0.17 †	-0.01
Disgust	58.30	31.94			—	0.39 ***	-0.15	-0.04
Guilt	42.47	31.78				—	0.09	0.18 †
Pride	19.66	22.15					—	0.67 ***
Hope	29.47	24.36						—

Note. † $p < .10$, *** $p < .001$. Emotions were rated on a 0–100 scale.

Supplementary Table 4. System-directed emotions (control condition) in Study 1.

Emotion	M	SD	Anger	Sadness	Disgust	Guilt	Pride	Hope
Anger	21.49	27.60	—	0.86 ***	0.92 ***	0.72 ***	-0.03	-0.02
Sadness	19.96	25.61		—	0.87 ***	0.69 ***	-0.04	0.00
Disgust	21.89	28.22			—	0.67 ***	-0.06	-0.06
Guilt	16.94	24.34				—	0.13	0.11
Pride	37.78	30.81					—	0.80 ***
Hope	39.50	29.68						—

Note. *** $p < .001$. Emotions were rated on a 0–100 scale.

Full Regression Results (Study 1)

Supplementary Table 5. Full regression results for analysis of person-directed anger in Study 1.

Predictor	B	SE B	*z*	*p*	CI LB	CI UB
Order (O)	2.241	1.260	1.779	0.075	-0.228	4.710
Economic System Justification (ESJ)	1.361	0.908	1.499	0.134	-0.418	3.140
O × ESJ	0.141	0.896	0.157	0.875	-1.615	1.897
Video Type (V)	13.636	2.329	5.856	0.000	9.072	18.200
V × O	-0.426	2.342	-0.182	0.856	-5.017	4.165
**V × ESJ**	**0.861**	**2.197**	**0.392**	**0.695**	**-3.445**	**5.167**
O × V × ESJ	-2.656	2.178	-1.219	0.223	-6.925	1.613
Control Video 1 vs. 2	2.544	1.135	2.241	0.025	0.319	4.769
Homeless Video 1 vs. 4	0.823	4.885	0.168	0.866	-8.751	10.397
Homeless Video 2 vs. 4	-8.875	3.174	-2.796	0.005	-15.096	-2.655
Homeless Video 3 vs. 4	0.761	4.269	0.178	0.858	-7.605	9.127
Intercept	5.117	1.118	4.578	0.000	2.927	7.308

Note. Video Type is coded such that 1 = homeless and 0 = control. Emotion was rated on a 0–100 scale.

Supplementary Table 6. Full regression results for analysis of person-directed sadness in Study 1.

Predictor	B	SE B	*z*	*p*	CI LB	CI UB
Order (O)	-0.538	1.419	-0.379	0.705	-3.318	2.243
Economic System Justification (ESJ)	2.242	1.034	2.168	0.030	0.215	4.268
O × ESJ	-1.500	0.998	-1.504	0.133	-3.456	0.455
Video Type (V)	63.708	2.841	22.427	0.000	58.141	69.276
V × O	-4.038	2.944	-1.372	0.170	-9.808	1.732
**V × ESJ**	**-9.274**	**2.594**	**-3.575**	**0.000**	**-14.357**	**-4.190**
O × V × ESJ	4.923	2.464	1.998	0.046	0.094	9.753
Control Video 1 vs. 2	2.041	1.387	1.471	0.141	-0.679	4.760
Homeless Video 1 vs. 4	-0.058	4.025	-0.014	0.989	-7.947	7.831
Homeless Video 2 vs. 4	-2.227	3.944	-0.565	0.572	-9.957	5.503
Homeless Video 3 vs. 4	-0.593	4.114	-0.144	0.885	-8.656	7.471
Intercept	6.812	1.283	5.307	0.000	4.296	9.327

Note. Video Type is coded such that 1 = homeless and 0 = control. Emotion was rated on a 0–100 scale.

Supplementary Table 7. Full regression results for analysis of person-directed disgust in Study 1.

Predictor	B	SE B	*z*	*p*	CI LB	CI UB
Order (O)	1.796	1.622	1.107	0.268	-1.384	4.976
Economic System Justification (ESJ)	1.754	1.185	1.481	0.139	-0.568	4.077
O × ESJ	-1.292	1.203	-1.075	0.282	-3.649	1.065
Video Type (V)	7.444	2.141	3.477	0.001	3.248	11.641
V × O	-1.880	2.236	-0.841	0.400	-6.264	2.503
**V × ESJ**	**0.906**	**1.831**	**0.495**	**0.621**	**-2.682**	**4.495**
O × V × ESJ	1.377	1.794	0.767	0.443	-2.140	4.894
Control Video 1 vs. 2	3.796	1.488	2.550	0.011	0.879	6.713
Homeless Video 1 vs. 4	0.375	4.031	0.093	0.926	-7.526	8.275
Homeless Video 2 vs. 4	-7.638	2.254	-3.388	0.001	-12.056	-3.219
Homeless Video 3 vs. 4	3.813	4.156	0.917	0.359	-4.333	11.959
Intercept	6.935	1.458	4.758	0.000	4.078	9.792

Note. Video Type is coded such that 1 = homeless and 0 = control. Emotion was rated on a 0–100 scale.

Supplementary Table 8. Full regression results for analysis of person-directed guilt in Study 1.

Predictor	B	SE B	*z*	*p*	CI LB	CI UB
Order (O)	0.137	1.529	0.090	0.929	-2.859	3.133
Economic System Justification (ESJ)	1.958	1.069	1.831	0.067	-0.138	4.054
O × ESJ	-1.228	0.970	-1.266	0.206	-3.129	0.673
Video Type (V)	30.026	2.911	10.316	0.000	24.322	35.731
V × O	3.386	2.950	1.148	0.251	-2.395	9.168
**V × ESJ**	**-2.784**	**3.068**	**-0.907**	**0.364**	**-8.798**	**3.229**
O × V × ESJ	3.563	3.090	1.153	0.249	-2.493	9.620
Control Video 1 vs. 2	0.573	1.472	0.389	0.697	-2.312	3.458
Homeless Video 1 vs. 4	7.422	5.258	1.411	0.158	-2.884	17.728
Homeless Video 2 vs. 4	3.453	5.283	0.654	0.513	-6.902	13.808
Homeless Video 3 vs. 4	5.103	5.560	0.918	0.359	-5.795	16.000
Intercept	5.993	1.366	4.388	0.000	3.316	8.669

Note. Video Type is coded such that 1 = homeless and 0 = control. Emotion was rated on a 0–100 scale.

Supplementary Table 9. Full regression results for analysis of person-directed pity in Study 1.

Predictor	B	SE B	*z*	*p*	CI LB	CI UB
Order (O)	0.414	1.419	0.292	0.770	-2.366	3.195
Economic System Justification (ESJ)	2.128	0.975	2.182	0.029	0.217	4.039
O × ESJ	-1.637	0.957	-1.710	0.087	-3.514	0.239
Video Type (V)	57.336	2.774	20.667	0.000	51.899	62.773
V × O	-0.341	2.906	-0.117	0.907	-6.036	5.354
**V × ESJ**	**-10.729**	**2.809**	**-3.819**	**0.000**	**-16.235**	**-5.223**
O × V × ESJ	4.729	2.803	1.687	0.092	-0.764	10.222
Control Video 1 vs. 2	1.647	1.376	1.197	0.231	-1.050	4.343
Homeless Video 1 vs. 4	0.762	4.123	0.185	0.853	-7.319	8.844
Homeless Video 2 vs. 4	-5.294	4.349	-1.217	0.223	-13.818	3.229
Homeless Video 3 vs. 4	-0.521	4.460	-0.117	0.907	-9.263	8.221
Intercept	7.041	1.245	5.658	0.000	4.602	9.480

Note. Video Type is coded such that 1 = homeless and 0 = control. Emotion was rated on a 0–100 scale.

Supplementary Table 10. Full regression results for analysis of person-directed empathy in Study 1.

Predictor	B	SE B	*z*	*p*	CI LB	CI UB
Order (O)	-1.587	2.566	-0.618	0.536	-6.617	3.443
Economic System Justification (ESJ)	0.251	2.449	0.103	0.918	-4.548	5.051
O × ESJ	-3.739	2.440	-1.532	0.125	-8.521	1.043
Video Type (V)	53.708	3.129	17.166	0.000	47.576	59.840
V × O	-1.125	3.157	-0.356	0.721	-7.312	5.062
**V × ESJ**	**-7.363**	**3.061**	**-2.405**	**0.016**	**-13.362**	**-1.363**
O × V × ESJ	5.058	3.109	1.627	0.104	-1.035	11.151
Control Video 1 vs. 2	0.491	2.576	0.191	0.849	-4.559	5.540
Homeless Video 1 vs. 4	-0.198	3.752	-0.053	0.958	-7.552	7.155
Homeless Video 2 vs. 4	4.224	2.742	1.540	0.123	-1.150	9.598
Homeless Video 3 vs. 4	-4.492	4.444	-1.011	0.312	-13.202	4.218
Intercept	21.884	2.558	8.556	0.000	16.871	26.898

Note. Video Type is coded such that 1 = homeless and 0 = control. Emotion was rated on a 0–100 scale.

Supplementary Table 11. Full regression results for analysis of system-directed anger in Study 1.

Predictor	B	SE B	*z*	*p*	CI LB	CI UB
Order (O)	-0.466	2.699	-0.173	0.863	-5.756	4.824
Economic System Justification (ESJ)	-3.287	2.922	-1.125	0.261	-9.014	2.440
O × ESJ	2.218	2.945	0.753	0.451	-3.555	7.991
Video Type (V)	36.393	2.924	12.447	0.000	30.662	42.124
V × O	-3.467	3.007	-1.153	0.249	-9.362	2.427
**V × ESJ**	**-10.448**	**3.000**	**-3.482**	**0.000**	**-16.328**	**-4.567**
O × V × ESJ	2.909	3.032	0.959	0.337	-3.034	8.852
Control Video 1 vs. 2	5.688	2.475	2.298	0.022	0.837	10.539
Homeless Video 1 vs. 4	5.042	4.215	1.196	0.232	-3.219	13.304
Homeless Video 2 vs. 4	-1.474	4.763	-0.310	0.757	-10.810	7.861
Homeless Video 3 vs. 4	-2.722	4.342	-0.627	0.531	-11.232	5.787
Intercept	21.203	2.590	8.187	0.000	16.127	26.279

Note. Video Type is coded such that 1 = homeless and 0 = control. Emotion was rated on a 0–100 scale.

Supplementary Table 12. Full regression results for analysis of system-directed sadness in Study 1.

Predictor	B	SE B	*z*	*p*	CI LB	CI UB
Order (O)	-0.530	2.581	-0.206	0.837	-5.589	4.528
Economic System Justification (ESJ)	-1.091	2.661	-0.410	0.682	-6.307	4.124
O × ESJ	2.038	2.736	0.745	0.457	-3.326	7.401
Video Type (V)	45.074	2.800	16.096	0.000	39.585	50.563
V × O	-2.989	2.912	-1.026	0.305	-8.695	2.718
**V × ESJ**	**-9.532**	**2.922**	**-3.262**	**0.001**	**-15.259**	**-3.805**
O × V × ESJ	-1.571	2.946	-0.533	0.594	-7.344	4.203
Control Video 1 vs. 2	5.065	2.426	2.088	0.037	0.311	9.820
Homeless Video 1 vs. 4	0.435	4.136	0.105	0.916	-7.671	8.541
Homeless Video 2 vs. 4	-2.423	3.794	-0.639	0.523	-9.859	5.014
Homeless Video 3 vs. 4	4.636	3.983	1.164	0.245	-3.172	12.443
Intercept	19.712	2.424	8.133	0.000	14.962	24.463

Note. Homeless is coded such that 1 = homeless and 0 = control. Emotion was rated on a 0–100 scale.

Supplementary Table 13. Full regression results for analysis of system-directed disgust in Study 1.

Predictor	B	SE B	*z*	*p*	CI LB	CI UB
Order (O)	-0.348	2.807	-0.124	0.901	-5.850	5.154
Economic System Justification (ESJ)	-3.820	3.020	-1.265	0.206	-9.739	2.098
O × ESJ	2.065	3.032	0.681	0.496	-3.877	8.007
Video Type (V)	36.762	3.086	11.913	0.000	30.714	42.811
V × O	-2.523	3.259	-0.774	0.439	-8.909	3.864
**V × ESJ**	**-13.338**	**3.149**	**-4.235**	**0.000**	**-19.510**	**-7.165**
O × V × ESJ	0.550	3.046	0.180	0.857	-5.420	6.520
Control Video 1 vs. 2	5.044	2.705	1.865	0.062	-0.257	10.345
Homeless Video 1 vs. 4	1.819	4.849	0.375	0.708	-7.685	11.323
Homeless Video 2 vs. 4	-0.382	4.707	-0.081	0.935	-9.608	8.845
Homeless Video 3 vs. 4	1.221	4.102	0.298	0.766	-6.819	9.261
Intercept	21.632	2.642	8.186	0.000	16.452	26.811

Note. Homeless is coded such that 1 = homeless and 0 = control. Emotion was rated on a 0–100 scale.

Supplementary Table 14. Full regression results for analysis of system-directed guilt in Study 1.

Predictor	B	SE B	*z*	*p*	CI LB	CI UB
Order (O)	4.634	2.343	1.978	0.048	0.043	9.226
Economic System Justification (ESJ)	1.988	2.082	0.955	0.340	-2.093	6.068
O × ESJ	-0.444	2.024	-0.219	0.827	-4.411	3.523
Video Type (V)	26.313	2.913	9.034	0.000	20.604	32.022
V × O	-5.672	3.012	-1.883	0.060	-11.576	0.232
**V × ESJ**	**-5.405**	**2.931**	**-1.844**	**0.065**	**-11.150**	**0.340**
O × V × ESJ	1.903	2.956	0.644	0.520	-3.891	7.697
Control Video 1 vs. 2	5.410	2.104	2.572	0.010	1.287	9.533
Homeless Video 1 vs. 4	11.786	4.965	2.374	0.018	2.055	21.517
Homeless Video 2 vs. 4	2.845	5.121	0.555	0.579	-7.192	12.882
Homeless Video 3 vs. 4	-7.029	4.945	-1.421	0.155	-16.720	2.663
Intercept	16.338	2.198	7.435	0.000	12.031	20.645

Note. Homeless is coded such that 1 = homeless and 0 = control. Emotion was rated on a 0–100 scale.

Supplementary Table 15. Full regression results for analysis of system-directed pride in Study 1.

Predictor	B	SE B	*z*	*p*	CI LB	CI UB
Order (O)	-0.648	2.785	-0.233	0.816	-6.107	4.810
Economic System Justification (ESJ)	11.480	3.013	3.811	0.000	5.575	17.385
O × ESJ	-0.345	2.965	-0.116	0.907	-6.156	5.466
Video Type (V)	-18.431	2.652	-6.949	0.000	-23.630	-13.233
V × O	0.855	2.553	0.335	0.738	-4.150	5.859
**V × ESJ**	**-1.881**	**2.601**	**-0.723**	**0.470**	**-6.978**	**3.217**
O × V × ESJ	-1.751	2.509	-0.698	0.485	-6.670	3.167
Control Video 1 vs. 2	-2.552	2.592	-0.984	0.325	-7.632	2.529
Homeless Video 1 vs. 4	-3.850	2.910	-1.323	0.186	-9.553	1.853
Homeless Video 2 vs. 4	1.120	2.783	0.402	0.687	-4.335	6.575
Homeless Video 3 vs. 4	1.681	3.650	0.461	0.645	-5.473	8.835
Intercept	37.925	2.839	13.360	0.000	32.361	43.489

Note. Homeless is coded such that 1 = homeless and 0 = control. Emotion was rated on a 0–100 scale.

Supplementary Table 16. Full regression results for analysis of system-directed hope in Study 1.

Predictor	B	SE B	*z*	*p*	CI LB	CI UB
Order (O)	-0.388	2.752	-0.141	0.888	-5.782	5.006
Economic System Justification (ESJ)	7.263	2.932	2.477	0.013	1.516	13.010
O × ESJ	2.435	2.896	0.841	0.401	-3.242	8.111
Video Type (V)	-10.408	2.685	-3.877	0.000	-15.670	-5.147
V × O	0.992	2.689	0.369	0.712	-4.277	6.262
**V × ESJ**	**-0.049**	**2.763**	**-0.018**	**0.986**	**-5.464**	**5.366**
O × V × ESJ	-3.250	2.602	-1.249	0.212	-8.350	1.851
Control Video 1 vs. 2	-3.997	2.500	-1.599	0.110	-8.896	0.903
Homeless Video 1 vs. 4	-4.349	2.818	-1.543	0.123	-9.872	1.174
Homeless Video 2 vs. 4	-0.668	3.552	-0.188	0.851	-7.629	6.292
Homeless Video 3 vs. 4	6.278	3.700	1.697	0.090	-0.973	13.530
Intercept	39.668	2.802	14.158	0.000	34.177	45.160

Note. Homeless is coded such that 1 = homeless and 0 = control. Emotion was rated on a 0–100 scale.

Supplementary Table 17. Robustness check for analysis person-directed sadness in Study 1.

Predictor	B	SE B	*z*	*p*	CI LB	CI UB
Order (O)	0.088	1.411	0.062	0.950	-2.677	2.853
Economic System Justification (ESJ)	0.427	1.269	0.337	0.736	-2.060	2.915
O × ESJ	-0.745	1.045	-0.713	0.476	-2.794	1.304
Video Type (V)	63.657	2.797	22.762	0.000	58.176	69.139
V × O	-4.383	2.871	-1.527	0.127	-10.010	1.244
**V × ESJ**	**-9.885**	**2.766**	**-3.574**	**0.000**	**-15.306**	**-4.465**
O × V × ESJ	3.861	2.673	1.444	0.149	-1.378	9.100
BIDR Self-Deceptive Enhancement (SDE)	-0.372	1.595	-0.233	0.815	-3.498	2.754
O × SDE	-0.042	1.560	-0.027	0.978	-3.101	3.016
V × SDE	4.961	3.408	1.456	0.146	-1.719	11.642
V × O × SDE	1.225	3.464	0.354	0.724	-5.564	8.014
BIDR Impression Management (IM)	-2.099	1.527	-1.375	0.169	-5.092	0.893
O × IM	1.103	1.337	0.825	0.410	-1.518	3.724
V × IM	-2.346	3.185	-0.737	0.461	-8.589	3.897
V × O × IM	-0.252	3.250	-0.078	0.938	-6.622	6.118
Control Video 1 vs. 2	1.574	1.403	1.122	0.262	-1.176	4.324
Homeless Video 1 vs. 4	-1.062	3.823	-0.278	0.781	-8.556	6.431
Homeless Video 2 vs. 4	0.440	4.049	0.109	0.913	-7.495	8.376
Homeless Video 3 vs. 4	-1.325	3.949	-0.336	0.737	-9.064	6.414
Religiosity	1.517	0.513	2.956	0.003	0.511	2.523
Black	7.674	4.405	1.742	0.081	-0.959	16.307
Latinx	14.192	7.161	1.982	0.048	0.156	28.229
Asian	2.414	3.809	0.634	0.526	-5.052	9.879
Other race	-6.490	7.515	-0.864	0.388	-21.220	8.239
Age	-0.155	0.095	-1.629	0.103	-0.342	0.032
Gender	8.834	2.726	3.240	0.001	3.490	14.177
Income	1.794	0.709	2.529	0.011	0.404	3.184
Intercept	-14.225	6.256	-2.274	0.023	-26.486	-1.964

Note. BIDR = Balanced Inventory of Desirable Responding. Homeless is coded such that 1 = homeless and 0 = control. Emotion was rated on a 0–100 scale.

Supplementary Table 18. Robustness check for analysis person-directed pity in Study 1.

Predictor	B	SE B	*z*	*p*	CI LB	CI UB
Order (O)	0.286	1.491	0.192	0.848	-2.638	3.209
Economic System Justification (ESJ)	0.784	1.245	0.629	0.529	-1.657	3.224
O × ESJ	-0.863	1.039	-0.830	0.406	-2.898	1.173
Video Type (V)	57.556	2.733	21.059	0.000	52.199	62.913
V × O	-1.000	2.876	-0.348	0.728	-6.636	4.636
**V × ESJ**	**-11.130**	**3.015**	**-3.692**	**0.000**	**-17.038**	**-5.221**
O × V × ESJ	3.381	2.995	1.129	0.259	-2.490	9.251
BIDR Self-Deceptive Enhancement (SDE)	-0.877	1.440	-0.609	0.542	-3.699	1.944
O × SDE	-0.748	1.382	-0.541	0.589	-3.457	1.962
V × SDE	2.822	3.749	0.753	0.452	-4.527	10.170
V × O × SDE	3.733	3.831	0.975	0.330	-3.775	11.242
BIDR Impression Management (IM)	-1.257	1.454	-0.864	0.387	-4.107	1.594
O × IM	1.494	1.199	1.246	0.213	-0.855	3.844
V × IM	-1.288	3.663	-0.352	0.725	-8.466	5.891
V × O × IM	-2.309	3.702	-0.624	0.533	-9.564	4.946
Control Video 1 vs. 2	1.061	1.414	0.750	0.453	-1.712	3.833
Homeless Video 1 vs. 4	1.507	3.950	0.382	0.703	-6.235	9.248
Homeless Video 2 vs. 4	-3.041	4.759	-0.639	0.523	-12.368	6.287
Homeless Video 3 vs. 4	-2.438	4.669	-0.522	0.602	-11.589	6.714
Religiosity	1.287	0.672	1.916	0.055	-0.030	2.604
Black	3.266	4.560	0.716	0.474	-5.672	12.203
Latinx	-34.228	10.754	-3.183	0.001	-55.306	-13.151
Asian	-2.205	3.873	-0.569	0.569	-9.796	5.387
Other race	-12.366	7.119	-1.737	0.082	-26.319	1.587
Age	-0.058	0.111	-0.520	0.603	-0.275	0.160
Gender	2.395	3.291	0.728	0.467	-4.057	8.846
Income	1.134	0.758	1.496	0.135	-0.352	2.621
Intercept	-0.465	7.295	-0.064	0.949	-14.762	13.833

Note. BIDR = Balanced Inventory of Desirable Responding. Homeless is coded such that 1 = homeless and 0 = control. Emotion was rated on a 0–100 scale.

Supplementary Table 19. Robustness check for analysis person-directed empathy in Study 1.

Predictor	B	SE B	*z*	*p*	CI LB	CI UB
Order (O)	-0.238	2.636	-0.090	0.928	-5.403	4.928
Economic System Justification (ESJ)	0.285	2.474	0.115	0.908	-4.563	5.133
O × ESJ	-2.528	2.447	-1.033	0.302	-7.324	2.269
Video Type (V)	53.578	3.074	17.430	0.000	47.554	59.603
V × O	-1.646	3.098	-0.531	0.595	-7.718	4.427
**V × ESJ**	**-8.079**	**3.245**	**-2.490**	**0.013**	**-14.438**	**-1.719**
O × V × ESJ	3.410	3.259	1.046	0.295	-2.978	9.797
BIDR Self-Deceptive Enhancement (SDE)	-1.453	3.335	-0.436	0.663	-7.989	5.083
O × SDE	-3.903	3.269	-1.194	0.232	-10.310	2.503
V × SDE	6.569	3.824	1.718	0.086	-0.926	14.065
V × O × SDE	2.672	3.858	0.693	0.489	-4.889	10.233
BIDR Impression Management (IM)	-1.655	3.476	-0.476	0.634	-8.467	5.157
O × IM	4.811	3.412	1.410	0.158	-1.875	11.498
V × IM	-1.985	3.482	-0.570	0.569	-8.810	4.840
V × O × IM	-1.945	3.633	-0.535	0.592	-9.066	5.176
Control Video 1 vs. 2	0.458	2.524	0.182	0.856	-4.488	5.404
Homeless Video 1 vs. 4	-1.491	3.861	-0.386	0.699	-9.059	6.077
Homeless Video 2 vs. 4	5.829	3.470	1.680	0.093	-0.973	12.631
Homeless Video 3 vs. 4	-5.132	4.153	-1.236	0.217	-13.271	3.008
Religiosity	0.217	0.840	0.258	0.796	-1.430	1.863
Black	-1.006	7.087	-0.142	0.887	-14.896	12.884
Latinx	4.544	10.564	0.430	0.667	-16.160	25.249
Asian	5.091	6.329	0.804	0.421	-7.314	17.496
Other race	-8.714	10.226	-0.852	0.394	-28.757	11.329
Age	0.018	0.157	0.116	0.908	-0.290	0.326
Gender	11.205	4.514	2.482	0.013	2.359	20.051
Income	1.360	1.094	1.244	0.213	-0.783	3.504
Intercept	-4.831	9.405	-0.514	0.608	-23.263	13.602

Note. BIDR = Balanced Inventory of Desirable Responding. Homeless is coded such that 1 = homeless and 0 = control. Emotion was rated on a 0–100 scale.

Supplementary Table 20. Robustness check for analysis system-directed anger in Study 1.

Predictor	B	SE B	*z*	*p*	CI LB	CI UB
Order (O)	-0.983	2.716	-0.362	0.717	-6.306	4.340
Economic System Justification (ESJ)	-5.103	2.825	-1.807	0.071	-10.639	0.433
O × ESJ	3.735	2.846	1.312	0.189	-1.843	9.312
Video Type (V)	36.512	2.995	12.189	0.000	30.641	42.383
V × O	-3.439	3.082	-1.116	0.264	-9.479	2.602
**V × ESJ**	**-10.512**	**3.023**	**-3.478**	**0.001**	**-16.436**	**-4.587**
O × V × ESJ	2.718	3.035	0.896	0.370	-3.230	8.667
BIDR Self-Deceptive Enhancement (SDE)	-0.969	3.916	-0.247	0.805	-8.645	6.707
O × SDE	-0.104	3.667	-0.028	0.977	-7.291	7.084
V × SDE	0.193	4.219	0.046	0.963	-8.076	8.462
V × O × SDE	-0.390	4.256	-0.092	0.927	-8.731	7.951
BIDR Impression Management (IM)	-2.704	3.702	-0.730	0.465	-9.961	4.552
O × IM	-0.705	3.500	-0.201	0.840	-7.565	6.154
V × IM	0.517	3.355	0.154	0.878	-6.059	7.092
V × O × IM	-0.688	3.487	-0.197	0.844	-7.523	6.147
Control Video 1 vs. 2	4.504	2.569	1.753	0.080	-0.531	9.538
Homeless Video 1 vs. 4	7.373	4.587	1.607	0.108	-1.617	16.363
Homeless Video 2 vs. 4	-2.234	4.592	-0.487	0.627	-11.234	6.766
Homeless Video 3 vs. 4	-2.877	4.485	-0.641	0.521	-11.668	5.914
Religiosity	3.286	0.909	3.615	0.000	1.504	5.067
Black	8.407	12.433	0.676	0.499	-15.961	32.775
Latinx	1.699	15.681	0.108	0.914	-29.036	32.434
Asian	-9.704	11.805	-0.822	0.411	-32.842	13.434
Other race	-17.419	15.475	-1.126	0.260	-47.749	12.911
Age	-0.085	0.173	-0.490	0.624	-0.424	0.254
Gender	-1.756	4.864	-0.361	0.718	-11.288	7.777
Income	-1.093	1.328	-0.824	0.410	-3.695	1.509
Intercept	29.294	15.801	1.854	0.064	-1.676	60.264

Note. BIDR = Balanced Inventory of Desirable Responding. Homeless is coded such that 1 = homeless and 0 = control. Emotion was rated on a 0–100 scale.

Supplementary Table 21. Robustness check for analysis system-directed sadness in Study 1.

Predictor	B	SE B	*z*	*p*	CI LB	CI UB
Order (O)	-0.306	2.556	-0.120	0.905	-5.316	4.704
Economic System Justification (ESJ)	-2.467	2.425	-1.018	0.309	-7.219	2.285
O × ESJ	3.277	2.619	1.251	0.211	-1.857	8.412
Video Type (V)	44.953	2.852	15.761	0.000	39.362	50.543
V × O	-2.804	2.968	-0.945	0.345	-8.620	3.013
**V × ESJ**	**-9.698**	**2.993**	**-3.240**	**0.001**	**-15.565**	**-3.831**
O × V × ESJ	-1.119	2.971	-0.377	0.706	-6.942	4.703
BIDR Self-Deceptive Enhancement (SDE)	-2.491	3.475	-0.717	0.473	-9.303	4.320
O × SDE	1.127	3.340	0.337	0.736	-5.419	7.672
V × SDE	-1.633	4.126	-0.396	0.692	-9.719	6.454
V × O × SDE	0.756	4.213	0.179	0.858	-7.501	9.013
BIDR Impression Management (IM)	-2.683	3.901	-0.688	0.492	-10.328	4.963
O × IM	-0.750	3.654	-0.205	0.837	-7.911	6.411
V × IM	1.384	3.613	0.383	0.702	-5.698	8.466
V × O × IM	2.104	3.669	0.574	0.566	-5.087	9.295
Control Video 1 vs. 2	4.398	2.647	1.662	0.097	-0.789	9.586
Homeless Video 1 vs. 4	0.715	3.977	0.180	0.857	-7.080	8.510
Homeless Video 2 vs. 4	-1.686	4.141	-0.407	0.684	-9.804	6.431
Homeless Video 3 vs. 4	4.125	4.169	0.989	0.322	-4.046	12.296
Religiosity	2.049	0.874	2.344	0.019	0.336	3.761
Black	-2.925	11.440	-0.256	0.798	-25.346	19.496
Latinx	2.016	13.986	0.144	0.885	-25.397	29.428
Asian	-7.441	10.485	-0.710	0.478	-27.991	13.110
Other race	-13.976	13.486	-1.036	0.300	-40.409	12.457
Age	-0.016	0.149	-0.108	0.914	-0.309	0.277
Gender	1.445	4.412	0.327	0.743	-7.202	10.091
Income	-0.279	1.204	-0.232	0.817	-2.638	2.080
Intercept	19.765	13.375	1.478	0.139	-6.449	45.979

Note. BIDR = Balanced Inventory of Desirable Responding. Homeless is coded such that 1 = homeless and 0 = control. Emotion was rated on a 0–100 scale.

Supplementary Table 22. Robustness check for analysis system-directed disgust in Study 1.

Predictor	B	SE B	*z*	*p*	CI LB	CI UB
Order (O)	-1.550	2.805	-0.552	0.581	-7.049	3.949
Economic System Justification (ESJ)	-5.582	2.841	-1.965	0.049	-11.151	-0.013
O × ESJ	3.400	2.921	1.164	0.244	-2.325	9.124
Video Type (V)	36.486	3.108	11.738	0.000	30.394	42.578
V × O	-2.320	3.297	-0.704	0.482	-8.782	4.142
**V × ESJ**	**-14.953**	**2.772**	**-5.394**	**0.000**	**-20.387**	**-9.520**
O × V × ESJ	0.646	2.684	0.241	0.810	-4.615	5.908
BIDR Self-Deceptive Enhancement (SDE)	-1.195	4.148	-0.288	0.773	-9.326	6.935
O × SDE	-0.547	3.952	-0.138	0.890	-8.292	7.199
V × SDE	1.258	4.575	0.275	0.783	-7.707	10.224
V × O × SDE	2.788	4.557	0.612	0.541	-6.143	11.719
BIDR Impression Management (IM)	-2.951	4.323	-0.683	0.495	-11.423	5.521
O × IM	-1.733	4.262	-0.407	0.684	-10.087	6.621
V × IM	-5.065	4.869	-1.040	0.298	-14.607	4.478
V × O × IM	0.343	4.898	0.070	0.944	-9.258	9.944
Control Video 1 vs. 2	3.404	2.846	1.196	0.232	-2.175	8.982
Homeless Video 1 vs. 4	3.357	4.559	0.736	0.462	-5.578	12.293
Homeless Video 2 vs. 4	0.540	4.771	0.113	0.910	-8.811	9.890
Homeless Video 3 vs. 4	0.283	4.325	0.065	0.948	-8.194	8.759
Religiosity	2.078	0.886	2.345	0.019	0.341	3.815
Black	5.638	13.294	0.424	0.671	-20.417	31.693
Latinx	-9.060	16.049	-0.565	0.572	-40.516	22.396
Asian	-2.649	11.687	-0.227	0.821	-25.555	20.258
Other race	-17.697	15.100	-1.172	0.241	-47.293	11.898
Age	-0.237	0.176	-1.350	0.177	-0.582	0.107
Gender	-2.144	4.506	-0.476	0.634	-10.976	6.689
Income	-0.521	1.220	-0.427	0.670	-2.913	1.871
Intercept	32.143	14.959	2.149	0.032	2.823	61.462

Note. BIDR = Balanced Inventory of Desirable Responding. Homeless is coded such that 1 = homeless and 0 = control. Emotion was rated on a 0–100 scale.

Descriptive Statistics and Correlations (Study 2)

Supplementary Table 23. Person-directed emotions (control condition) in Study 2.

Emotion	M	SD	Sadness	Pity	Empathy
Sadness	8.95	18.24	—	0.86 ***	0.49 ***
Pity	9.09	18.15		—	0.47 ***
Empathy	25.91	27.35			—

Note. *** *p* < .001. Emotions were rated on a 0–100 scale.

Supplementary Table 24. System-directed emotions (control condition) in Study 2.

Emotion	M	SD	Anger	Sadness	Disgust
Anger	16.94	25.89	—	0.92 ***	0.93 **
Sadness	18.22	25.99		—	0.92 ***
Disgust	17.96	26.52			—

Note. ** *p* < .01, *** *p* < .001. Emotions were rated on a 0–100 scale.

Supplementary Table 25. Person-directed emotions (homeless condition) in Study 2.

Emotion	M	SD	Sadness	Pity	Empathy
Sadness	76.05	24.93	—	0.63 ***	0.73 ***
Pity	70.16	26.94		—	0.48 ***
Empathy	79.98	22.26			—

Note. ****p* < .001. Emotions were rated on a 0–100 scale.

Supplementary Table 26. System-directed emotions (homeless condition) in Study 2.

Emotion	M	SD	Anger	Sadness	Disgust
Anger	62.16	32.04	—	0.67 ***	0.86 ***
Sadness	66.92	28.82		—	0.65 ***
Disgust	63.45	32.67			—

Note. *** *p* < .001. Emotions were rated on a 0–100 scale.

Supplementary Table 27. Person-directed emotions (CF condition) in Study 2.

Emotion	M	SD	Sadness	Pity	Empathy
Sadness	78.84	22.60	—	0.48 ***	0.72 ***
Pity	67.95	27.56		—	0.45 ***
Empathy	82.63	19.78			—

Note. *** *p* < .001. Emotions were rated on a 0–100 scale.

Supplementary Table 28. System-directed emotions (CF condition) in Study 2.

Emotion	M	SD	Anger	Sadness	Disgust
Anger	40.27	33.78	—	0.73 ***	0.92 ***
Sadness	50.62	34.55		—	0.69 ***
Disgust	40.32	33.90			—

Note. *** p < .001. Emotions were rated on a 0–100 scale.

Full Regression Results (Study 2)

Supplementary Table 29. Full regression results for analysis of person-directed sadness in Study 2.

Predictor	B	SE B	*z*	*p*	CI LB	CI UB
Order (O)	-0.571	1.009	-0.566	0.571	-2.548	1.406
Economic System Justification (ESJ)	3.177	1.022	3.108	0.002	1.174	5.181
O × ESJ	-1.195	1.027	-1.164	0.245	-3.208	0.818
Homeless Video (H)	67.049	1.626	41.246	0.000	63.863	70.235
O × H	0.180	1.631	0.110	0.912	-3.016	3.376
**ESJ × H**	**-10.027**	**1.691**	**-5.928**	**0.000**	**-13.342**	**-6.712**
O × ESJ × H	-2.724	1.717	-1.587	0.113	-6.089	0.640
Cystic Fibrosis Video (CF)	69.938	1.655	42.262	0.000	66.695	73.182
O × CF	-0.633	1.668	-0.380	0.704	-3.902	2.636
**ESJ × CF**	**-4.498**	**1.759**	**-2.558**	**0.011**	**-7.945**	**-1.051**
O × ESJ × CF	-1.347	1.766	-0.763	0.446	-4.809	2.114
Control Video 1 vs. 2	-0.171	1.073	-0.159	0.873	-2.275	1.932
Homeless Video 1 vs. 4	1.491	2.124	0.702	0.483	-2.672	5.653
Homeless Video 2 vs. 4	-3.023	1.985	-1.522	0.128	-6.914	0.869
Homeless Video 3 vs. 4	-5.061	2.061	-2.456	0.014	-9.101	-1.021
Cystic Fibrosis Video 3 vs. 4	-0.855	1.158	-0.739	0.460	-3.124	1.413
Intercept	8.974	1.003	8.948	0.000	7.008	10.939

Note. Homeless video is coded such that 1 = homeless and 0 = control; cystic fibrosis video is coded such that 1 = cystic fibrosis and 0 = control. Emotions were rated on a 0–100 scale.

Supplementary Table 30. Full regression results for analysis of person-directed pity in Study 2.

Predictor	B	SE B	*z*	*p*	CI LB	CI UB
Order (O)	-0.284	0.993	-0.286	0.775	-2.230	1.662
Economic System Justification (ESJ)	3.749	1.032	3.634	0.000	1.727	5.771
O × ESJ	-0.099	1.032	-0.096	0.924	-2.121	1.923
Homeless Video (H)	60.876	1.750	34.777	0.000	57.445	64.307
O × H	0.546	1.760	0.310	0.757	-2.904	3.996
**ESJ × H**	**-5.789**	**1.782**	**-3.249**	**0.001**	**-9.282**	**-2.297**
O × ESJ × H	-3.631	1.788	-2.030	0.042	-7.136	-0.126
Cystic Fibrosis Video (CF)	58.914	1.850	31.848	0.000	55.288	62.539
O × CF	-1.019	1.860	-0.548	0.584	-4.664	2.626
**ESJ × CF**	**-2.407**	**1.853**	**-1.299**	**0.194**	**-6.040**	**1.225**
O × ESJ × CF	-0.954	1.857	-0.514	0.607	-4.593	2.685
Control Video 1 vs. 2	-0.025	1.152	-0.022	0.983	-2.282	2.232
Homeless Video 1 vs. 4	5.040	2.106	2.394	0.017	0.913	9.166
Homeless Video 2 vs. 4	-6.553	2.252	-2.910	0.004	-10.966	-2.139
Homeless Video 3 vs. 4	-3.600	2.219	-1.622	0.105	-7.950	0.749
Cystic Fibrosis Video 3 vs. 4	-1.380	1.357	-1.017	0.309	-4.041	1.280
Intercept	9.120	0.982	9.288	0.000	7.196	11.045

Note. Homeless video is coded such that 1 = homeless and 0 = control; cystic fibrosis video is coded such that 1 = cystic fibrosis and 0 = control. Emotions were rated on a 0–100 scale.

Supplementary Table 31. Full regression results for analysis of person-directed empathy in Study 2.

Predictor	B	SE B	*z*	*p*	CI LB	CI UB
Order (O)	-0.292	1.525	-0.191	0.848	-3.281	2.698
Economic System Justification (ESJ)	-1.004	1.574	-0.638	0.524	-4.088	2.081
O × ESJ	0.803	1.576	0.509	0.610	-2.286	3.892
Homeless Video (H)	53.946	1.924	28.035	0.000	50.174	57.717
O × H	-0.755	1.923	-0.393	0.694	-4.524	3.013
**ESJ × H**	**-4.987**	**1.959**	**-2.546**	**0.011**	**-8.826**	**-1.148**
O × ESJ × H	-3.781	1.976	-1.913	0.056	-7.655	0.092
Cystic Fibrosis Video (CF)	56.474	1.912	29.537	0.000	52.727	60.221
O × CF	1.199	1.916	0.626	0.531	-2.556	4.954
**ESJ × CF**	**-1.184**	**1.952**	**-0.607**	**0.544**	**-5.011**	**2.642**
O × ESJ × CF	-1.941	1.955	-0.993	0.321	-5.774	1.892
Control Video 1 vs. 2	-3.461	1.542	-2.244	0.025	-6.483	-0.438
Homeless Video 1 vs. 4	-0.818	2.038	-0.401	0.688	-4.811	3.176
Homeless Video 2 vs. 4	0.232	1.790	0.130	0.897	-3.276	3.739
Homeless Video 3 vs. 4	1.169	1.721	0.679	0.497	-2.204	4.542
Cystic Fibrosis Video 3 vs. 4	1.159	1.018	1.139	0.255	-0.836	3.153
Intercept	26.086	1.525	17.102	0.000	23.096	29.075

Note. Homeless video is coded such that 1 = homeless and 0 = control; cystic fibrosis video is coded such that 1 = cystic fibrosis and 0 = control. Emotions were rated on a 0–100 scale.

Supplementary Table 32. Full regression results for analysis of system-directed anger in Study 2.

Predictor	B	SE B	*z*	*p*	CI LB	CI UB
Order (O)	-0.620	1.442	-0.430	0.667	-3.446	2.205
Economic System Justification (ESJ)	-1.649	1.587	-1.039	0.299	-4.760	1.462
O × ESJ	-0.148	1.589	-0.093	0.926	-3.263	2.967
Homeless Video (H)	45.290	1.897	23.880	0.000	41.572	49.007
O × H	0.042	1.906	0.022	0.982	-3.694	3.779
**ESJ × H**	**-12.606**	**1.833**	**-6.879**	**0.000**	**-16.198**	**-9.014**
O × ESJ × H	-1.643	1.843	-0.892	0.373	-5.254	1.969
Cystic Fibrosis Video (CF)	22.919	1.770	12.948	0.000	19.449	26.388
O × CF	5.128	1.782	2.878	0.004	1.636	8.620
**ESJ × CF**	**-5.945**	**1.604**	**-3.706**	**0.000**	**-9.089**	**-2.801**
O × ESJ × CF	-1.927	1.597	-1.207	0.227	-5.057	1.202
Control Video 1 vs. 2	1.425	1.329	1.072	0.284	-1.180	4.029
Homeless Video 1 vs. 4	0.404	2.570	0.157	0.875	-4.633	5.442
Homeless Video 2 vs. 4	3.535	2.390	1.479	0.139	-1.149	8.218
Homeless Video 3 vs. 4	2.333	2.448	0.953	0.341	-2.465	7.131
Cystic Fibrosis Video 3 vs. 4	-1.091	1.589	-0.687	0.492	-4.205	2.023
Intercept	16.964	1.435	11.819	0.000	14.151	19.777

Note. Homeless video is coded such that 1 = homeless and 0 = control; cystic fibrosis video is coded such that 1 = cystic fibrosis and 0 = control. Emotions were rated on a 0–100 scale.

Supplementary Table 33. Full regression results for analysis of system-directed sadness in Study 2.

Predictor	B	SE B	*z*	*p*	CI LB	CI UB
Order (O)	-0.586	1.450	-0.404	0.686	-3.429	2.256
Economic System Justification (ESJ)	-2.310	1.483	-1.557	0.119	-5.217	0.597
O × ESJ	1.303	1.487	0.876	0.381	-1.612	4.217
Homeless Video (H)	48.680	1.907	25.528	0.000	44.943	52.418
O × H	0.798	1.914	0.417	0.677	-2.954	4.549
**ESJ × H**	**-9.729**	**1.935**	**-5.028**	**0.000**	**-13.521**	**-5.936**
O × ESJ × H	-3.037	1.939	-1.567	0.117	-6.837	0.763
Cystic Fibrosis Video (CF)	32.138	1.842	17.451	0.000	28.529	35.748
O × CF	3.104	1.848	1.679	0.093	-0.519	6.726
**ESJ × CF**	**-5.032**	**1.743**	**-2.886**	**0.004**	**-8.448**	**-1.615**
O × ESJ × CF	-1.871	1.736	-1.078	0.281	-5.274	1.532
Control Video 1 vs. 2	1.802	1.357	1.328	0.184	-0.857	4.461
Homeless Video 1 vs. 4	0.810	2.522	0.321	0.748	-4.134	5.754
Homeless Video 2 vs. 4	1.467	2.087	0.703	0.482	-2.623	5.558
Homeless Video 3 vs. 4	0.691	2.259	0.306	0.760	-3.737	5.118
Cystic Fibrosis Video 3 vs. 4	-3.151	1.668	-1.889	0.059	-6.421	0.119
Intercept	18.243	1.442	12.648	0.000	15.416	21.070

Note. Homeless video is coded such that 1 = homeless and 0 = control; cystic fibrosis video is coded such that 1 = cystic fibrosis and 0 = control. Emotions were rated on a 0–100 scale.

Supplementary Table 34. Full regression results for analysis of system-directed disgust in Study 2.

Predictor	B	SE B	*z*	*p*	CI LB	CI UB
Order (O)	-1.427	1.492	-0.956	0.339	-4.352	1.498
Economic System Justification (ESJ)	-2.777	1.697	-1.637	0.102	-6.103	0.548
O × ESJ	0.549	1.637	0.335	0.737	-2.660	3.758
Homeless Video (H)	45.679	1.921	23.774	0.000	41.913	49.445
O × H	0.247	1.934	0.128	0.898	-3.543	4.038
**ESJ × H**	**-12.399**	**1.882**	**-6.587**	**0.000**	**-16.089**	**-8.710**
O × ESJ × H	-2.167	1.882	-1.151	0.250	-5.855	1.521
Cystic Fibrosis Video (CF)	21.865	1.711	12.777	0.000	18.511	25.219
O × CF	6.385	1.719	3.715	0.000	3.016	9.753
**ESJ × CF**	**-5.733**	**1.557**	**-3.682**	**0.000**	**-8.785**	**-2.681**
O × ESJ × CF	-2.835	1.553	-1.825	0.068	-5.880	0.209
Control Video 1 vs. 2	-1.128	1.676	-0.673	0.501	-4.412	2.157
Homeless Video 1 vs. 4	1.223	1.573	0.777	0.437	-1.860	4.306
Homeless Video 2 vs. 4	-3.673	2.138	-1.718	0.086	-7.862	0.517
Homeless Video 3 vs. 4	-0.887	2.118	-0.419	0.675	-5.038	3.263
Cystic Fibrosis Video 3 vs. 4	-3.037	1.653	-1.838	0.066	-6.276	0.201
Intercept	1.157	1.469	0.787	0.431	-1.722	4.036

Note. Homeless video is coded such that 1 = homeless and 0 = control; cystic fibrosis video is coded such that 1 = cystic fibrosis and 0 = control. Emotions were rated on a 0–100 scale.

Supplementary Table 35. Robustness check for analysis of person-directed sadness in Study 2.

Predictor	B	SE B	*z*	*p*	CI LB	CI UB
Order (O)	-0.823	1.001	-0.822	0.411	-2.786	1.140
Economic System Justification (ESJ)	2.978	1.085	2.746	0.006	0.852	5.104
O × ESJ	-1.120	1.071	-1.046	0.296	-3.219	0.979
Homeless Video (H)	66.925	1.620	41.310	0.000	63.749	70.100
O × H	0.405	1.626	0.249	0.803	-2.783	3.593
**ESJ × H**	**-10.354**	**1.745**	**-5.932**	**0.000**	**-13.775**	**-6.933**
O × ESJ × H	-2.728	1.759	-1.551	0.121	-6.175	0.720
Cystic Fibrosis Video (CF)	69.767	1.659	42.059	0.000	66.515	73.018
O × CF	-0.404	1.668	-0.242	0.809	-3.672	2.864
**ESJ × CF**	**-4.550**	**1.807**	**-2.518**	**0.012**	**-8.092**	**-1.008**
O × ESJ × CF	-1.206	1.814	-0.665	0.506	-4.760	2.349
BIDR SDE (SDE)	-3.605	1.044	-3.454	0.001	-5.651	-1.560
O × SDE	-0.456	0.994	-0.459	0.646	-2.404	1.492
H × SDE	2.199	1.785	1.231	0.218	-1.301	5.698
H × O × SDE	-0.250	1.797	-0.139	0.889	-3.772	3.272
BIDR IM (IM)	0.229	1.011	0.227	0.821	-1.752	2.210
O × IM	-0.087	0.967	-0.090	0.928	-1.983	1.809
H × IM	0.472	1.791	0.264	0.792	-3.037	3.982
H × O × IM	-0.220	1.795	-0.122	0.903	-3.739	3.299
D × SDE	0.716	1.812	0.395	0.693	-2.836	4.268
D × O × SDE	-1.450	1.809	-0.801	0.423	-4.996	2.096
D × IM	1.884	1.920	0.982	0.326	-1.878	5.647
D × O × IM	0.244	1.921	0.127	0.899	-3.522	4.010
Control Video 1 vs. 2	0.011	1.066	0.010	0.992	-2.079	2.101
Homeless Video 1 vs. 4	1.692	2.085	0.812	0.417	-2.393	5.778
Homeless Video 2 vs. 4	-3.338	1.975	-1.690	0.091	-7.210	0.533
Homeless Video 3 vs. 4	-5.123	2.119	-2.418	0.016	-9.276	-0.970
Cystic Fibrosis Video 3 vs. 4	-0.683	1.162	-0.588	0.557	-2.961	1.594
Religiosity	1.243	0.423	2.938	0.003	0.414	2.073
Black	6.042	3.067	1.970	0.049	0.032	12.053
Latinx	-1.692	3.463	-0.489	0.625	-8.479	5.095
Asian	-1.284	4.116	-0.312	0.755	-9.351	6.784
Other race	3.225	2.191	1.471	0.141	-1.070	7.520

Age	-0.022	0.074	-0.298	0.765	-0.168	0.124
Gender	-0.123	0.821	-0.150	0.881	-1.732	1.486
Income	0.889	0.489	1.818	0.069	-0.069	1.847
Intercept	2.387	3.159	0.756	0.450	-3.805	8.579

Note. Homeless video is coded such that 1 = homeless and 0 = control; cystic fibrosis video is coded such that 1 = cystic fibrosis and 0 = control. Emotions were rated on a 0–100 scale.

Supplementary Table 36. Robustness check for analysis of person-directed pity in Study 2.

Predictor	B	SE B	*z*	*p*	CI LB	CI UB
Order (O)	-0.264	0.995	-0.265	0.791	-2.215	1.687
Economic System Justification (ESJ)	3.082	1.065	2.894	0.004	0.995	5.170
O × ESJ	-0.242	1.042	-0.232	0.817	-2.284	1.801
Homeless Video (H)	60.803	1.760	34.542	0.000	57.353	64.254
O × H	0.642	1.771	0.363	0.717	-2.829	4.113
**ESJ × H**	**-5.816**	**1.819**	**-3.197**	**0.001**	**-9.381**	**-2.251**
O × ESJ × H	-3.885	1.821	-2.133	0.033	-7.455	-0.315
Cystic Fibrosis Video (CF)	58.725	1.856	31.636	0.000	55.086	62.363
O × CF	-0.807	1.862	-0.433	0.665	-4.457	2.844
**ESJ × CF**	**-2.214**	**1.886**	**-1.174**	**0.240**	**-5.910**	**1.483**
O × ESJ × CF	-1.196	1.888	-0.633	0.526	-4.896	2.504
BIDR SDE (SDE)	-2.306	0.999	-2.309	0.021	-4.264	-0.349
O × SDE	0.300	0.927	0.324	0.746	-1.517	2.117
H × SDE	0.342	1.895	0.181	0.857	-3.373	4.057
H × O × SDE	1.547	1.892	0.818	0.414	-2.162	5.256
BIDR IM (IM)	0.450	1.096	0.410	0.682	-1.699	2.599
O × IM	-0.530	1.049	-0.505	0.613	-2.585	1.525
H × IM	0.775	2.075	0.373	0.709	-3.292	4.842
H × O × IM	-1.444	2.071	-0.697	0.486	-5.503	2.614
D × SDE	-0.509	1.962	-0.259	0.795	-4.355	3.337
D × O × SDE	0.807	1.950	0.414	0.679	-3.016	4.629
D × IM	2.094	2.257	0.928	0.354	-2.330	6.518
D × O × IM	-1.868	2.242	-0.833	0.405	-6.261	2.526
Control Video 1 vs. 2	0.037	1.132	0.033	0.974	-2.181	2.255
Homeless Video 1 vs. 4	4.921	2.076	2.370	0.018	0.852	8.990
Homeless Video 2 vs. 4	-7.142	2.243	-3.185	0.001	-11.538	-2.747
Homeless Video 3 vs. 4	-3.225	2.245	-1.436	0.151	-7.624	1.175
Cystic Fibrosis Video 3 vs. 4	-1.321	1.366	-0.968	0.333	-3.998	1.355
Religiosity	0.891	0.506	1.759	0.079	-0.102	1.883
Black	1.116	3.732	0.299	0.765	-6.199	8.431
Latinx	0.464	2.966	0.157	0.876	-5.348	6.277
Asian	-3.228	4.365	-0.739	0.460	-11.782	5.327
Other race	-0.054	3.728	-0.014	0.989	-7.361	7.254

Age	-0.095	0.088	-1.078	0.281	-0.267	0.078
Gender	-1.134	0.967	-1.172	0.241	-3.030	0.763
Income	1.529	0.532	2.871	0.004	0.485	2.573
Intercept	3.892	3.808	1.022	0.307	-3.571	11.355

Note. Homeless video is coded such that 1 = homeless and 0 = control; cystic fibrosis video is coded such that 1 = cystic fibrosis and 0 = control. Emotions were rated on a 0–100 scale.

Supplementary Table 37. Robustness check for analysis of person-directed empathy in Study 2.

Predictor	B	SE B	*z*	*p*	CI LB	CI UB
Order (O)	-0.468	1.515	-0.309	0.757	-3.438	2.502
Economic System Justification (ESJ)	-1.016	1.565	-0.649	0.516	-4.082	2.051
O × ESJ	0.449	1.545	0.290	0.772	-2.579	3.476
Homeless Video (H)	53.949	1.899	28.408	0.000	50.227	57.672
O × H	-0.631	1.898	-0.333	0.739	-4.351	3.089
**ESJ × H**	**-5.790**	**1.938**	**-2.987**	**0.003**	**-9.589**	**-1.991**
O × ESJ × H	-3.382	1.944	-1.739	0.082	-7.193	0.429
Cystic Fibrosis Video (CF)	56.340	1.897	29.702	0.000	52.622	60.058
O × CF	1.404	1.900	0.739	0.460	-2.320	5.128
**ESJ × CF**	**-1.695**	**1.949**	**-0.870**	**0.384**	**-5.515**	**2.125**
O × ESJ × CF	-1.462	1.948	-0.751	0.453	-5.280	2.355
BIDR SDE (SDE)	-3.602	1.737	-2.074	0.038	-7.007	-0.197
O × SDE	0.782	1.728	0.453	0.651	-2.605	4.170
H × SDE	3.621	2.227	1.626	0.104	-0.744	7.987
H × O × SDE	-1.259	2.245	-0.561	0.575	-5.659	3.142
BIDR IM (IM)	3.078	1.644	1.872	0.061	-0.144	6.300
O × IM	-1.300	1.640	-0.793	0.428	-4.514	1.914
H × IM	-3.186	1.911	-1.667	0.096	-6.932	0.560
H × O × IM	3.083	1.910	1.614	0.107	-0.661	6.827
D × SDE	2.375	2.166	1.096	0.273	-1.871	6.621
D × O × SDE	-2.242	2.159	-1.038	0.299	-6.474	1.990
D × IM	-1.471	1.935	-0.760	0.447	-5.264	2.322
D × O × IM	2.497	1.946	1.283	0.199	-1.317	6.312
Control Video 1 vs. 2	-3.323	1.541	-2.157	0.031	-6.344	-0.303
Homeless Video 1 vs. 4	-0.928	2.019	-0.460	0.646	-4.886	3.029
Homeless Video 2 vs. 4	0.262	1.819	0.144	0.886	-3.304	3.827
Homeless Video 3 vs. 4	1.360	1.742	0.780	0.435	-2.055	4.775
Cystic Fibrosis Video 3 vs. 4	1.397	1.029	1.358	0.174	-0.619	3.414
Religiosity	0.964	0.440	2.193	0.028	0.102	1.826
Black	2.439	2.655	0.919	0.358	-2.765	7.643
Latinx	-1.753	3.412	-0.514	0.607	-8.441	4.934
Asian	-1.682	3.938	-0.427	0.669	-9.401	6.036
Other race	2.191	4.032	0.544	0.587	-5.710	10.093

Age	0.108	0.076	1.419	0.156	-0.041	0.256
Gender	-0.175	0.843	-0.208	0.835	-1.828	1.477
Income	0.545	0.490	1.111	0.267	-0.416	1.506
Intercept	17.641	3.457	5.103	0.000	10.866	24.416

Note. Homeless video is coded such that 1 = homeless and 0 = control; cystic fibrosis video is coded such that 1 = cystic fibrosis and 0 = control. Emotions were rated on a 0–100 scale.

Supplementary Table 38. Robustness check for analysis of system-directed anger in Study 2.

Predictor	B	SE B	*z*	*p*	CI LB	CI UB
Order (O)	-0.826	1.455	-0.568	0.570	-3.677	2.025
Economic System Justification (ESJ)	-2.416	1.706	-1.416	0.157	-5.759	0.927
O × ESJ	0.170	1.635	0.104	0.917	-3.034	3.375
Homeless Video (H)	45.450	1.890	24.052	0.000	41.747	49.154
O × H	-0.335	1.900	-0.176	0.860	-4.060	3.389
**ESJ × H**	**-11.923**	**1.898**	**-6.281**	**0.000**	**-15.643**	**-8.203**
O × ESJ × H	-1.603	1.904	-0.842	0.400	-5.335	2.128
Cystic Fibrosis Video (CF)	22.960	1.763	13.025	0.000	19.505	26.415
O × CF	5.054	1.772	2.852	0.004	1.581	8.527
**ESJ × CF**	**-5.447**	**1.672**	**-3.259**	**0.001**	**-8.724**	**-2.171**
O × ESJ × CF	-2.017	1.662	-1.214	0.225	-5.274	1.240
BIDR SDE (SDE)	-0.201	1.607	-0.125	0.901	-3.350	2.949
O × SDE	0.377	1.520	0.248	0.804	-2.602	3.356
H × SDE	-4.525	2.084	-2.171	0.030	-8.610	-0.440
H × O × SDE	0.028	2.069	0.014	0.989	-4.027	4.083
BIDR IM (IM)	-2.613	1.587	-1.646	0.100	-5.724	0.498
O × IM	0.800	1.412	0.567	0.571	-1.967	3.567
H × IM	0.820	2.190	0.374	0.708	-3.472	5.112
H × O × IM	0.272	2.186	0.125	0.901	-4.012	4.557
D × SDE	-2.812	1.897	-1.483	0.138	-6.530	0.905
D × O × SDE	-0.101	1.908	-0.053	0.958	-3.840	3.638
D × IM	2.417	2.187	1.105	0.269	-1.870	6.703
D × O × IM	-0.162	2.179	-0.074	0.941	-4.433	4.109
Control Video 1 vs. 2	1.390	1.350	1.030	0.303	-1.256	4.036
Homeless Video 1 vs. 4	0.820	2.605	0.315	0.753	-4.286	5.926
Homeless Video 2 vs. 4	2.862	2.305	1.242	0.214	-1.656	7.380
Homeless Video 3 vs. 4	3.111	2.383	1.306	0.192	-1.559	7.781
Cystic Fibrosis Video 3 vs. 4	-1.274	1.604	-0.794	0.427	-4.417	1.869
Religiosity	0.480	0.649	0.739	0.460	-0.792	1.751
Black	1.581	5.329	0.297	0.767	-8.865	12.026
Latinx	6.016	6.143	0.979	0.327	-6.025	18.056
Asian	6.984	4.268	1.636	0.102	-1.381	15.348
Other race	-6.740	5.082	-1.326	0.185	-16.700	3.220

Age	0.011	0.121	0.088	0.930	-0.227	0.248
Gender	0.593	1.199	0.495	0.621	-1.756	2.942
Income	0.432	0.718	0.602	0.547	-0.975	1.839
Intercept	13.385	5.558	2.408	0.016	2.492	24.278

Note. Homeless video is coded such that 1 = homeless and 0 = control; cystic fibrosis video is coded such that 1 = cystic fibrosis and 0 = control. Emotions were rated on a 0–100 scale.

Supplementary Table 39. Robustness check for analysis of system-directed sadness in Study 2.

Predictor	B	SE B	*z*	*p*	CI LB	CI UB
Order (O)	-0.542	1.417	-0.382	0.702	-3.318	2.235
Economic System Justification (ESJ)	-2.690	1.543	-1.744	0.081	-5.714	0.334
O × ESJ	2.022	1.542	1.311	0.190	-1.000	5.045
Homeless Video (H)	49.033	1.871	26.206	0.000	45.366	52.700
O × H	0.026	1.884	0.014	0.989	-3.666	3.719
**ESJ × H**	**-7.374**	**2.159**	**-3.416**	**0.001**	**-11.605**	**-3.142**
O × ESJ × H	-3.881	2.169	-1.789	0.074	-8.133	0.370
Cystic Fibrosis Video (CF)	32.307	1.826	17.694	0.000	28.728	35.885
O × CF	2.368	1.830	1.294	0.196	-1.219	5.955
**ESJ × CF**	**-2.721**	**1.934**	**-1.407**	**0.159**	**-6.513**	**1.070**
O × ESJ × CF	-2.155	1.923	-1.121	0.262	-5.924	1.613
BIDR SDE (SDE)	-0.792	1.427	-0.555	0.579	-3.588	2.005
O × SDE	2.322	1.420	1.636	0.102	-0.460	5.104
H × SDE	7.024	2.013	3.489	0.000	3.078	10.969
H × O × SDE	-2.112	2.007	-1.053	0.292	-6.045	1.821
BIDR IM (IM)	6.992	1.938	3.607	0.000	3.193	10.791
O × IM	-0.391	1.929	-0.203	0.839	-4.172	3.390
H × IM	1.603	1.347	1.190	0.234	-1.037	4.242
H × O × IM	0.931	2.447	0.380	0.704	-3.866	5.727
D × SDE	1.427	2.031	0.703	0.482	-2.553	5.408
D × O × SDE	1.266	2.186	0.579	0.563	-3.018	5.549
D × IM	-3.043	1.650	-1.845	0.065	-6.277	0.190
D × O × IM	17.913	1.409	12.712	0.000	15.151	20.675
Control Video 1 vs. 2	-0.542	1.417	-0.382	0.702	-3.318	2.235
Homeless Video 1 vs. 4	-2.690	1.543	-1.744	0.081	-5.714	0.334
Homeless Video 2 vs. 4	2.022	1.542	1.311	0.190	-1.000	5.045
Homeless Video 3 vs. 4	49.033	1.871	26.206	0.000	45.366	52.700
Cystic Fibrosis Video 3 vs. 4	0.026	1.884	0.014	0.989	-3.666	3.719
Religiosity	-7.374	2.159	-3.416	0.001	-11.605	-3.142
Black	-3.881	2.169	-1.789	0.074	-8.133	0.370
Latinx	32.307	1.826	17.694	0.000	28.728	35.885
Asian	2.368	1.830	1.294	0.196	-1.219	5.955
Other race	-2.721	1.934	-1.407	0.159	-6.513	1.070

Age	-2.155	1.923	-1.121	0.262	-5.924	1.613
Gender	-0.792	1.427	-0.555	0.579	-3.588	2.005
Income	2.322	1.420	1.636	0.102	-0.460	5.104
Intercept	7.024	2.013	3.489	0.000	3.078	10.969

Note. Homeless video is coded such that 1 = homeless and 0 = control; cystic fibrosis video is coded such that 1 = cystic fibrosis and 0 = control. Emotions were rated on a 0–100 scale.

Supplementary Table 40. Robustness check for analysis of system-directed disgust in Study 2.

Predictor	B	SE B	*z*	*p*	CI LB	CI UB
Order (O)	-1.424	1.494	-0.954	0.340	-4.352	1.503
Economic System Justification (ESJ)	-2.841	1.699	-1.672	0.094	-6.171	0.489
O × ESJ	0.502	1.635	0.307	0.759	-2.702	3.706
Homeless Video (H)	45.678	1.921	23.773	0.000	41.912	49.444
O × H	0.243	1.934	0.126	0.900	-3.547	4.033
**ESJ × H**	**-12.403**	**1.882**	**-6.589**	**0.000**	**-16.092**	**-8.713**
O × ESJ × H	-2.161	1.882	-1.149	0.251	-5.849	1.527
Cystic Fibrosis Video (CF)	21.865	1.711	12.777	0.000	18.511	25.219
O × CF	6.384	1.719	3.715	0.000	3.016	9.753
**ESJ × CF**	**-5.733**	**1.557**	**-3.682**	**0.000**	**-8.786**	**-2.681**
O × ESJ × CF	-2.834	1.553	-1.824	0.068	-5.879	0.211
BIDR SDE (SDE)	-1.105	1.675	-0.660	0.509	-4.387	2.178
O × SDE	1.271	1.575	0.807	0.420	-1.816	4.357
H × SDE	-3.671	2.138	-1.717	0.086	-7.861	0.519
H × O × SDE	-0.883	2.118	-0.417	0.677	-5.033	3.268
BIDR IM (IM)	-3.016	1.663	-1.814	0.070	-6.275	0.243
O × IM	1.051	1.484	0.708	0.479	-1.857	3.959
H × IM	2.177	2.274	0.957	0.338	-2.280	6.635
H × O × IM	0.219	2.261	0.097	0.923	-4.214	4.651
D × SDE	-2.522	1.787	-1.411	0.158	-6.025	0.980
D × O × SDE	-0.832	1.793	-0.464	0.643	-4.347	2.683
D × IM	2.647	2.163	1.224	0.221	-1.592	6.886
D × O × IM	0.505	2.159	0.234	0.815	-3.726	4.737
Control Video 1 vs. 2	1.814	1.365	1.329	0.184	-0.862	4.489
Homeless Video 1 vs. 4	2.903	2.606	1.114	0.265	-2.204	8.010
Homeless Video 2 vs. 4	3.568	2.402	1.485	0.137	-1.140	8.275
Homeless Video 3 vs. 4	-1.214	2.326	-0.522	0.602	-5.773	3.346
Cystic Fibrosis Video 3 vs. 4	-2.039	1.514	-1.346	0.178	-5.007	0.930
Religiosity	0.516	0.667	0.773	0.439	-0.792	1.824
Black	2.705	5.380	0.503	0.615	-7.839	13.250
Latinx	6.095	6.206	0.982	0.326	-6.069	18.259
Asian	9.602	4.268	2.250	0.024	1.237	17.966
Other race	-9.833	4.619	-2.129	0.033	-18.886	-0.780

Age	0.016	0.123	0.130	0.897	-0.226	0.258
Gender	-0.138	1.220	-0.113	0.910	-2.530	2.254
Income	0.051	0.755	0.067	0.947	-1.430	1.531
Intercept	15.906	5.768	2.758	0.006	4.601	27.211

Note. Homeless video is coded such that 1 = homeless and 0 = control; cystic fibrosis video is coded such that 1 = cystic fibrosis and 0 = control. Emotions were rated on a 0–100 scale.

Descriptive Statistics and Correlations (Study 3)

Supplementary Table 41. Variables assessed in Study 3 (homeless condition).

Measure	M	SD	*Corrugator*	*Levator*	SCL	Affect
Corrugator	1.26	2.54	—	0.22	0.15	-0.25
Levator	0.78	3.06		—	0.41**	-0.20
SCL	-0.18	1.20			—	-0.20
Positive Affect	37.95	21.71				—

Note. ** $p < .01$. *Corrugator* and *levator* reflect difference from baseline in microvolts; SCL reflects difference from baseline in microsiemens. Positive affect (slider) ratings range from 0 to 100. Correlations are based on within-participant means.

Supplementary Table 42. Variables assessed in Study 3 (control condition).

Measure	M	SD	*Corrugator*	*Levator*	SCL	Affect
Corrugator	0.07	2.42	—	0.08	-0.08	-0.06
Levator	0.02	1.56		—	0.16	0.22
SCL	-0.34	1.38			—	-0.09
Positive Affect	54.99	11.17				—

Note. *Corrugator* and *levator* reflect difference from baseline in microvolts; SCL reflects difference from baseline in microsiemens. Positive affect (slider) ratings range from 0 to 100. Correlations are based on within-participant means.

Full Regression Results (Study 3)

Supplementary Table 43. Full regression results for analysis of *corrugator* activity in Study 3.

Predictor	B	SE B	*z*	*p*	CI LB	CI UB
Economic System Justification (ESJ)	0.467	0.380	1.227	0.220	-0.279	1.212
Video Type (V)	1.368	0.671	2.039	0.041	0.053	2.683
**ESJ × V**	**-1.507**	**0.736**	**-2.046**	**0.041**	**-2.950**	**-0.063**
Control Video 1 vs. 2	0.042	0.364	0.115	0.909	-0.672	0.755
Homeless Video 1 vs. 4	0.070	0.682	0.102	0.919	-1.267	1.406
Homeless Video 2 vs. 4	-0.680	0.777	-0.875	0.381	-2.203	0.843
Homeless Video 3 vs. 4	-0.125	0.683	-0.183	0.855	-1.463	1.213
Intercept	-0.074	0.428	-0.173	0.863	-0.913	0.765

Note. Video type is coded such that 1 = homeless and 0 = control. *Corrugator* activity reflects difference from baseline in microvolts

Supplementary Table 44. Full regression results for analysis of *levator* activity in Study 3.

Predictor	B	SE B	*z*	*p*	CI LB	CI UB
Economic System Justification (ESJ)	0.676	0.500	1.350	0.177	-0.305	1.656
Video Type (V)	0.781	0.549	1.422	0.155	-0.295	1.857
**ESJ × V**	**-2.458**	**1.572**	**-1.564**	**0.118**	**-5.538**	**0.622**
Control Video 1 vs. 2	0.113	0.141	0.801	0.423	-0.164	0.390
Homeless Video 1 vs. 4	0.531	0.467	1.138	0.255	-0.383	1.446
Homeless Video 2 vs. 4	-0.399	0.535	-0.746	0.456	-1.448	0.649
Homeless Video 3 vs. 4	-0.524	0.484	-1.083	0.279	-1.472	0.424
Intercept	0.004	0.204	0.019	0.985	-0.397	0.404

Note. Video type is coded such that 1 = homeless and 0 = control. *Levator* activity reflects difference from baseline in microvolts

Supplementary Table 45. Full regression results for analysis of skin conductance level in Study 3.

Predictor	B	SE B	*z*	*p*	CI LB	CI UB
Economic System Justification (ESJ)	0.125	0.172	0.727	0.467	-0.212	0.461
Video Type (V)	0.399	0.197	2.028	0.043	0.013	0.784
**ESJ × V**	**-0.567**	**0.256**	**-2.217**	**0.027**	**-1.069**	**-0.066**
Control Video 1 vs. 2	0.384	0.183	2.092	0.036	0.024	0.743
Homeless Video 1 vs. 4	0.144	0.215	0.671	0.502	-0.277	0.566
Homeless Video 2 vs. 4	-0.144	0.197	-0.728	0.467	-0.530	0.243
Homeless Video 3 vs. 4	-0.187	0.180	-1.040	0.298	-0.540	0.166
Intercept	-0.399	0.211	-1.888	0.059	-0.813	0.015

Note. Video type is coded such that 1 = homeless and 0 = control. Skin conductance level reflects difference from baseline in microsiemens.

Supplementary Table 46. Full regression results for analysis of self-reported positive affect in Study 3.

Predictor	B	SE B	*z*	*p*	CI LB	CI UB
Economic System Justification (ESJ)	1.019	1.235	0.825	0.409	-1.401	3.439
Video Type (V)	-15.157	2.841	-5.334	0.000	-20.726	-9.588
**ESJ × V**	**4.693**	**2.390**	**1.963**	**0.050**	**0.008**	**9.378**
Control Video 1 vs. 2	-3.594	1.795	-2.002	0.045	-7.111	-0.076
Homeless Video 1 vs. 4	-3.741	6.762	-0.553	0.580	-16.994	9.513
Homeless Video 2 vs. 4	10.151	2.973	3.415	0.001	4.325	15.978
Homeless Video 3 vs. 4	-6.345	4.665	-1.360	0.174	-15.488	2.799
Intercept	55.829	1.769	31.559	0.000	52.362	59.296

Note. Video type is coded such that 1 = homeless and 0 = control. Positive affect (slider) ratings range from 0 to 100.

Descriptive Statistics and Correlations (Study 4)

Supplementary Table 47. Variables assessed in Study 4 (homeless condition).

Measure	M	SD	*Corrugator*	*Levator*	Affect
Corrugator	1.26	2.54	—	0.03	-0.46 **
Levator	0.78	3.06		—	-0.07
Positive Affect	37.95	21.71			—

Note. ** $p < .01$. *Corrugator* and *levator* reflect difference from baseline in microvolts. Positive affect (slider) ratings range from 0 to 100. Correlations are based on within-participant means.

Supplementary Table 48. Variables assessed in Study 4 (control condition).

Measure	M	SD	*Corrugator*	*Levator*	Affect
Corrugator	1.26	2.54	—	0.02	0.10
Levator	0.78	3.06		—	-0.09
Positive Affect	37.95	21.71			—

Note. *Corrugator* and *levator* reflect difference from baseline in microvolts. Positive affect (slider) ratings range from 0 to 100. Correlations are based on within-participant means.

Full Regression Results (Study 4)

Supplementary Table 49. Full regression results for analysis of *corrugator* activity in Study 4.

Predictor	B	SE B	*z*	*p*	CI LB	CI UB
Economic System Justification (ESJ)	0.688	0.428	1.606	0.108	-0.152	1.528
Video Type (V)	3.895	0.853	4.568	0.000	2.224	5.567
**ESJ × V**	**-1.735**	**0.742**	**-2.337**	**0.019**	**-3.190**	**-0.280**
Control Video 1 vs. 2	0.168	0.420	0.399	0.690	-0.655	0.990
Homeless Video 1 vs. 4	0.501	1.748	0.287	0.774	-2.925	3.927
Homeless Video 2 vs. 4	-0.099	1.179	-0.084	0.933	-2.410	2.212
Homeless Video 3 vs. 4	0.531	1.259	0.422	0.673	-1.936	2.998
Intercept	1.264	0.385	3.281	0.001	0.509	2.020

Note. Video type is coded such that 1 = homeless and 0 = control. *Corrugator* activity reflects difference from baseline in microvolts

Supplementary Table 50. Full regression results for analysis of *levator* activity in Study 4.

Predictor	B	SE B	*z*	*p*	CI LB	CI UB
Economic System Justification (ESJ)	-0.475	0.276	-1.723	0.085	-1.015	0.065
Video Type (V)	1.645	0.655	2.512	0.012	0.361	2.928
**ESJ × V**	**-0.281**	**0.631**	**-0.445**	**0.656**	**-1.518**	**0.956**
Control Video 1 vs. 2	0.049	0.284	0.173	0.863	-0.507	0.605
Homeless Video 1 vs. 4	-0.150	0.942	-0.159	0.874	-1.996	1.696
Homeless Video 2 vs. 4	1.068	1.322	0.808	0.419	-1.523	3.659
Homeless Video 3 vs. 4	-0.894	0.822	-1.087	0.277	-2.506	0.718
Intercept	1.201	0.269	4.473	0.000	0.675	1.727

Note. Video type is coded such that 1 = homeless and 0 = control. *Levator* activity reflects difference from baseline in microvolts

Supplementary Table 51. Full regression results for analysis of self-reported positive affect in Study 4.

Predictor	B	SE B	*z*	*p*	CI LB	CI UB
Economic System Justification (ESJ)	2.688	3.917	0.686	0.492	-4.988	10.365
Video Type (V)	-9.656	3.579	-2.698	0.007	-16.670	-2.642
**ESJ × V**	**-0.176**	**4.442**	**-0.040**	**0.968**	**-8.882**	**8.529**
Control Video 1 vs. 2	-4.493	3.400	-1.322	0.186	-11.157	2.171
Homeless Video 1 vs. 4	6.641	4.348	1.527	0.127	-1.882	15.164
Homeless Video 2 vs. 4	-2.422	5.109	-0.474	0.635	-12.436	7.592
Homeless Video 3 vs. 4	-3.139	4.512	-0.696	0.487	-11.982	5.703
Intercept	45.846	3.452	13.281	0.000	39.080	52.611

Note. Video type is coded such that 1 = homeless and 0 = control. Positive affect (slider) ratings range from 0 to 100.

Descriptive Statistics and Correlations (Study 5)

Supplementary Table 52. Variables assessed in Study 5 (homeless condition).

Measure	M	SD	*Corrugator*	*Levator*	SCL	Affect
Corrugator	5.73	5.40	—	0.03	-0.03	0.17
Levator	1.91	5.98		—	0.03	0.10
SCL	-0.48	1.28			—	0.09
Positive Affect	43.81	16.89				—

Note. *Corrugator* and *levator* reflect difference from baseline in microvolts; SCL reflects difference from baseline in microsiemens. Positive affect (slider) ratings range from 0 to 100. Correlations are based on within-participant means.

Supplementary Table 53. Variables assessed in Study 5 (cystic fibrosis condition).

Measure	M	SD	*Corrugator*	*Levator*	SCL	Affect
Corrugator	3.68	4.43	—	0.16 †	-0.22	0.13
Levator	0.46	3.83		—	-0.10	-0.02
SCL	0.40	1.49			—	-0.07
Positive Affect	42.08	17.22				—

Note. † $p < .10$. *Corrugator* and *levator* reflect difference from baseline in microvolts; SCL reflects difference from baseline in microsiemens. Positive affect (slider) ratings range from 0 to 100. Correlations are based on within-participant means.

Supplementary Table 54. Variables assessed in Study 5 (control condition).

Measure	M	SD	*Corrugator*	*Levator*	SCL	Affect
Corrugator	1.91	3.14	—	0.13	0.04	-0.01
Levator	0.82	1.71		—	0.02	0.10
SCL	-0.54	1.14			—	-0.09
Positive Affect	50.38	18.16				—

Note. *Corrugator* and *levator* reflect difference from baseline in microvolts; SCL reflects difference from baseline in microsiemens. Positive affect (slider) ratings range from 0 to 100. Correlations are based on within-participant means.

Full Regression Results (Study 5)

Supplementary Table 55. Full regression results for analysis of *corrugator* activity in Study 5.

Predictor	B	SE B	*z*	*p*	CI LB	CI UB
Order (O)	-0.361	0.350	-1.032	0.302	-1.046	0.324
Economic System Justification (ESJ)	0.104	0.474	0.219	0.826	-0.824	1.032
O × ESJ	0.746	0.476	1.568	0.117	-0.186	1.678
Homeless Video (H)	3.245	0.438	7.407	0.000	2.386	4.103
O × H	-0.238	0.427	-0.557	0.578	-1.075	0.599
**ESJ × H**	**-0.895**	**0.486**	**-1.842**	**0.066**	**-1.847**	**0.057**
O × ESJ × H	-0.637	0.492	-1.296	0.195	-1.601	0.326
Cystic Fibrosis Video (C)	1.925	0.621	3.098	0.002	0.707	3.142
O × C	0.303	0.631	0.480	0.631	-0.934	1.540
**ESJ × C**	**-0.912**	**0.690**	**-1.320**	**0.187**	**-2.265**	**0.442**
O × ESJ × C	-0.823	0.733	-1.123	0.261	-2.259	0.613
Control Video 1 vs. 2	-0.387	0.364	-1.065	0.287	-1.100	0.326
Homeless Video 1 vs. 4	-1.410	0.613	-2.300	0.021	-2.611	-0.209
Homeless Video 2 vs. 4	0.532	0.823	0.646	0.518	-1.082	2.145
Homeless Video 3 vs. 4	-0.102	0.774	-0.132	0.895	-1.620	1.415
Cystic Fibrosis Video 3 vs. 4	0.103	0.508	0.204	0.839	-0.891	1.098
Intercept	1.685	0.343	4.910	0.000	1.012	2.358

Note. Homeless video is coded such that 1 = homeless and 0 = control; cystic fibrosis video is coded such that 1 = cystic fibrosis and 0 = control. *Corrugator* activity reflects difference from baseline in microvolts.

Supplementary Table 56. Full regression results for analysis of *levator* activity in Study 5.

Predictor	B	SE B	*z*	*p*	CI LB	CI UB
Order (O)	-0.352	0.169	-2.080	0.038	-0.683	-0.020
Economic System Justification (ESJ)	-0.277	0.207	-1.341	0.180	-0.683	0.128
O × ESJ	0.107	0.202	0.532	0.594	-0.288	0.503
Homeless Video (H)	0.893	0.516	1.729	0.084	-0.119	1.904
O × H	0.914	0.503	1.816	0.069	-0.072	1.901
**ESJ × H**	**1.171**	**1.132**	**1.034**	**0.301**	**-1.048**	**3.390**
O × ESJ × H	1.012	1.182	0.856	0.392	-1.305	3.329
Cystic Fibrosis Video (C)	-0.117	0.445	-0.262	0.793	-0.988	0.755
O × C	0.269	0.435	0.619	0.536	-0.583	1.121
**ESJ × C**	**-1.045**	**0.864**	**-1.210**	**0.226**	**-2.739**	**0.648**
O × ESJ × C	-1.123	0.900	-1.249	0.212	-2.887	0.640
Control Video 1 vs. 2	-0.035	0.191	-0.184	0.854	-0.410	0.340
Homeless Video 1 vs. 4	-0.205	0.623	-0.328	0.743	-1.425	1.016
Homeless Video 2 vs. 4	-1.118	0.708	-1.580	0.114	-2.505	0.269
Homeless Video 3 vs. 4	1.051	0.949	1.108	0.268	-0.808	2.910
Cystic Fibrosis Video 3 vs. 4	0.755	0.469	1.609	0.108	-0.165	1.675
Intercept	0.721	0.168	4.283	0.000	0.391	1.051

Note. Homeless video is coded such that 1 = homeless and 0 = control; cystic fibrosis video is coded such that 1 = cystic fibrosis and 0 = control. *Levator* activity reflects difference from baseline in microvolts.

Supplementary Table 57. Full regression results for analysis of skin conductance level in Study 5.

Predictor	B	SE B	*z*	*p*	CI LB	CI UB
Order (O)	-0.356	0.144	-2.469	0.014	-0.639	-0.073
Economic System Justification (ESJ)	0.221	0.172	1.287	0.198	-0.116	0.558
O × ESJ	0.039	0.168	0.230	0.818	-0.290	0.367
Homeless Video (H)	0.543	0.171	3.170	0.002	0.207	0.879
O × H	0.300	0.171	1.756	0.079	-0.035	0.634
**ESJ × H**	**-0.449**	**0.211**	**-2.124**	**0.034**	**-0.864**	**-0.035**
O × ESJ × H	-0.041	0.212	-0.193	0.847	-0.456	0.375
Cystic Fibrosis Video (C)	1.284	0.299	4.301	0.000	0.699	1.869
O × C	0.796	0.296	2.687	0.007	0.215	1.376
**ESJ × C**	**-0.547**	**0.328**	**-1.669**	**0.095**	**-1.190**	**0.095**
O × ESJ × C	-0.223	0.318	-0.703	0.482	-0.846	0.399
Control Video 1 vs. 2	0.087	0.122	0.711	0.477	-0.153	0.327
Homeless Video 1 vs. 4	-0.254	0.185	-1.375	0.169	-0.615	0.108
Homeless Video 2 vs. 4	-0.435	0.141	-3.091	0.002	-0.712	-0.159
Homeless Video 3 vs. 4	0.513	0.176	2.922	0.003	0.169	0.857
Cystic Fibrosis Video 3 vs. 4	-0.390	0.152	-2.576	0.010	-0.687	-0.093
Intercept	-0.638	0.144	-4.421	0.000	-0.921	-0.355

Note. Homeless video is coded such that 1 = homeless and 0 = control; cystic fibrosis video is coded such that 1 = cystic fibrosis and 0 = control. Skin conductance level reflects difference from baseline in microsiemens.

Supplementary Table 58. Full regression results for analysis of self-reported positive affect in Study 5.

Predictor	B	SE B	*z*	*p*	CI LB	CI UB
Order (O)	-0.713	2.231	-0.319	0.749	-5.086	3.660
Economic System Justification (ESJ)	2.136	1.737	1.230	0.219	-1.268	5.540
O × ESJ	-0.469	1.705	-0.275	0.783	-3.810	2.872
Homeless Video (H)	-7.407	1.352	-5.480	0.000	-10.056	-4.758
O × H	-2.940	1.325	-2.219	0.027	-5.538	-0.343
**ESJ × H**	**0.239**	**1.639**	**0.146**	**0.884**	**-2.973**	**3.452**
O × ESJ × H	1.845	1.546	1.194	0.233	-1.185	4.875
Cystic Fibrosis Video (C)	-8.262	2.176	-3.798	0.000	-12.527	-3.998
O × C	-1.215	2.172	-0.559	0.576	-5.471	3.042
ESJ × C	**0.245**	**2.102**	**0.117**	**0.907**	**-3.876**	**4.366**
O × ESJ × C	-0.964	2.036	-0.473	0.636	-4.955	3.027
Control Video 1 vs. 2	-2.948	2.024	-1.456	0.145	-6.915	1.019
Homeless Video 1 vs. 4	1.067	2.202	0.485	0.628	-3.249	5.384
Homeless Video 2 vs. 4	-2.210	2.350	-0.940	0.347	-6.815	2.396
Homeless Video 3 vs. 4	-0.165	2.292	-0.072	0.942	-4.658	4.327
Cystic Fibrosis Video 3 vs. 4	-0.585	1.903	-0.307	0.759	-4.314	3.145
Intercept	50.312	2.234	22.520	0.000	45.933	54.691

Note. Homeless video is coded such that 1 = homeless and 0 = control; cystic fibrosis video is coded such that 1 = cystic fibrosis and 0 = control. Positive affect (slider) ratings range from 0 to 100.

Full Regression Results (Integrative Data Analysis)

Supplementary Table 59. Full regression results for analysis of *corrugator* activity in the IDA.

Predictor	B	SE B	*z*	*p*	CI LB	CI UB
Study 1 (E1)	-1.097	0.311	-3.533	0.000	-1.706	-0.488
Economic System Justification (ESJ)	0.304	0.216	1.403	0.161	-0.121	0.728
E1 × ESJ	0.186	0.331	0.561	0.575	-0.463	0.835
Video Type (V)	2.831	0.389	7.283	0.000	2.069	3.592
E1 × V	-1.488	0.535	-2.779	0.005	-2.537	-0.438
**ESJ × V**	**-1.192**	**0.355**	**-3.356**	**0.001**	**-1.889**	**-0.496**
E1 × ESJ × V	-0.116	0.588	-0.197	0.844	-1.269	1.037
Study 2 (E2)	0.224	0.312	0.717	0.474	-0.388	0.835
E2 × ESJ	0.447	0.328	1.363	0.173	-0.196	1.090
E2 × B	1.091	0.629	1.735	0.083	-0.141	2.324
E2 × ESJ × V	-0.510	0.542	-0.940	0.347	-1.572	0.553
Control Video 1 vs. 2	-0.058	0.235	-0.246	0.806	-0.517	0.402
Homeless Video 1 vs. 4	-0.708	0.611	-1.159	0.247	-1.905	0.490
Homeless Video 2 vs. 4	0.454	0.597	0.761	0.447	-0.715	1.623
Homeless Video 3 vs. 4	-0.150	0.586	-0.256	0.798	-1.298	0.998
Intercept	1.047	0.219	4.777	0.000	0.617	1.476

Note. Video type is coded such that 1 = homeless and 0 = control. Study 1 and Study 2 are deviation contrasts with Study 3 as the reference category. *Corrugator* activity reflects difference from baseline in microvolts.

Supplementary Table 60. Full regression results for analysis of *levator* activity in the IDA.

Predictor	B	SE B	*z*	*p*	CI LB	CI UB
Study 1 (E1)	-0.665	0.178	-3.728	0.000	-1.015	-0.316
Economic System Justification (ESJ)	-0.053	0.213	-0.251	0.802	-0.471	0.364
E1 × ESJ	0.746	0.357	2.087	0.037	0.045	1.446
Video Type (V)	1.077	0.347	3.104	0.002	0.397	1.757
E1 × V	-0.258	0.459	-0.562	0.574	-1.158	0.642
**ESJ × V**	**-0.651**	**0.588**	**-1.107**	**0.268**	**-1.805**	**0.502**
E1 × ESJ × V	-1.689	1.101	-1.534	0.125	-3.847	0.469
Study 2 (E2)	0.515	0.200	2.580	0.010	0.124	0.906
E2 × ESJ	-0.419	0.258	-1.625	0.104	-0.924	0.086
E2 × B	0.570	0.519	1.098	0.272	-0.447	1.586
E2 × ESJ × V	0.112	0.757	0.149	0.882	-1.371	1.596
Control Video 1 vs. 2	0.041	0.127	0.322	0.748	-0.209	0.290
Homeless Video 1 vs. 4	-0.447	0.417	-1.073	0.283	-1.264	0.370
Homeless Video 2 vs. 4	-0.282	0.455	-0.620	0.535	-1.173	0.609
Homeless Video 3 vs. 4	0.630	0.782	0.805	0.421	-0.904	2.163
Intercept	0.686	0.133	5.173	0.000	0.426	0.946

Note. Video type is coded such that 1 = homeless and 0 = control. Study 1 and Study 2 are deviation contrasts with Study 3 as the reference category. *Levator* activity reflects difference from baseline in microvolts.

Supplementary Table 61. Full regression results for analysis of skin conductance level in the IDA.

Predictor	B	SE B	*z*	*p*	CI LB	CI UB
Study 1 (E1)	-0.081	0.121	-0.667	0.505	-0.318	0.156
Economic System Justification (ESJ)	0.156	0.103	1.523	0.128	-0.045	0.358
E1 × ESJ	-0.010	0.106	-0.098	0.922	-0.219	0.198
Video Type (V)	0.406	0.123	3.302	0.001	0.165	0.648
E1 × V	0.030	0.122	0.247	0.805	-0.210	0.270
**ESJ × V**	**-0.470**	**0.145**	**-3.229**	**0.001**	**-0.755**	**-0.185**
E1 × ESJ × V	0.125	0.142	0.882	0.378	-0.153	0.403
Control Video 1 vs. 2	0.202	0.109	1.842	0.065	-0.013	0.416
Homeless Video 1 vs. 4	-0.121	0.132	-0.912	0.362	-0.380	0.139
Homeless Video 2 vs. 4	-0.301	0.115	-2.610	0.009	-0.527	-0.075
Homeless Video 3 vs. 4	0.245	0.152	1.617	0.106	-0.052	0.543
Intercept	-0.436	0.120	-3.621	0.000	-0.672	-0.200

Note. Video type is coded such that 1 = homeless and 0 = control. Study 1 and Study 2 are deviation contrasts with Study 3 as the reference category. Skin conductance level reflects difference from baseline in microsiemens.

Robustness Checks (Integrative Data Analysis)

Supplementary Table 63. Robustness check for analysis of *corrugator* activity in the IDA.

Predictor	B	SE B	*z*	*p*	CI LB	CI UB
Study 1 (E1)	-1.104	0.333	-3.316	0.001	-1.757	-0.452
Economic System Justification (ESJ)	0.527	0.237	2.226	0.026	0.063	0.991
E1 × ESJ	0.204	0.353	0.578	0.563	-0.488	0.896
Video Type (V)	2.848	0.392	7.273	0.000	2.080	3.615
E1 × V	-1.506	0.537	-2.805	0.005	-2.558	-0.454
**ESJ × V**	**-1.201**	**0.361**	**-3.322**	**0.001**	**-1.909**	**-0.492**
E1 × ESJ × V	-0.134	0.592	-0.227	0.820	-1.295	1.026
Study 2 (E2)	0.252	0.328	0.768	0.442	-0.391	0.894
E2 × ESJ	0.455	0.325	1.401	0.161	-0.181	1.091
E2 × B	1.072	0.631	1.698	0.090	-0.166	2.310
E2 × ESJ × V	-0.519	0.546	-0.950	0.342	-1.589	0.551
Control Video 1 vs. 2	-0.083	0.234	-0.354	0.723	-0.541	0.375
Homeless Video 1 vs. 4	-0.657	0.592	-1.110	0.267	-1.818	0.503
Homeless Video 2 vs. 4	0.313	0.579	0.541	0.589	-0.822	1.448
Homeless Video 3 vs. 4	-0.049	0.587	-0.084	0.933	-1.199	1.100
Religiosity	0.048	0.227	0.212	0.832	-0.397	0.493
Black	-0.051	1.021	-0.050	0.961	-2.052	1.951
Latinx	-0.080	0.618	-0.129	0.897	-1.290	1.131
Asian	-0.357	0.528	-0.677	0.498	-1.391	0.677
Other race	1.358	0.961	1.413	0.158	-0.526	3.242
Age	0.183	0.160	1.141	0.254	-0.131	0.497
Gender	0.557	0.220	2.535	0.011	0.126	0.987
Parents' income	-0.123	0.221	-0.556	0.578	-0.555	0.310
Intercept	0.883	0.350	2.525	0.012	0.198	1.568

Note. Video type is coded such that 1 = homeless and 0 = control. Study 2 and Study 3 are deviation contrasts with Study 4 as the reference category. Gender is coded such that -1 = male and 1 = female. Participant race (Black, Latinx, Asian, Other race) is dummy-coded with White as the reference category. *Corrugator* activity reflects difference from baseline in microvolts.

Supplementary Table 64. Robustness check for analysis of skin conductance level in the IDA.

Predictor	B	SE B	*z*	*p*	CI LB	CI UB
Study 1 (E1)	-0.087	0.125	-0.691	0.490	-0.332	0.159
Economic System Justification (ESJ)	0.192	0.111	1.735	0.083	-0.025	0.409
E1 × ESJ	-0.001	0.109	-0.006	0.995	-0.214	0.213
Video Type (V)	0.424	0.124	3.423	0.001	0.181	0.667
E1 × V	0.045	0.123	0.369	0.712	-0.195	0.286
**ESJ × V**	**-0.464**	**0.144**	**-3.225**	**0.001**	**-0.747**	**-0.182**
E1 × ESJ × V	0.137	0.141	0.973	0.331	-0.139	0.413
Control Video 1 vs. 2	0.217	0.112	1.936	0.053	-0.003	0.436
Homeless Video 1 vs. 4	-0.068	0.138	-0.488	0.625	-0.339	0.204
Homeless Video 2 vs. 4	-0.337	0.107	-3.149	0.002	-0.547	-0.127
Homeless Video 3 vs. 4	0.232	0.139	1.666	0.096	-0.041	0.505
Religiosity	0.039	0.067	0.580	0.562	-0.092	0.170
Black	0.506	0.235	2.149	0.032	0.045	0.968
Latinx	0.241	0.253	0.953	0.341	-0.255	0.737
Asian	0.107	0.204	0.526	0.599	-0.292	0.506
Other race	0.578	0.360	1.608	0.108	-0.127	1.283
Age	-0.037	0.059	-0.620	0.535	-0.153	0.079
Gender	0.173	0.099	1.760	0.078	-0.020	0.366
Parents’ income	0.025	0.077	0.332	0.740	-0.125	0.176
Intercept	-0.638	0.168	-3.791	0.000	-0.968	-0.308

Note. Video type is coded such that 1 = homeless and 0 = control. Study 2 and Study 3 are deviation contrasts with Study 4 as the reference category. Gender is coded such that -1 = male and 1 = female. Participant race (Black, Latinx, Asian, Other race) is dummy-coded with White as the reference category. Skin conductance level reflects difference from baseline in microsiemens.

Descriptive Statistics and Correlations (Study 6)

Supplementary Table 65. Emotions following poor encounter in Study 6.

Emotion	M	SD	Empathy (person)	Anger (person)	Sadness (person)	Anger (system)	Disgust (system)
Empathy (person)	4.74	1.62	—	-0.064	0.712 ***	0.536 ***	0.525 ***
Anger (person)	1.60	1.10		—	-0.032	-0.186 *	0.198 **
Sadness (person)	4.77	1.64			—	0.198 **	0.519 ***
Anger (system)	3.54	1.84				—	0.891 ***
Disgust (system)	3.49	1.88					—

Note. * p < .05, ** p < .01, *** p < .001. Emotion was rated on a 1 (not at all) to 7 (extremely) scale.

Supplementary Table 66. Emotions following rich encounter in Study 6.

Emotion	M	SD	Empathy (person)	Anger (person)	Sadness (self)	Jealousy	Anger (system)	Disgust (system)
Empathy (person)	2.44	1.48	—	-0.272 ***	0.126	0.091	-0.111	-0.090
Anger (person)	2.34	1.73		—	-0.0797	0.158 *	0.552 ***	0.612 ***
Sadness (self)	2.41	1.51			—	0.658 ***	0.349 ***	0.275 ***
jealousy	3.28	1.74				—	0.891 ***	0.319 ***
Anger (system)	2.65	1.75					—	0.882 ***
Disgust (system)	2.67	1.79						—

Note. * p < .05, *** p < .001. Emotion was rated on a 1 (not at all) to 7 (extremely) scale.

Full Regression Results (Study 6)

Supplementary Table 67. Full regression results for analysis of person-directed empathy after poor encounter in Study 6.

Predictor	B	SE B	*z*	*p*	CI LB	CI UB
Econ. System Justification (ESJ)	-0.288	0.163	-1.774	0.076	-0.607	0.030
Within Poor Encounter (WP)	1.979	0.186	10.661	0.000	1.616	2.343
**WP × ESJ**	**-0.015**	**0.185**	**-0.079**	**0.937**	**-0.376**	**0.347**
Between Poor Encounter (BP)	-0.126	0.139	-0.910	0.363	-0.398	0.146
BP × ESJ	-0.155	0.135	-1.146	0.252	-0.419	0.110
Intercept	2.803	0.162	17.351	0.000	2.487	3.120

Note. WP is coded such that 1 = Encounter and 0 = No Encounter. Emotion was rated on a 1 (not at all) to 7 (extremely) scale.

Supplementary Table 68. Full regression results for analysis of person-directed anger after poor encounter in Study 6.

Predictor	B	SE B	*z*	*p*	CI LB	CI UB
Econ. System Justification (ESJ)	0.224	0.116	1.938	0.053	-0.003	0.451
Within Poor Encounter (WP)	0.114	0.112	1.020	0.308	-0.105	0.332
**WP × ESJ**	**-0.302**	**0.111**	**-2.723**	**0.006**	**-0.519**	**-0.085**
Between Poor Encounter (BP)	-0.049	0.103	-0.478	0.633	-0.252	0.153
BP × ESJ	0.031	0.101	0.313	0.754	-0.166	0.229
Intercept	1.554	0.115	13.525	0.000	1.329	1.779

Note. WP is coded such that 1 = Encounter and 0 = No Encounter. Emotion was rated on a 1 (not at all) to 7 (extremely) scale.

Supplementary Table 69. Full regression results for analysis of person-directed sadness after poor encounter in Study 6.

Predictor	B	SE B	*z*	*p*	CI LB	CI UB
Econ. System Justification (ESJ)	-0.256	0.156	-1.644	0.100	-0.562	0.049
Within Poor Encounter (WP)	2.043	0.191	10.706	0.000	1.669	2.417
**WP × ESJ**	**-0.026**	**0.190**	**-0.139**	**0.890**	**-0.398**	**0.346**
Between Poor Encounter (BP)	-0.063	0.136	-0.460	0.645	-0.329	0.204
BP × ESJ	-0.114	0.131	-0.866	0.387	-0.371	0.144
Intercept	2.726	0.155	17.559	0.000	2.421	3.030

Note. WP is coded such that 1 = Encounter and 0 = No Encounter. Emotion was rated on a 1 (not at all) to 7 (extremely) scale.

Supplementary Table 70. Full regression results for analysis of system-directed anger after poor encounter in Study 6.

Predictor	B	SE B	*z*	*p*	CI LB	CI UB
Econ. System Justification (ESJ)	-0.446	0.149	-2.989	0.003	-0.739	-0.154
Within Poor Encounter (WP)	1.092	0.152	7.164	0.000	0.793	1.391
**WP × ESJ**	**-0.361**	**0.152**	**-2.380**	**0.017**	**-0.658**	**-0.064**
Between Poor Encounter (BP)	-0.027	0.153	-0.178	0.858	-0.327	0.272
BP × ESJ	-0.073	0.147	-0.494	0.621	-0.361	0.215
Intercept	2.425	0.148	16.340	0.000	2.134	2.715

Note. WP is coded such that 1 = Encounter and 0 = No Encounter. Emotion was rated on a 1 (not at all) to 7 (extremely) scale.

Supplementary Table 71. Full regression results for analysis of system-directed disgust after poor encounter in Study 6.

Predictor	B	SE B	*z*	*p*	CI LB	CI UB
Econ. System Justification (ESJ)	-0.447	0.134	-3.327	0.001	-0.711	-0.184
Within Poor Encounter (WP)	1.069	0.149	7.191	0.000	0.777	1.360
**WP × ESJ**	**-0.482**	**0.148**	**-3.260**	**0.001**	**-0.771**	**-0.192**
Between Poor Encounter (BP)	0.008	0.141	0.055	0.956	-0.269	0.285
BP × ESJ	-0.100	0.136	-0.740	0.459	-0.366	0.165
Intercept	2.331	0.134	17.423	0.000	2.069	2.594

Note. WP is coded such that 1 = Encounter and 0 = No Encounter. Emotion was rated on a 1 (not at all) to 7 (extremely) scale.

Supplementary Table 72. Full regression results for analysis of person-directed empathy after rich encounter in Study 6.

Predictor	B	SE B	*z*	*p*	CI LB	CI UB
Econ. System Justification (ESJ)	0.122	0.127	0.962	0.336	-0.127	0.372
Within Rich Encounter (WR)	0.282	0.177	1.590	0.112	-0.066	0.629
**WR × ESJ**	**0.104**	**0.174**	**0.598**	**0.550**	**-0.237**	**0.445**
Between Rich Encounter (BR)	0.270	0.116	2.324	0.020	0.042	0.498
BR × ESJ	-0.162	0.123	-1.322	0.186	-0.402	0.078
Intercept	1.950	0.127	15.395	0.000	1.701	2.198

Note. WR is coded such that 1 = Encounter and 0 = No Encounter. Emotion was rated on a 1 (not at all) to 7 (extremely) scale.

Supplementary Table 73. Full regression results for analysis of person-directed anger after rich encounter in Study 6.

Predictor	B	SE B	*z*	*p*	CI LB	CI UB
Econ. System Justification (ESJ)	0.001	0.149	0.010	0.992	-0.290	0.293
Within Rich Encounter (WR)	0.418	0.180	2.327	0.020	0.066	0.770
**WR × ESJ**	**-0.408**	**0.176**	**-2.317**	**0.021**	**-0.753**	**-0.063**
Between Rich Encounter (BR)	-0.009	0.135	-0.069	0.945	-0.273	0.255
BR × ESJ	0.010	0.143	0.072	0.942	-0.269	0.290
Intercept	1.947	0.148	13.180	0.000	1.657	2.236

Note. WR is coded such that 1 = Encounter and 0 = No Encounter. Emotion was rated on a 1 (not at all) to 7 (extremely) scale.

Supplementary Table 74. Full regression results for analysis of self-directed sadness after rich encounter in Study 6.

Predictor	B	SE B	*z*	*p*	CI LB	CI UB
Econ. System Justification (ESJ)	0.029	0.148	0.197	0.844	-0.260	0.319
Within Rich Encounter (WR)	0.188	0.150	1.254	0.210	-0.106	0.481
**WR × ESJ**	**-0.335**	**0.147**	**-2.284**	**0.022**	**-0.622**	**-0.048**
Between Rich Encounter (BR)	0.108	0.137	0.794	0.427	-0.159	0.376
BR × ESJ	-0.064	0.145	-0.445	0.657	-0.348	0.219
Intercept	2.106	0.146	14.381	0.000	1.819	2.393

Note. WR is coded such that 1 = Encounter and 0 = No Encounter. Emotion was rated on a 1 (not at all) to 7 (extremely) scale.

Supplementary Table 75. Full regression results for analysis of jealousy following rich after in Study 6.

Predictor	B	SE B	*z*	*p*	CI LB	CI UB
Econ. System Justification (ESJ)	0.187	0.152	1.229	0.219	-0.111	0.486
Within Rich Encounter (WR)	0.631	0.185	3.410	0.001	0.268	0.993
**WR × ESJ**	**-0.445**	**0.181**	**-2.459**	**0.014**	**-0.801**	**-0.090**
Between Rich Encounter (BR)	0.271	0.145	1.867	0.062	-0.013	0.556
BR × ESJ	0.073	0.153	0.473	0.636	-0.228	0.373
Intercept	2.352	0.151	15.539	0.000	2.055	2.648

Note. WR is coded such that 1 = Encounter and 0 = No Encounter. Emotion was rated on a 1 (not at all) to 7 (extremely) scale.

Supplementary Table 76. Full regression results for analysis of system-directed anger after rich encounter in Study 6.

Predictor	B	SE B	*z*	*p*	CI LB	CI UB
Econ. System Justification (ESJ)	-0.258	0.145	-1.784	0.074	-0.541	0.025
Within Rich Encounter (WR)	0.549	0.188	2.915	0.004	0.180	0.918
**WR × ESJ**	**-0.488**	**0.185**	**-2.645**	**0.008**	**-0.850**	**-0.126**
Between Rich Encounter (BR)	-0.030	0.138	-0.217	0.829	-0.301	0.241
BR × ESJ	-0.114	0.146	-0.785	0.433	-0.400	0.171
Intercept	2.178	0.144	15.152	0.000	1.896	2.460

Note. WR is coded such that 1 = Encounter and 0 = No Encounter. Emotion was rated on a 1 (not at all) to 7 (extremely) scale.

Supplementary Table 77. Full regression results for analysis of system-directed disgust after rich encounter in Study 6.

Predictor	B	SE B	*z*	*p*	CI LB	CI UB
Econ. System Justification (ESJ)	-0.258	0.150	-1.716	0.086	-0.552	0.037
Within Rich Encounter (WR)	0.502	0.195	2.572	0.010	0.119	0.884
**WR × ESJ**	**-0.415**	**0.191**	**-2.171**	**0.030**	**-0.789**	**-0.040**
Between Rich Encounter (BR)	-0.004	0.143	-0.025	0.980	-0.284	0.277
BR × ESJ	-0.170	0.151	-1.124	0.261	-0.466	0.126
Intercept	2.228	0.149	14.919	0.000	1.936	2.521

Note. WR is coded such that 1 = Encounter and 0 = No Encounter. Emotion was rated on a 1 (not at all) to 7 (extremely) scale.

Supplementary Table 78. Robustness check for analysis person-directed anger after poor encounter in Study 6.

Predictor	B	SE B	*z*	*p*	CI LB	CI UB
Econ. System Justification (ESJ)	0.191	0.130	1.466	0.143	-0.064	0.447
Within Poor Encounter (WP)	0.076	0.112	0.679	0.497	-0.143	0.296
**WP × ESJ**	**-0.292**	**0.108**	**-2.703**	**0.007**	**-0.504**	**-0.080**
Between Poor Encounter (BP)	-0.039	0.116	-0.336	0.737	-0.266	0.188
BP × ESJ	0.028	0.108	0.261	0.794	-0.183	0.239
religiosity	0.053	0.048	1.114	0.265	-0.040	0.147
Black	0.293	0.569	0.515	0.607	-0.822	1.409
Latinx	0.119	0.326	0.365	0.715	-0.520	0.758
Asian	0.052	0.324	0.160	0.873	-0.582	0.686
Other	-0.467	0.487	-0.958	0.338	-1.422	0.488
Sex	-0.094	0.242	-0.390	0.696	-0.568	0.380
Income	0.046	0.044	1.056	0.291	-0.040	0.132
Intercept	1.161	0.378	3.071	0.002	0.420	1.903

Note. WP is coded such that 1 = Encounter and 0 = No Encounter. Emotion was rated on a 1 (not at all) to 7 (extremely) scale.

Supplementary Table 79. Robustness check for analysis system-directed anger after poor encounter in Study 6.

Predictor	B	SE B	*z*	*p*	CI LB	CI UB
Econ. System Justification (ESJ)	-0.441	0.157	-2.803	0.005	-0.750	-0.133
Within Poor Encounter (WP)	1.089	0.163	6.686	0.000	0.769	1.408
**WP × ESJ**	**-0.357**	**0.157**	**-2.272**	**0.023**	**-0.665**	**-0.049**
Between Poor Encounter (BP)	0.125	0.165	0.758	0.448	-0.198	0.449
BP × ESJ	-0.168	0.148	-1.134	0.257	-0.459	0.122
religiosity	0.083	0.063	1.320	0.187	-0.040	0.206
Black	0.837	0.723	1.157	0.247	-0.581	2.255
Latinx	0.974	0.432	2.253	0.024	0.127	1.822
Asian	0.245	0.425	0.576	0.564	-0.588	1.077
Other	0.054	0.616	0.088	0.930	-1.154	1.262
Sex	0.257	0.325	0.790	0.429	-0.381	0.895
Income	-0.106	0.058	-1.831	0.067	-0.219	0.007
Intercept	2.342	0.496	4.721	0.000	1.370	3.314

Note. WP is coded such that 1 = Encounter and 0 = No Encounter. Emotion was rated on a 1 (not at all) to 7 (extremely) scale.

Supplementary Table 80. Robustness check for analysis system-directed disgust after poor encounter in Study 6.

Predictor	B	SE B	*z*	*p*	CI LB	CI UB
Econ. System Justification (ESJ)	-0.426	0.143	-2.981	0.003	-0.707	-0.146
Within Poor Encounter (WP)	1.093	0.156	6.986	0.000	0.786	1.399
**WP × ESJ**	**-0.477**	**0.151**	**-3.160**	**0.002**	**-0.773**	**-0.181**
Between Poor Encounter (BP)	0.152	0.153	0.988	0.323	-0.149	0.452
BP × ESJ	-0.183	0.137	-1.337	0.181	-0.451	0.085
religiosity	0.056	0.057	0.973	0.330	-0.056	0.167
Black	0.918	0.655	1.402	0.161	-0.365	2.201
Latinx	0.790	0.395	2.000	0.046	0.016	1.563
Asian	0.263	0.386	0.681	0.496	-0.494	1.020
Other	0.013	0.557	0.023	0.982	-1.080	1.105
Sex	0.287	0.298	0.963	0.335	-0.297	0.870
Income	-0.118	0.053	-2.240	0.025	-0.221	-0.015
Intercept	2.414	0.451	5.347	0.000	1.529	3.299

Note. WP is coded such that 1 = Encounter and 0 = No Encounter. Emotion was rated on a 1 (not at all) to 7 (extremely) scale.

Supplementary Table 81. Robustness check for analysis person-directed anger after rich encounter in Study 6.

Predictor	B	SE B	*z*	*p*	CI LB	CI UB
Econ. System Justification (ESJ)	-0.085	0.168	-0.507	0.612	-0.414	0.244
Within Rich Encounter (WR)	0.480	0.189	2.536	0.011	0.109	0.851
**WR × ESJ**	**-0.423**	**0.180**	**-2.357**	**0.018**	**-0.775**	**-0.071**
Between Rich Encounter (BR)	-0.081	0.144	-0.567	0.571	-0.363	0.200
BR × ESJ	-0.013	0.150	-0.090	0.928	-0.307	0.280
religiosity	0.060	0.063	0.959	0.338	-0.063	0.184
Black	0.110	0.734	0.150	0.881	-1.329	1.549
Latinx	0.230	0.424	0.541	0.589	-0.602	1.061
Asian	0.217	0.421	0.516	0.606	-0.609	1.043
Other	0.169	0.605	0.280	0.780	-1.017	1.355
Sex	-0.433	0.322	-1.348	0.178	-1.064	0.197
Income	-0.046	0.058	-0.805	0.421	-0.159	0.066
Intercept	2.159	0.491	4.400	0.000	1.197	3.120

Note. WR is coded such that 1 = Encounter and 0 = No Encounter. Emotion was rated on a 1 (not at all) to 7 (extremely) scale.

Supplementary Table 82. Robustness check for analysis self-directed sadness after rich encounter in Study 6.

Predictor	B	SE B	*z*	*p*	CI LB	CI UB
Econ. System Justification (ESJ)	0.044	0.166	0.267	0.790	-0.281	0.369
Within Rich Encounter (WR)	0.226	0.158	1.437	0.151	-0.082	0.535
**WR × ESJ**	**-0.354**	**0.149**	**-2.373**	**0.018**	**-0.647**	**-0.062**
Between Rich Encounter (BR)	0.040	0.140	0.286	0.775	-0.234	0.315
BR × ESJ	-0.024	0.146	-0.164	0.869	-0.311	0.263
religiosity	0.043	0.063	0.690	0.490	-0.080	0.166
Black	0.143	0.732	0.196	0.845	-1.291	1.577
Latinx	0.823	0.422	1.948	0.051	-0.005	1.650
Asian	0.034	0.419	0.080	0.936	-0.788	0.855
Other	-0.092	0.605	-0.152	0.879	-1.277	1.093
Sex	0.194	0.319	0.610	0.542	-0.430	0.819
Income	-0.096	0.057	-1.684	0.092	-0.209	0.016
Intercept	2.240	0.488	4.592	0.000	1.284	3.197

Note. WR is coded such that 1 = Encounter and 0 = No Encounter. Emotion was rated on a 1 (not at all) to 7 (extremely) scale.

Supplementary Table 83. Robustness check for analysis jealousy after rich encounter in Study 6.

Predictor	B	SE B	*z*	*p*	CI LB	CI UB
Econ. System Justification (ESJ)	0.213	0.177	1.203	0.229	-0.134	0.559
Within Rich Encounter (WR)	0.670	0.196	3.425	0.001	0.287	1.054
**WR × ESJ**	**-0.455**	**0.186**	**-2.452**	**0.014**	**-0.819**	**-0.091**
Between Rich Encounter (BR)	0.229	0.155	1.474	0.141	-0.076	0.533
BR × ESJ	0.086	0.162	0.530	0.596	-0.232	0.404
religiosity	-0.004	0.068	-0.060	0.952	-0.137	0.129
Black	0.078	0.793	0.098	0.922	-1.477	1.633
Latinx	0.458	0.462	0.993	0.321	-0.446	1.363
Asian	-0.086	0.455	-0.190	0.850	-0.978	0.805
Other	0.059	0.655	0.090	0.928	-1.226	1.343
Sex	-0.070	0.349	-0.201	0.841	-0.755	0.615
Income	-0.082	0.062	-1.321	0.187	-0.204	0.040
Intercept	2.852	0.528	5.402	0.000	1.817	3.886

Note. BR is coded such that 1 = Encounter and 0 = No Encounter. Emotion was rated on a 1 (not at all) to 7 (extremely) scale.

Supplementary Table 84. Robustness check for analysis system-directed anger after rich encounter in Study 6.

Predictor	B	SE B	*z*	*p*	CI LB	CI UB
Econ. System Justification (ESJ)	-0.258	0.167	-1.546	0.122	-0.584	0.069
Within Rich Encounter (WR)	0.571	0.203	2.817	0.005	0.174	0.969
**WR × ESJ**	**-0.488**	**0.192**	**-2.536**	**0.011**	**-0.865**	**-0.111**
Between Rich Encounter (BR)	-0.081	0.148	-0.548	0.584	-0.371	0.209
BR × ESJ	-0.082	0.155	-0.532	0.595	-0.385	0.221
religiosity	0.036	0.064	0.568	0.570	-0.089	0.162
Black	-0.250	0.746	-0.334	0.738	-1.712	1.213
Latinx	0.300	0.435	0.691	0.490	-0.552	1.153
Asian	-0.026	0.428	-0.061	0.951	-0.865	0.813
Other	-0.188	0.615	-0.305	0.760	-1.394	1.018
Sex	-0.001	0.330	-0.002	0.999	-0.647	0.646
Income	-0.085	0.059	-1.449	0.147	-0.200	0.030
Intercept	2.524	0.497	5.081	0.000	1.550	3.498

Note. WR is coded such that 1 = Encounter and 0 = No Encounter. Emotion was rated on a 1 (not at all) to 7 (extremely) scale.

Supplementary Table 85. Robustness check for analysis system-directed disgust after rich encounter in Study 6.

Predictor	B	SE B	*z*	*p*	CI LB	CI UB
Econ. System Justification (ESJ)	-0.282	0.172	-1.638	0.101	-0.620	0.056
Within Rich Encounter (WR)	0.556	0.209	2.660	0.008	0.146	0.965
**WR × ESJ**	**-0.428**	**0.198**	**-2.162**	**0.031**	**-0.817**	**-0.040**
Between Rich Encounter (BR)	-0.058	0.153	-0.380	0.704	-0.359	0.242
BR × ESJ	-0.144	0.160	-0.899	0.368	-0.458	0.170
religiosity	0.051	0.066	0.771	0.441	-0.079	0.181
Black	-0.260	0.774	-0.335	0.737	-1.778	1.258
Latinx	0.191	0.452	0.423	0.672	-0.694	1.076
Asian	0.074	0.444	0.167	0.868	-0.797	0.945
Other	-0.311	0.639	-0.487	0.626	-1.563	0.941
Sex	-0.103	0.343	-0.301	0.763	-0.775	0.568
Income	-0.077	0.061	-1.268	0.205	-0.196	0.042
Intercept	2.543	0.515	4.936	0.000	1.534	3.553

Note. WR is coded such that 1 = Encounter and 0 = No Encounter. Emotion was rated on a 1 (not at all) to 7 (extremely) scale.
