# Supplementary material for: Economic system justification predicts muted emotional responses to inequality
Source: Nat Commun. 2020 Jan 20;11:383. doi: 10.1038/s41467-019-14193-z (PMC6971272; doi:10.1038/s41467-019-14193-z)
Supplement: Supplementary file 3 — Reporting Summary [file 41467_2019_14193_MOESM3_ESM.pdf]

nature research

Corresponding author(s): Shahrzad Goudarzi

Last updated by author(s): 2019, 11.12

Reporting Summary

Nature Research wishes to improve the reproducibility of the work that we publish. This form provides structure for consistency and transparency in reporting. For further information on Nature Research policies, see Authors & Referees and the Editorial Policy Checklist .

Statistics

For all statistical analyses, confirm that the following items are present in the figure legend, table legend, main text, or Methods section.

n/a	Confirmed	
☐	☒	The exact sample size (*n*) for each experimental group/condition, given as a discrete number and unit of measurement
☐	☒	A statement on whether measurements were taken from distinct samples or whether the same sample was measured repeatedly
☐	☒	The statistical test(s) used AND whether they are one- or two-sided<br>*Only common tests should be described solely by name; describe more complex techniques in the Methods section.*
☐	☒	A description of all covariates tested
☐	☒	A description of any assumptions or corrections, such as tests of normality and adjustment for multiple comparisons
☐	☒	A full description of the statistical parameters including central tendency (e.g. means) or other basic estimates (e.g. regression coefficient) AND variation (e.g. standard deviation) or associated estimates of uncertainty (e.g. confidence intervals)
☐	☒	For null hypothesis testing, the test statistic (e.g. *F*, *t*, *r*) with confidence intervals, effect sizes, degrees of freedom and *P* value noted<br>*Give P values as exact values whenever suitable.*
☒	☐	For Bayesian analysis, information on the choice of priors and Markov chain Monte Carlo settings
☒	☐	For hierarchical and complex designs, identification of the appropriate level for tests and full reporting of outcomes
☒	☐	Estimates of effect sizes (e.g. Cohen's *d*, Pearson's *r*), indicating how they were calculated

Our web collection on statistics for biologists contains articles on many of the points above.

Software and code

Policy information about availability of computer code

Data collection	Eprime 2.0, AcqKnowledge 4.3,
Data analysis	Stata/SE 15.0, Analysis code is deposited in the Open Science Framework repository. (https://osf.io/2qn2z/)

For manuscripts utilizing custom algorithms or software that are central to the research but not yet described in published literature, software must be made available to editors/reviewers. We strongly encourage code deposition in a community repository (e.g. GitHub). See the Nature Research guidelines for submitting code & software for further information.

Data

Policy information about availability of data

All manuscripts must include a data availability statement. This statement should provide the following information, where applicable:

- Accession codes, unique identifiers, or web links for publicly available datasets
- A list of figures that have associated raw data
- A description of any restrictions on data availability

The data sets for all studies are available in the Open Science Framework repository. (https://osf.io/2qn2z/)

Field-specific reporting

Please select the one below that is the best fit for your research. If you are not sure, read the appropriate sections before making your selection.

☐ Life sciences ☒ Behavioural & social sciences ☐ Ecological, evolutionary & environmental sciences

For a reference copy of the document with all sections, see nature.com/documents/nr-reporting-summary-flat.pdf

Behavioural & social sciences study design

All studies must disclose on these points even when the disclosure is negative.

Study description	Quantitative Experimental
Research sample	Study 1: 105 U.S.-born workers from Amazon's Mechanical Turk crowdsourcing platform (55 males, 50 females, aged 18 to 68, M = 37.72, SD = 13.03).<br><br>Study 2: 326 U.S.-born workers from the Prolific Academic crowdsourcing platform (151 men, 171 women, and 4 who identified as non-binary, aged 18 to 72, M = 33.80, SD = 11.77).<br><br>Study 3: 42 New York University undergraduates (NYU) born or raised in the U.S (18 males, 24 females, aged 18 to 31 years, M = 19.88, SD = 2.63).<br><br>Study 4: 37 New York University undergraduates (NYU) born or raised in the U.S (13 males, 24 females, ranging in age from 18 to 40, M = 19.81, SD = 3.72).<br><br>Study 5: 76 New York University undergraduates (NYU) born or raised in the U.S (19 males, 57 females; ranging in age from 17 to 24, M = 19.14, SD = 1.16).<br><br>Study 6: 71 NYU undergraduate participants (NYU) born or raised in the U.S (24 males, 47 females) ranging in age from 17.5 to 22.4 (M = 19.24, SD = 1.12).<br><br>In Study 1 and 2, we aimed on recruiting a demographically diverse range of participants that is relatively representative. Study 3–6, required lab sessions and/or involved longitudinal design and we recruited from NYU undergraduate psychology research participation pool due budget to limitation. Therefore, in these experiments, our sample is not representative.
Sampling strategy	Study 1: Convenience sampling, sample size was determined based on the maximum number of participants that can be recruited given the project's budget at the time of data collection. No sample-size computations were performed.<br><br>Study 2: Convenience sampling, sample size was informed by our previous study and the fact that we were adding a condition. We chose a relatively large sample size (n = 400) given that we were unsure of the attrition rate on Prolific. Sample size and rationale were preregistered on Open Science Framework. (https://osf.io/2qn2z/registrations). No sample-size computations were performed.<br><br>Study 3: Convenience sampling, the desired sample size (n = 60) was determine by the number of participants that could be recruited by the end of the spring 2015 academic semester. No sample-size computations were performed.<br><br>Study 4: Convenience sampling, we determined to run until we reach our desired sample size (n= 80) or until the end of the spring 2017 academic term. Desired sample size and rule for data collection termination were preregistered on Open Science Framework (https://osf.io/2qn2z/registrations). No sample-size computations were performed.<br><br>Study 5: Convenience sampling, We looked for 80% power to detect smallest relevant effect (from our previous study) at p less than .05. Sample size based on power analysis (n = 80) and rule for data collection termination were preregistered on Open Science Framework (https://osf.io/2qn2z/registrations).<br><br>Study 6: Convenience sampling, sample size was determined based on the maximum number of participants that can be recruited given the project's budget at the time of data collection. No sample-size computations were performed.
Data collection	Experiments 1–2: Qualtrics online survey distributed via the crowdsourcing platforms. No researcher was present.<br>Experiment 3–5: BIOPAC EMG100C and EDA100C modules, Qualtrics online survey completed on a Chromebook. A researcher (unaware to participants' ESJ scores) was present in an adjacent room and monitored the session using closed-circuit camera.
Timing	Study 1: Fall 2015 Academic Semester<br>Study 2: Fall 2018 Academic Semester<br>Study 3: Spring 2015 Academic Semester<br>Study 4: Fall 2017 Academic Semester<br>Study 5: Spring 2017–Fall 2017 Academic Semesters<br>Study 6: Spring 2018 Academic Semester
Data exclusions	Study 1–2: no data were excluded from analysis.<br>Study 3: Of the 54 participants originally recruited, 12 were excluded from preprocessing and analysis—3 due to audio and/or video malfunctions, 2 because of noncompliance with the experimenter's instructions, 2 because their pre-test data could not be located, 1 because impedance levels of her facial EMG electrodes could not be kept below 10 kΩ (based on recommended guidelines for EMG data collection), 1 who had an extreme emotional reaction during the session, and 3 because they were not born or raised in the United States despite our attempt at screening out those who were not born or raised in the U.S.<br>Study 4: Of the 39 participants originally recruited, 2 participants' corrugator and levator muscle activity data were excluded from

preprocessing and analysis because of high impedance (>10 kΩ). Data-exclusion criteria were preregistered on the Open Science Framework (OSF) (see https://osf.io/2qn2z/registrations).
Study 5: Of the 80 recruited participants, 4 participants' data were excluded from the preprocessing and analyses due to noncompliance with the experimenter's instructions. Data-exclusion criteria were preregistered on the Open Science Framework (OSF) (see https://osf.io/2qn2z/registrations.
Study 6: of the total 349 observations, 6 erroneous responses were removed from analysis. Results from all analysis reported in Study 6 are consistent with or without the exclusion of the erroneous responses. Results with the exclusion of these 6 responses are reported in the manuscript.

Non-participation	No participant dropped out/ declined participation in all experiments.
Randomization	Because our experimental manipulation was within participants in Studies 1–5, all participants were exposed to all conditions in all experiments.<br>Design of Study 6 was quasi-experimental and did not involve random assignment.

Reporting for specific materials, systems and methods

We require information from authors about some types of materials, experimental systems and methods used in many studies. Here, indicate whether each material, system or method listed is relevant to your study. If you are not sure if a list item applies to your research, read the appropriate section before selecting a response.

Materials & experimental systems

n/a	Involved in the study
☒	☐ Antibodies
☒	☐ Eukaryotic cell lines
☒	☐ Palaeontology
☒	☐ Animals and other organisms
☐	☒ Human research participants
☒	☐ Clinical data

Methods

n/a	Involved in the study
☒	☐ ChIP-seq
☒	☐ Flow cytometry
☒	☐ MRI-based neuroimaging

Human research participants

Policy information about studies involving human research participants

Population characteristics	See Above
Recruitment	Experiment 1: Amazon's Mechanical Turk crowdsourcing platform<br>Experiment 2: Prolific Academic crowdsourcing platform<br>Experiment 3–6: NYU Psychology Sona Systems<br>We do not know of any sources of possible self-selection.
Ethics oversight	NYU University Committee on Activities Involving Human Subjects(UCAIHS)

Note that full information on the approval of the study protocol must also be provided in the manuscript.
